# Supplementary material for: RAS Mediates BET Inhibitor-Endued Repression of Lymphoma Migration and Prognosticates a Novel Proteomics-Based Subgroup of DLBCL through Its Negative Regulator IQGAP3
Source: Cancers (Basel). 2021 Oct 7;13(19):5024. doi: 10.3390/cancers13195024 (PMC8508075; doi:10.3390/cancers13195024)
Supplement: Supplementary file 1 [file cancers-13-05024-s001.zip › cancers 1388839 Figure S6 original western blot.pptx]

## Slide 1
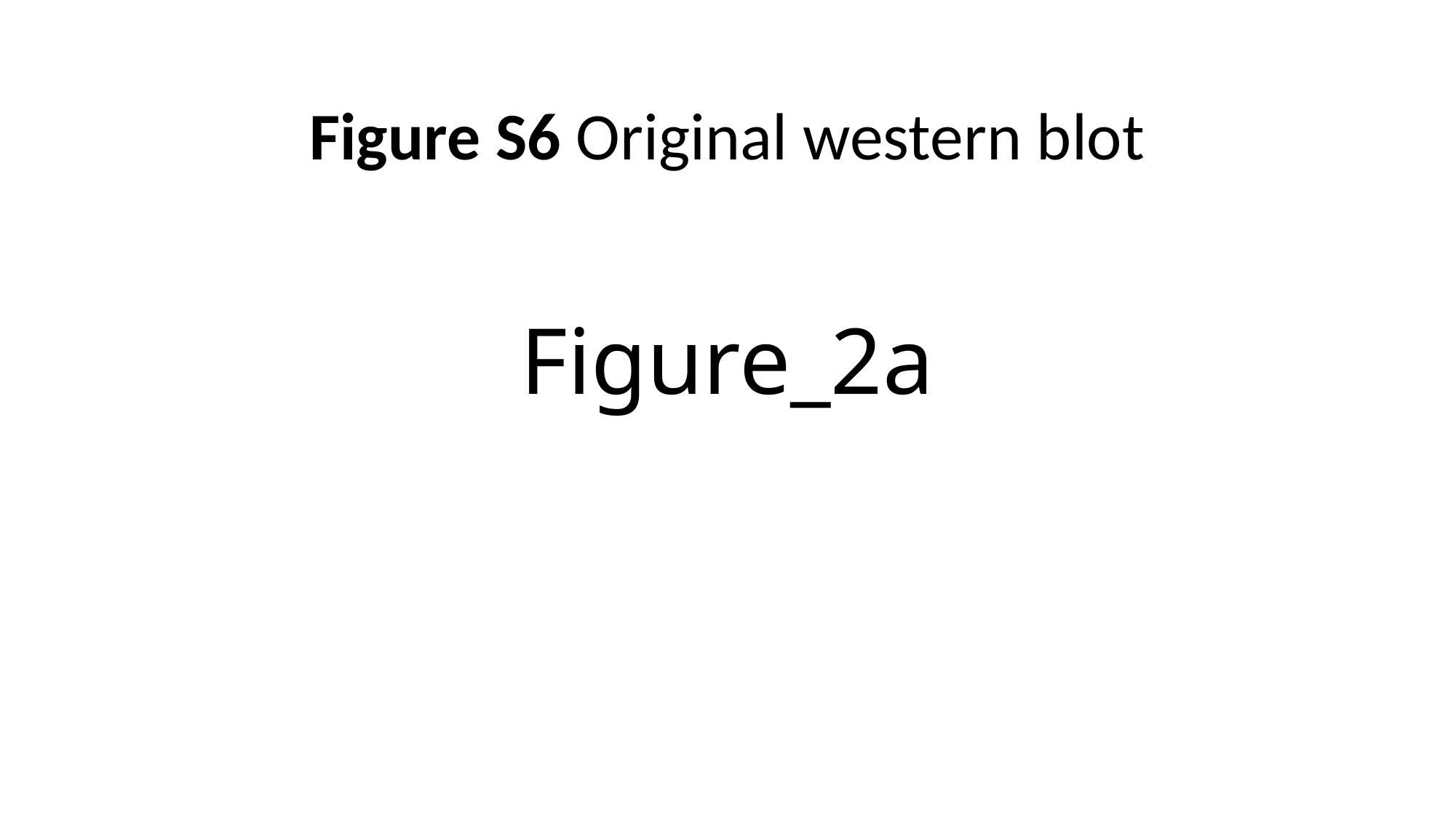

Figure S6 Original western blot
# Figure_2a

## Slide 2
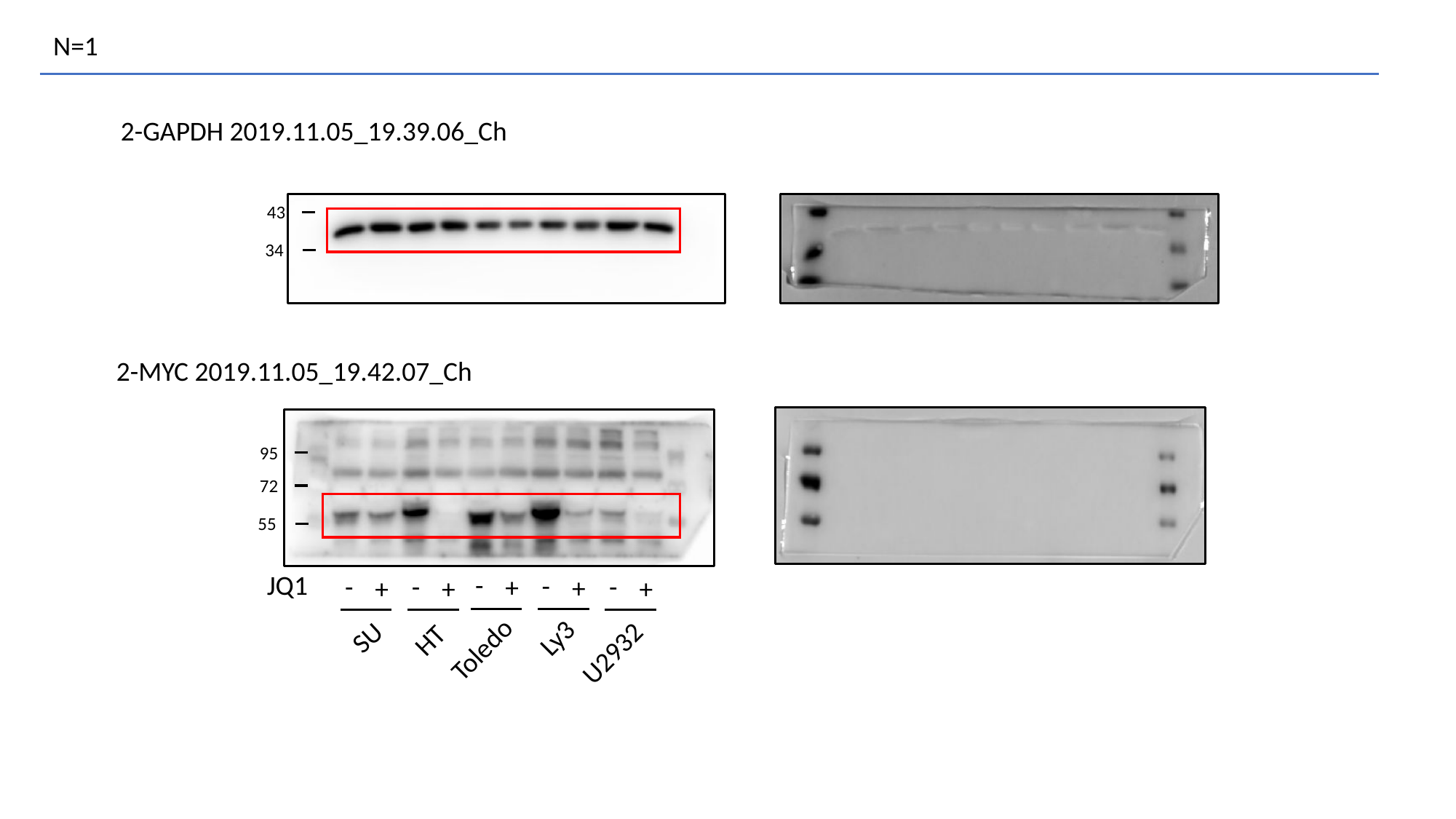

N=1
2-GAPDH 2019.11.05_19.39.06_Ch
43
34
2-MYC 2019.11.05_19.42.07_Ch
95
72
55
JQ1
-
-
-
-
-
+
+
+
+
+
Ly3
SU
HT
Toledo
U2932

## Slide 3
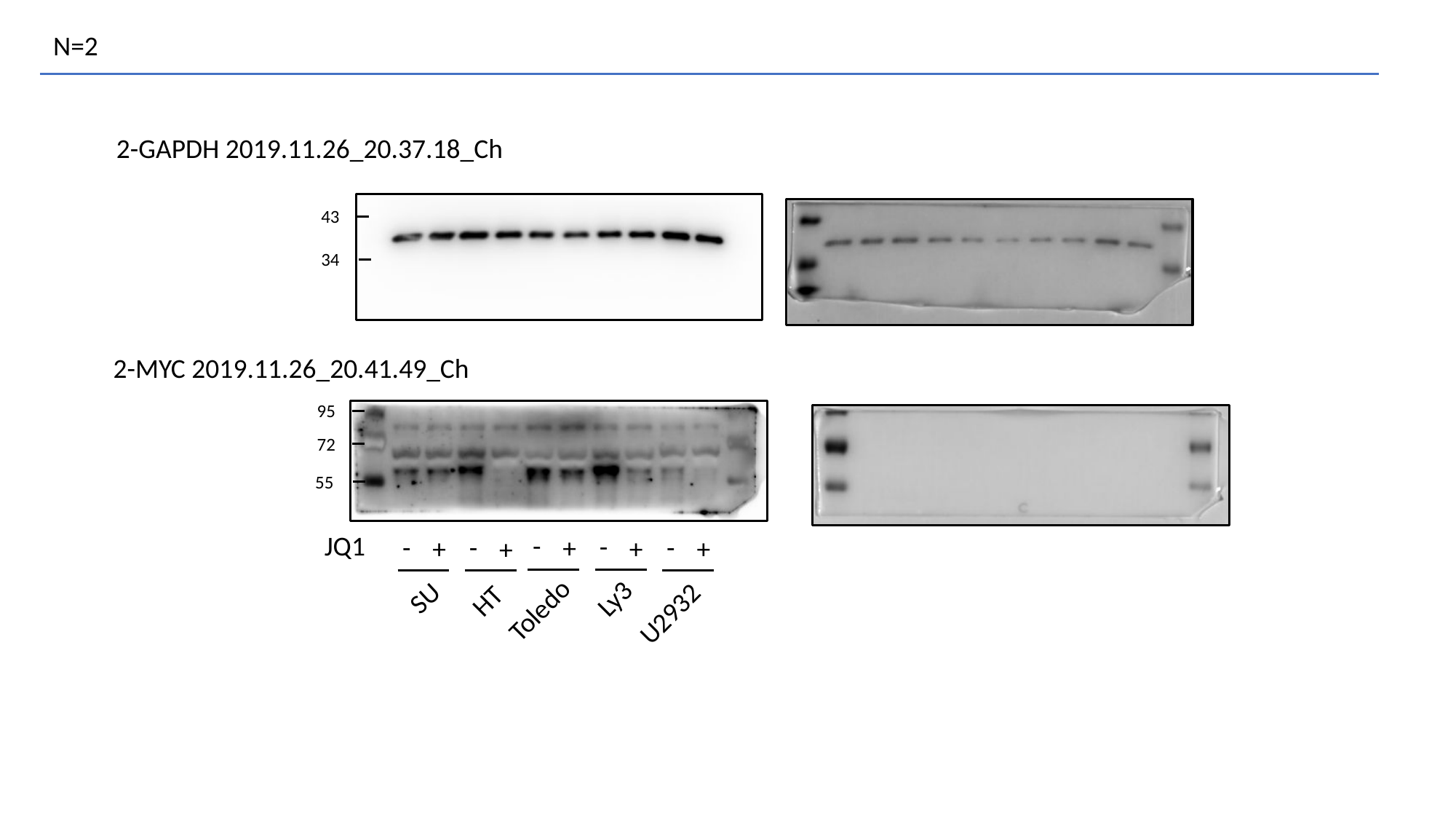

N=2
2-GAPDH 2019.11.26_20.37.18_Ch
43
34
2-MYC 2019.11.26_20.41.49_Ch
95
72
55
JQ1
-
-
-
-
-
+
+
+
+
+
Ly3
SU
HT
Toledo
U2932

## Slide 4
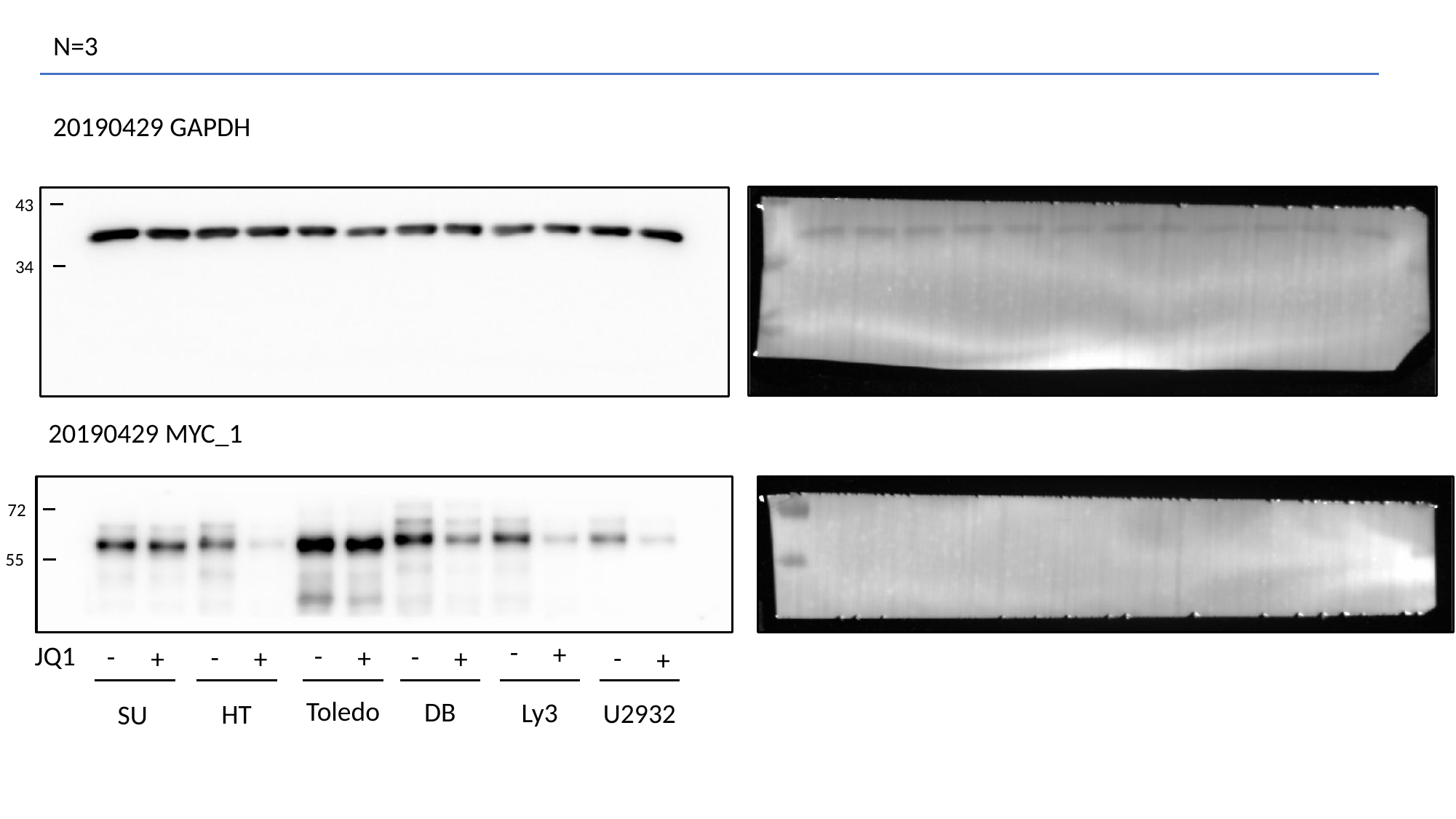

N=3
20190429 GAPDH
43
34
20190429 MYC_1
72
55
-
+
JQ1
-
-
-
-
-
+
+
+
+
+
Toledo
DB
Ly3
U2932
HT
SU

## Slide 5
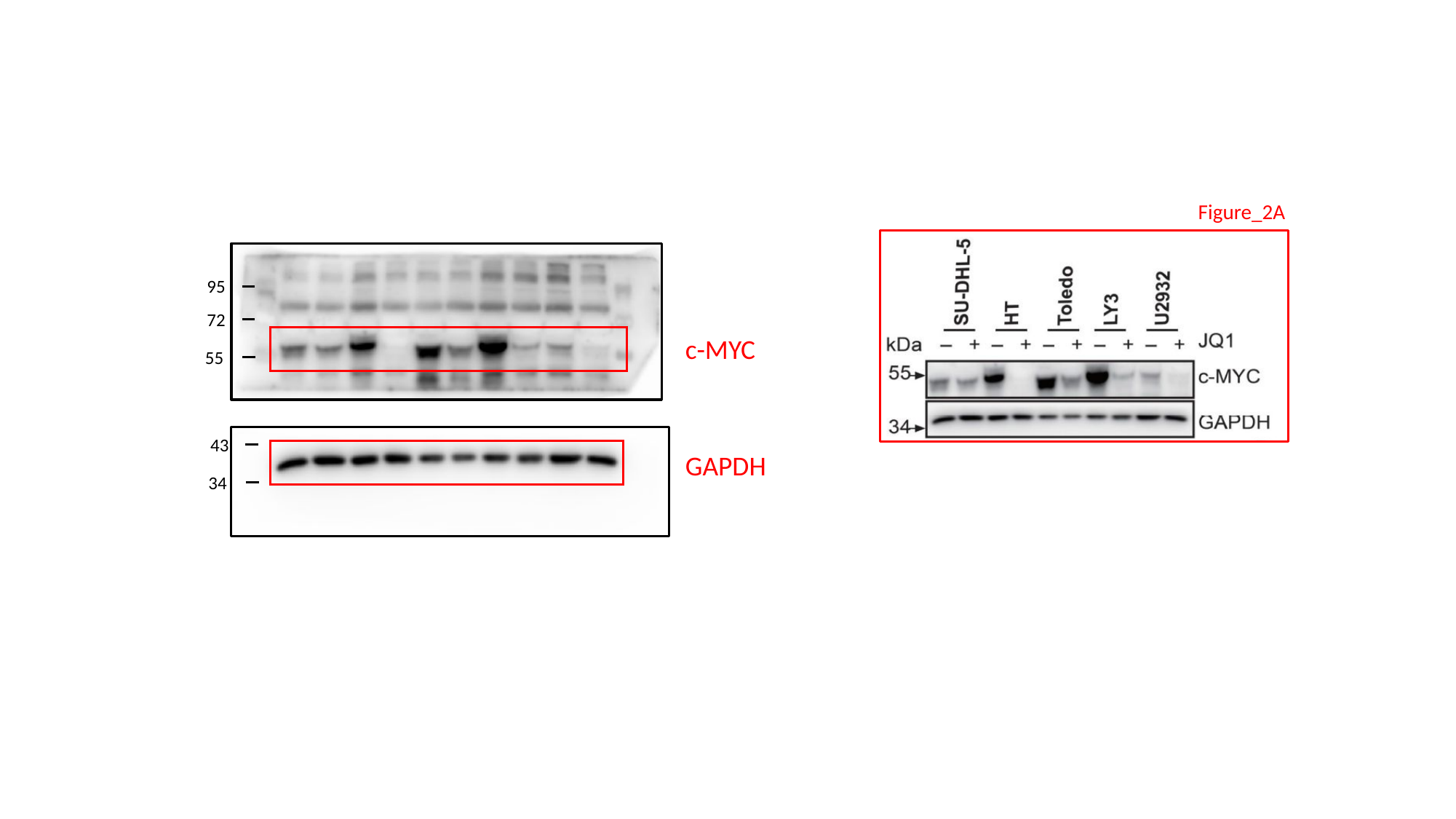

Figure_2A
95
72
c-MYC
55
43
GAPDH
34

## Slide 6
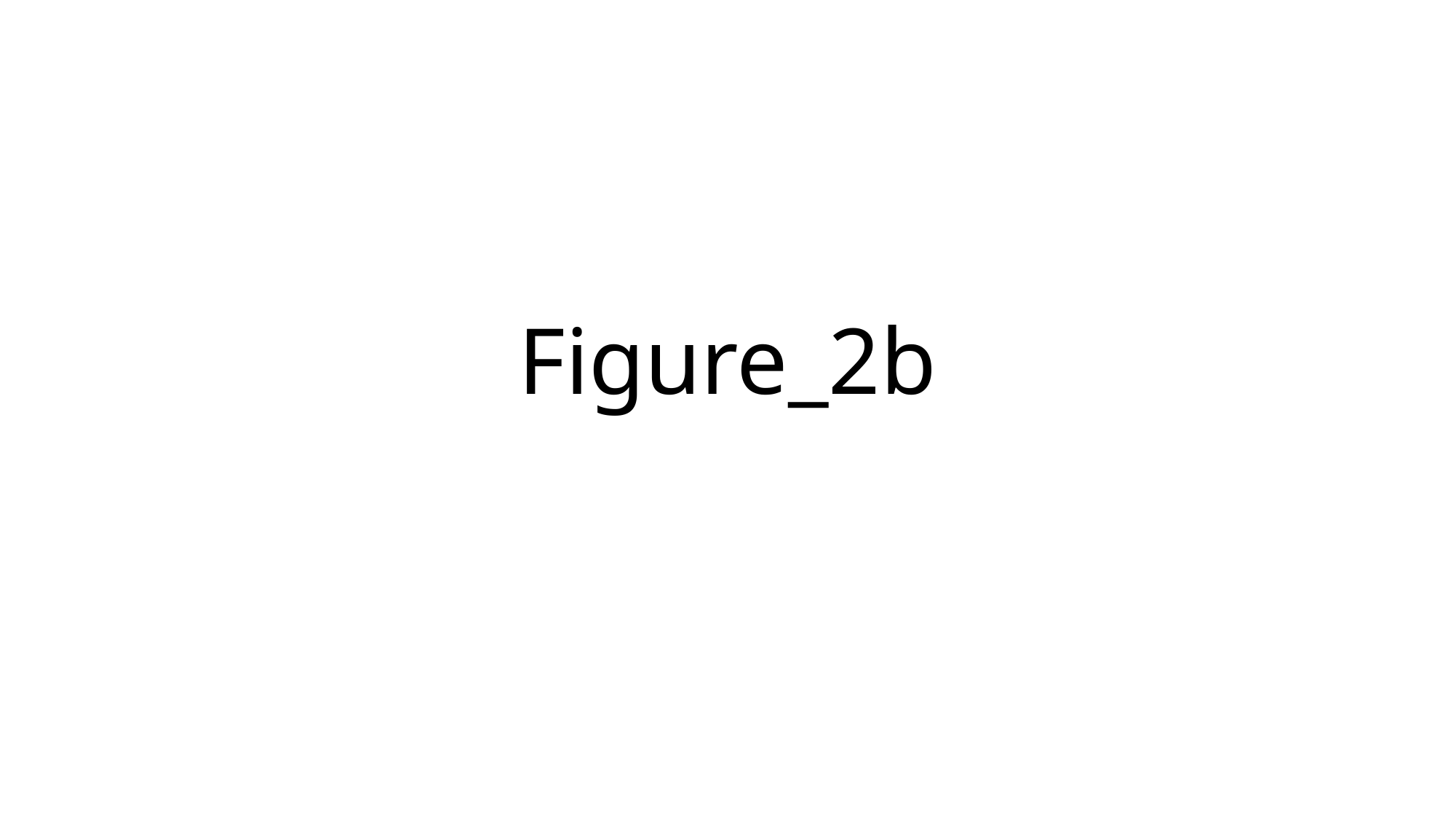

# Figure_2b

## Slide 7
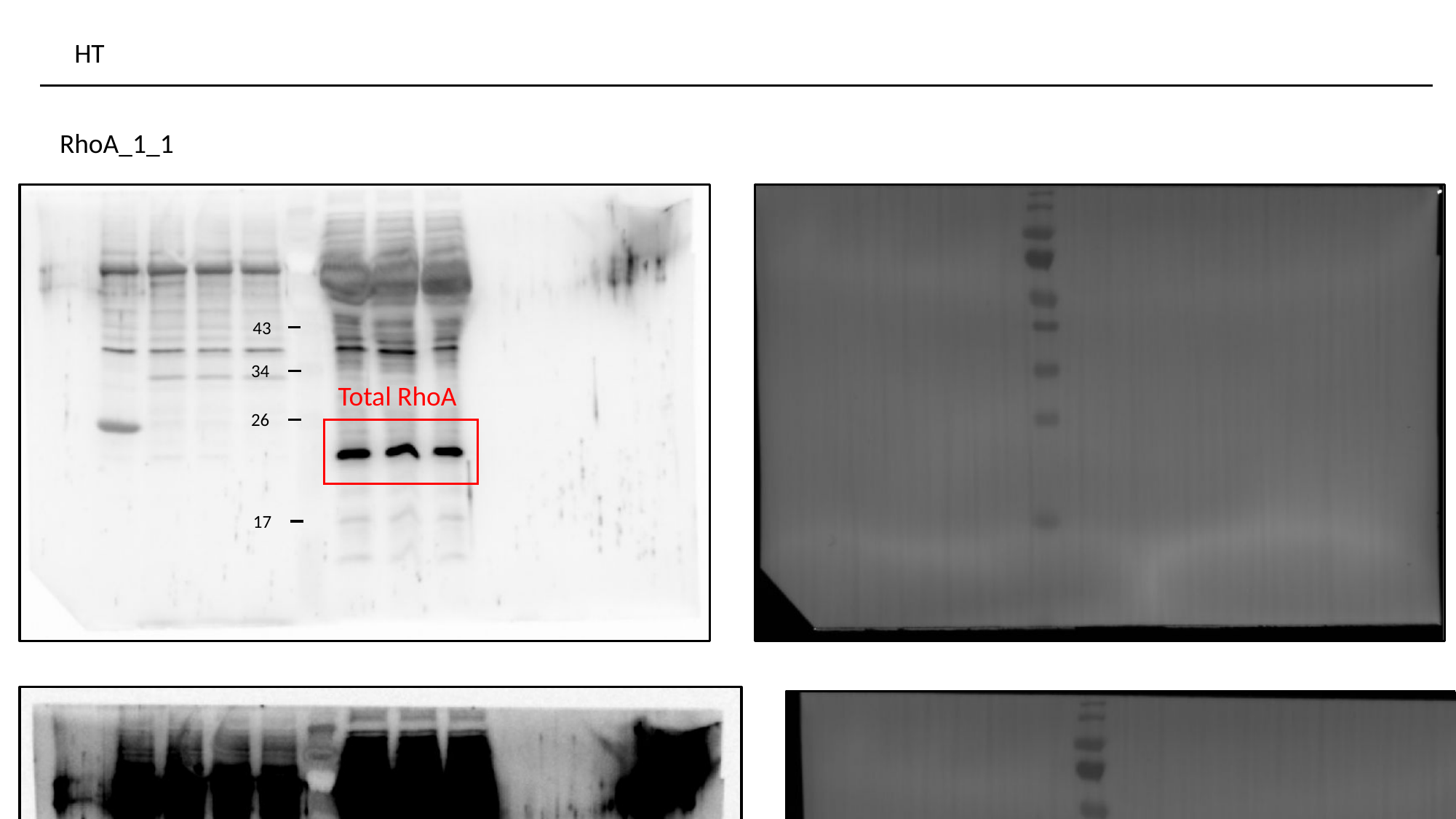

HT
RhoA_1_1
43
34
Total RhoA
26
17
43
34
26
GTP-RhoA
17

## Slide 8
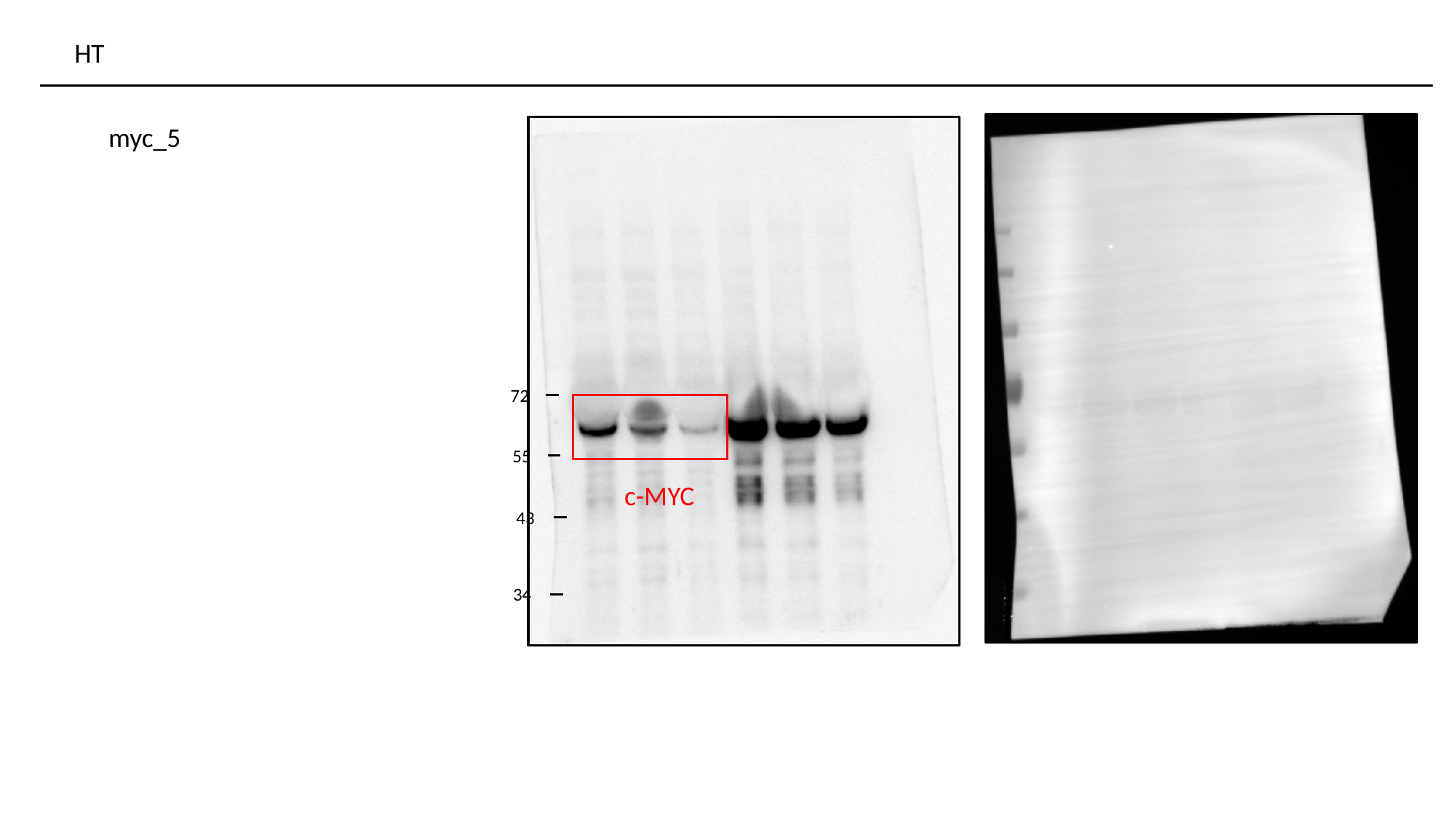

HT
myc_5
72
55
c-MYC
43
34

## Slide 9
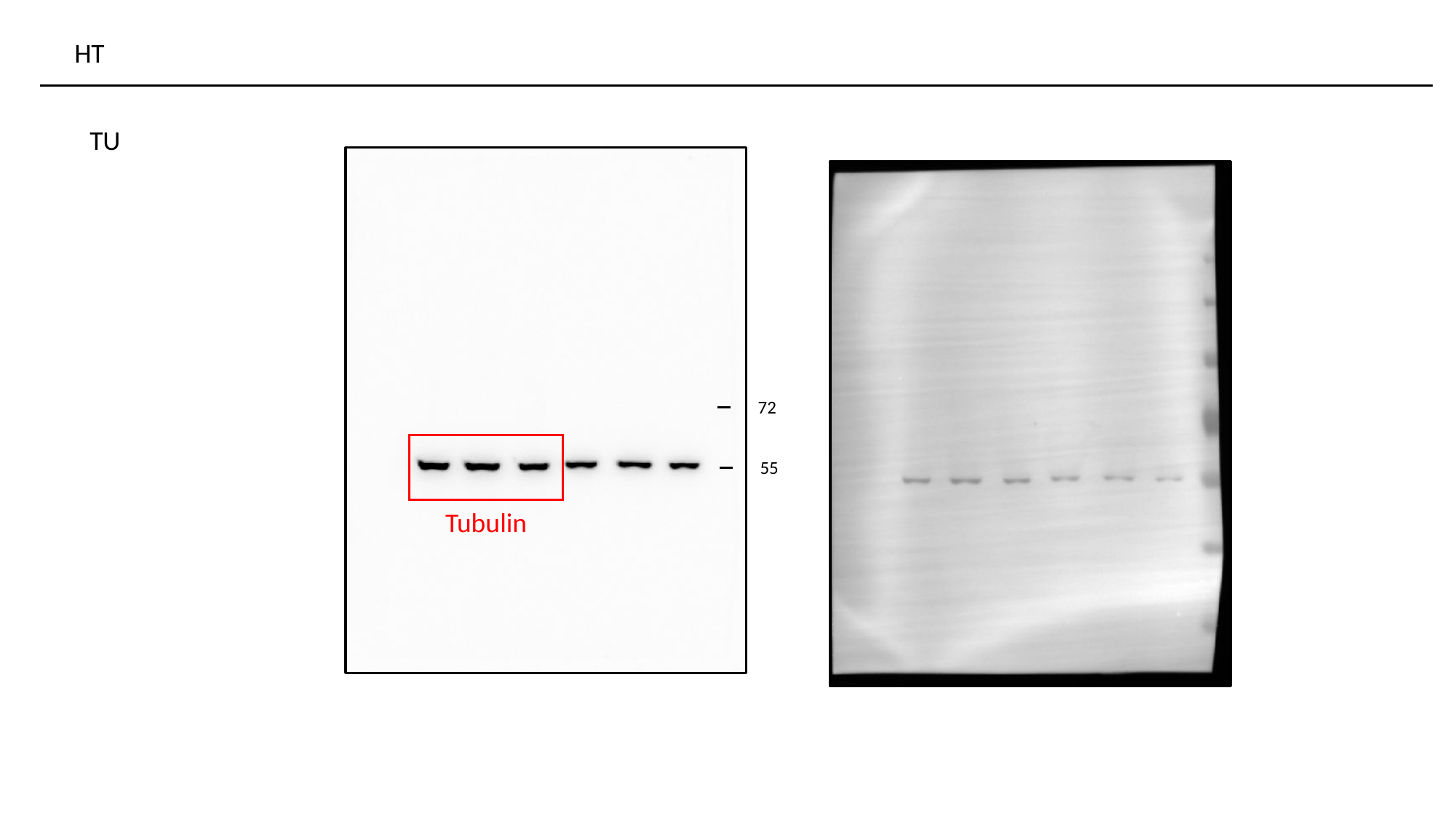

HT
TU
72
55
Tubulin

## Slide 10
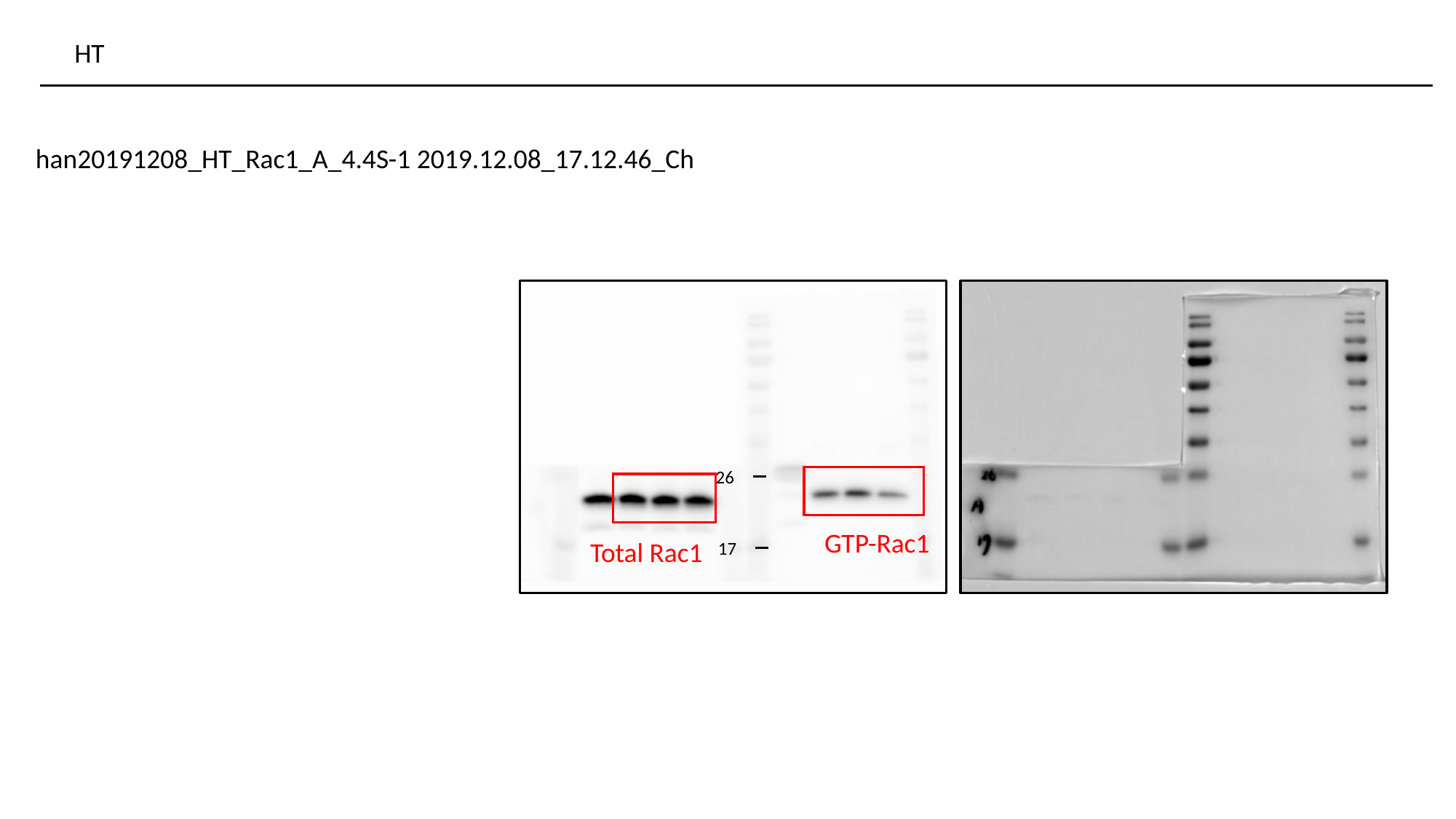

HT
han20191208_HT_Rac1_A_4.4S-1 2019.12.08_17.12.46_Ch
26
GTP-Rac1
Total Rac1
17

## Slide 11
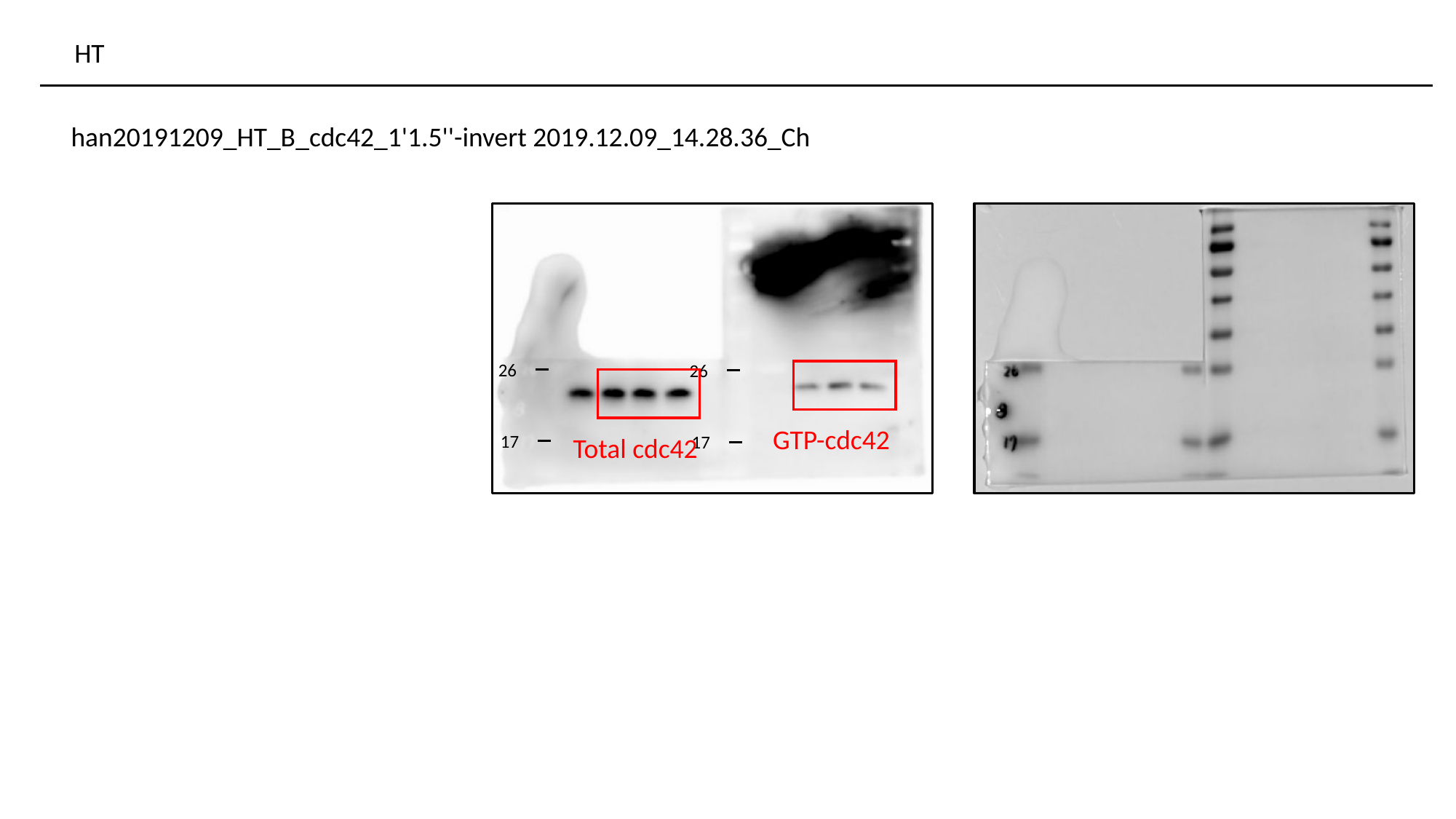

HT
han20191209_HT_B_cdc42_1'1.5''-invert 2019.12.09_14.28.36_Ch
26
26
GTP-cdc42
17
17
Total cdc42

## Slide 12
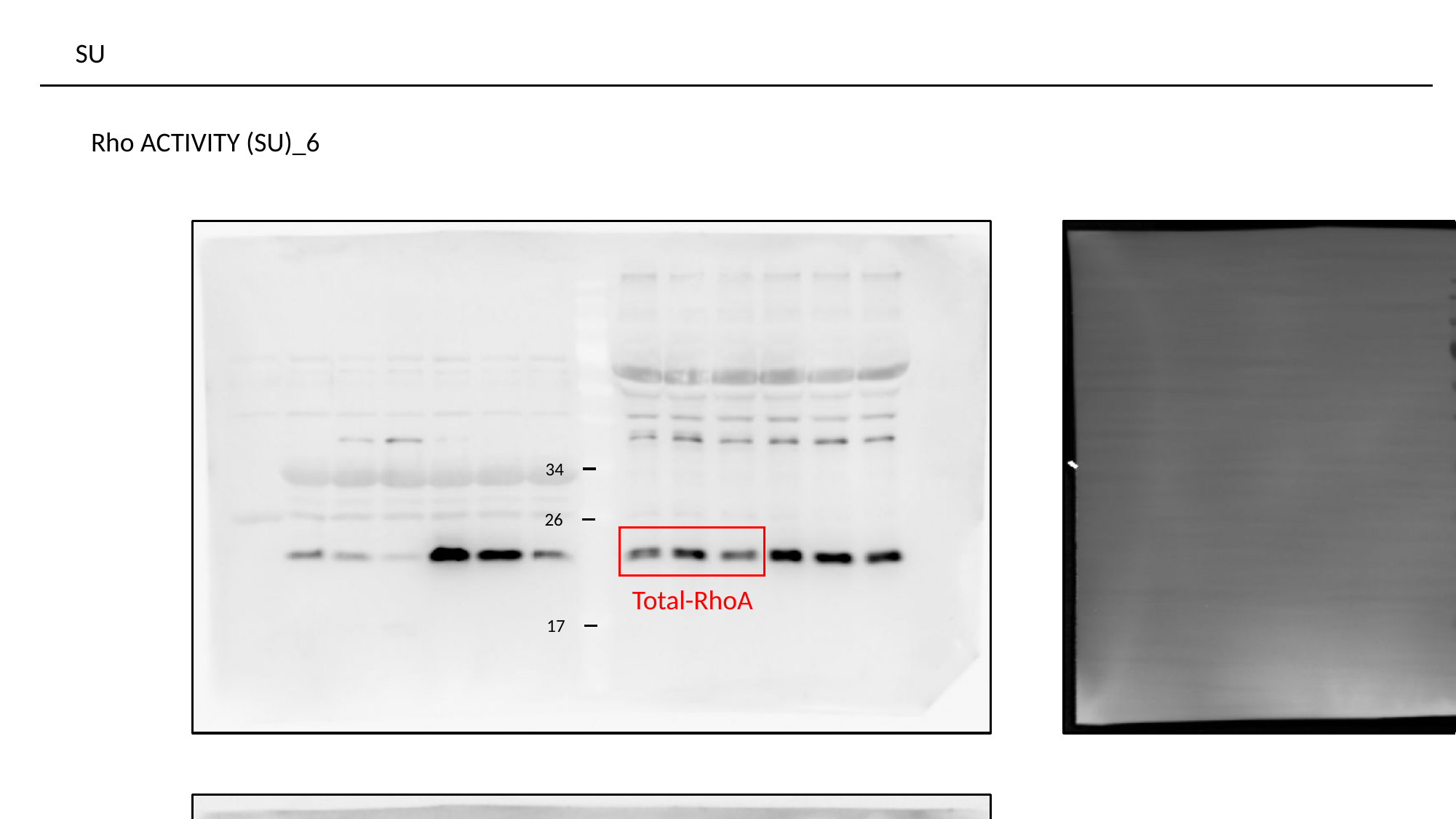

SU
Rho ACTIVITY (SU)_6
34
26
Total-RhoA
17
34
26
GTP-RhoA
17

## Slide 13
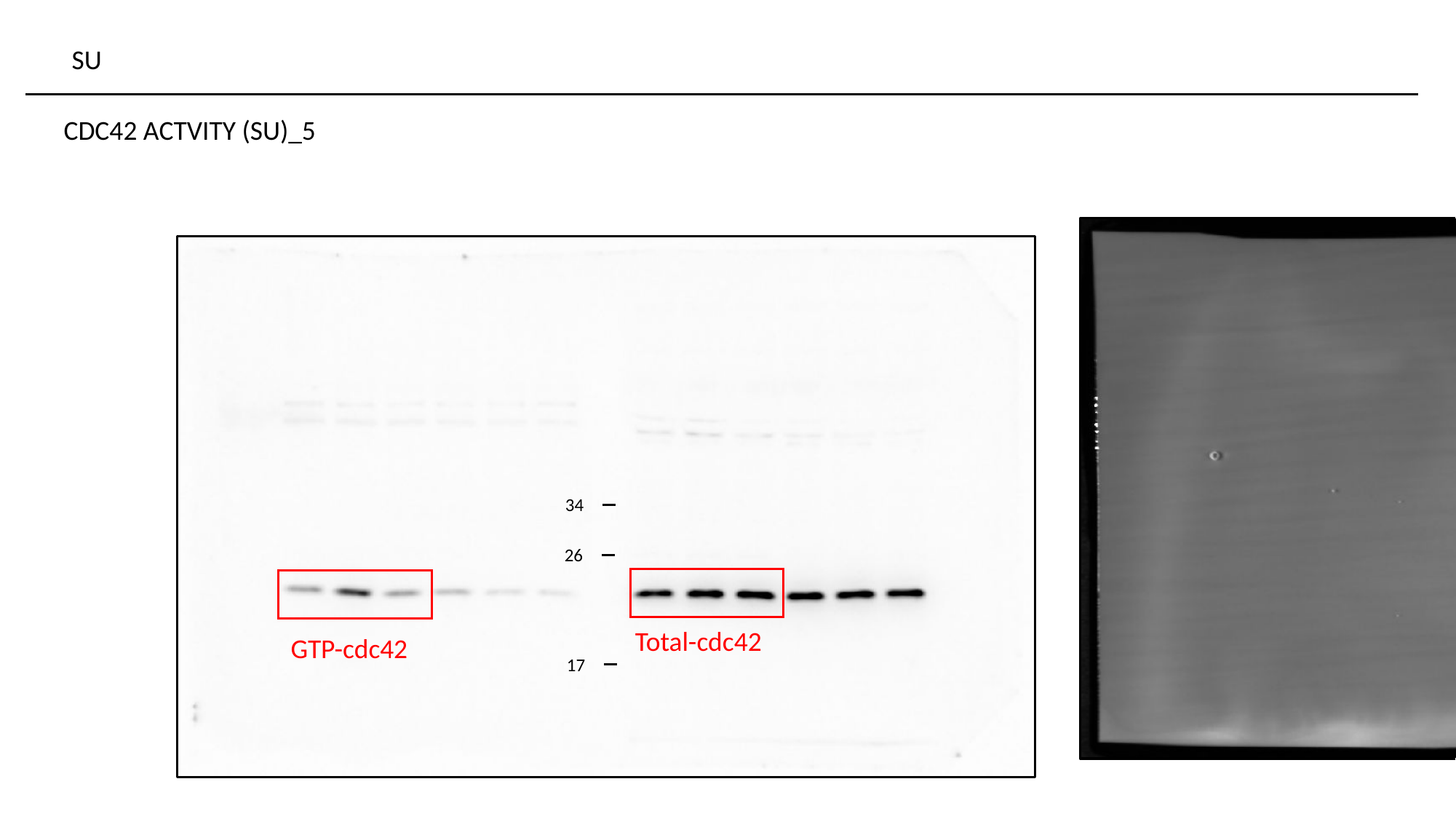

SU
CDC42 ACTVITY (SU)_5
34
26
Total-cdc42
GTP-cdc42
17

## Slide 14
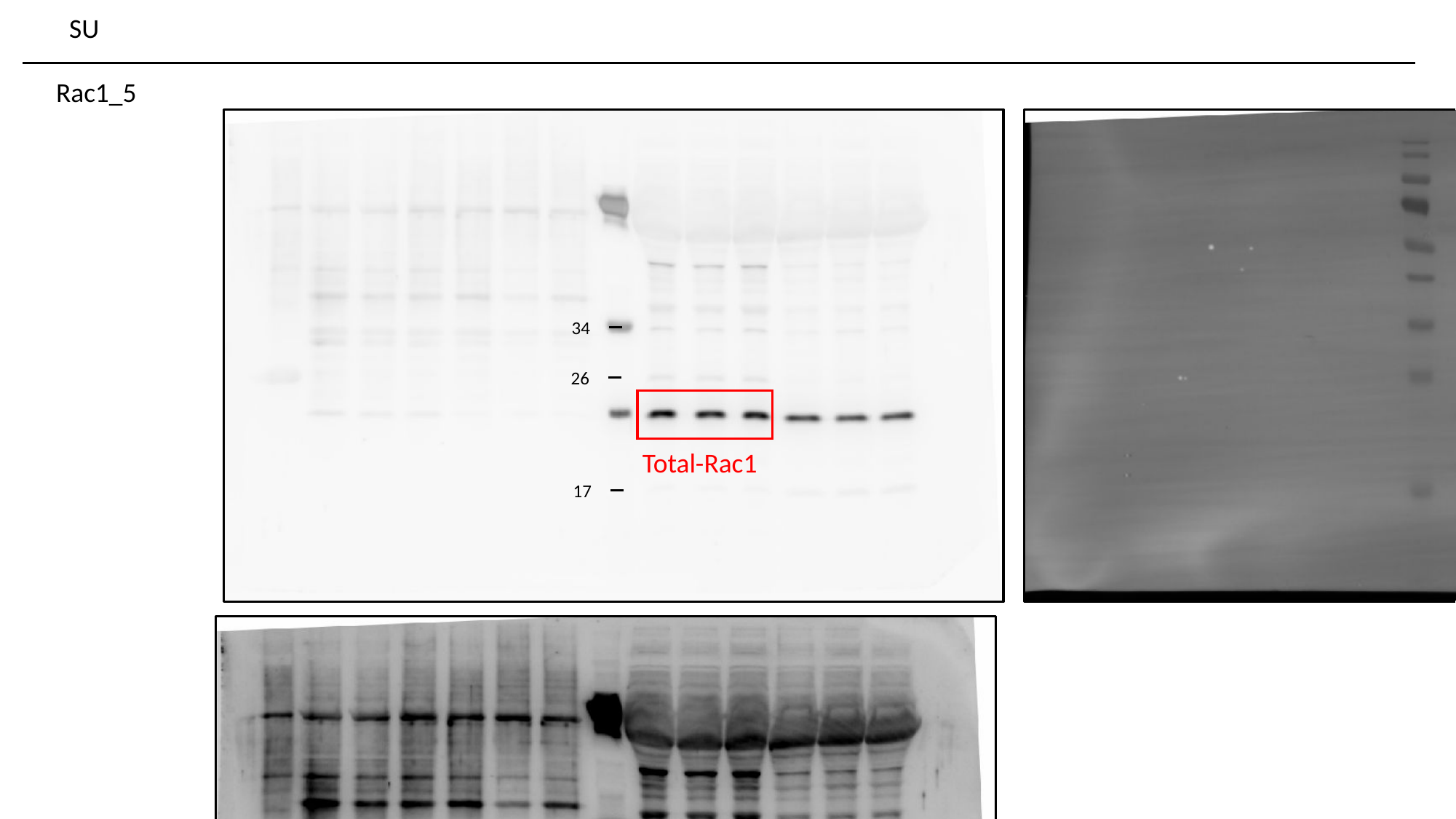

SU
Rac1_5
34
26
Total-Rac1
17
34
26
GTP-Rac1
17

## Slide 15
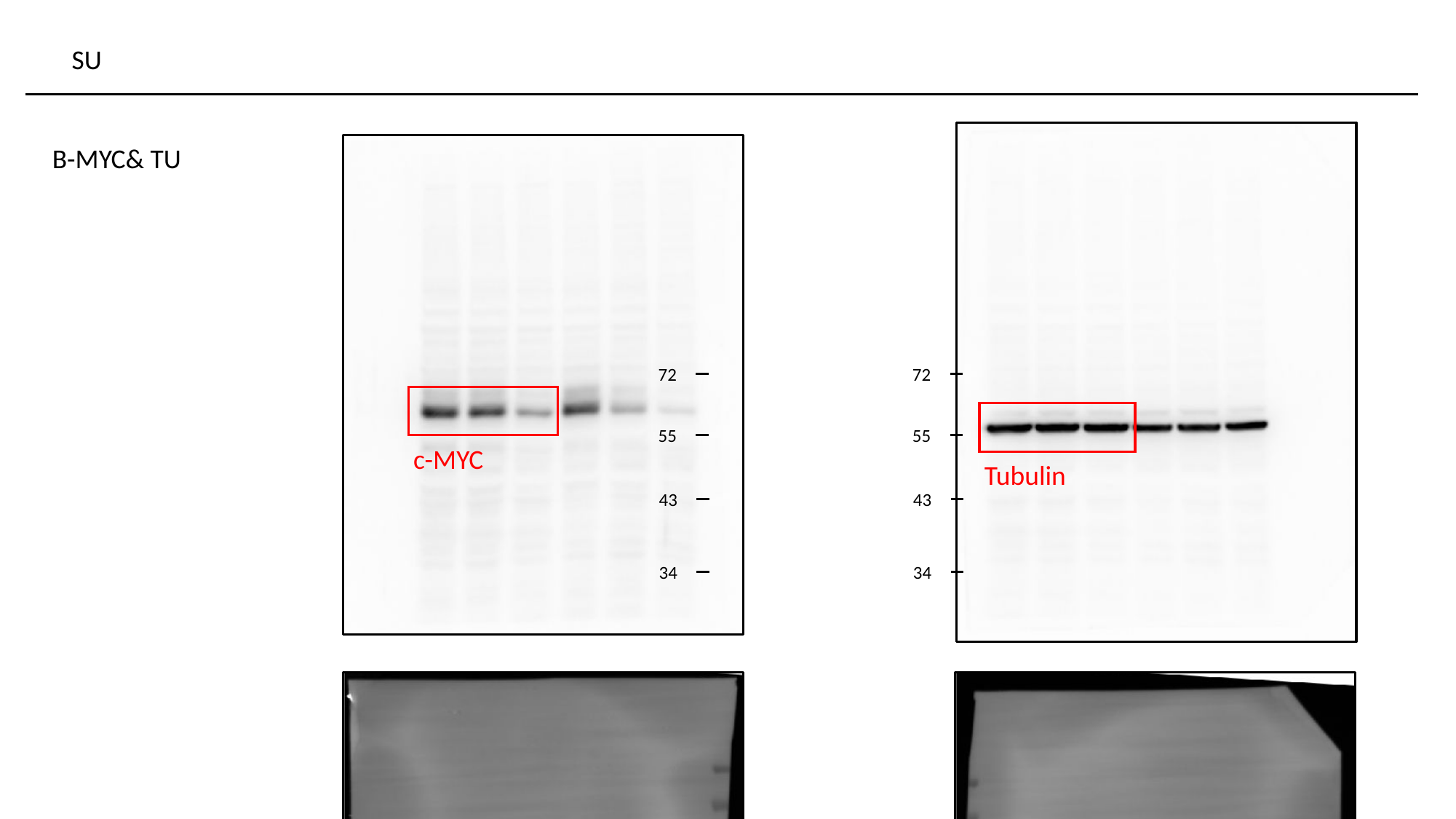

SU
B-MYC& TU
72
72
55
55
c-MYC
Tubulin
43
43
34
34

## Slide 16
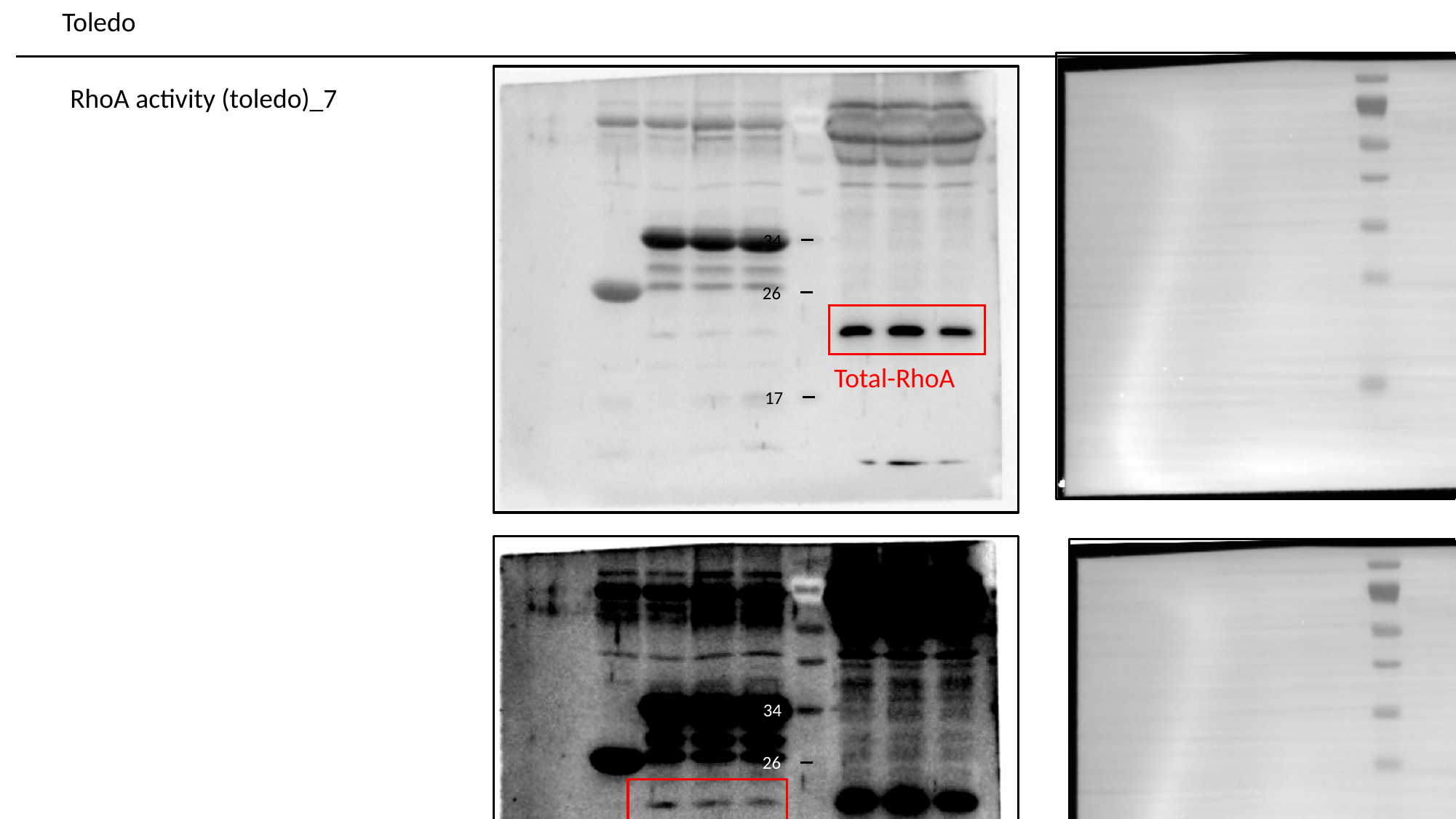

Toledo
RhoA activity (toledo)_7
34
26
Total-RhoA
17
34
26
GTP-RhoA
17

## Slide 17
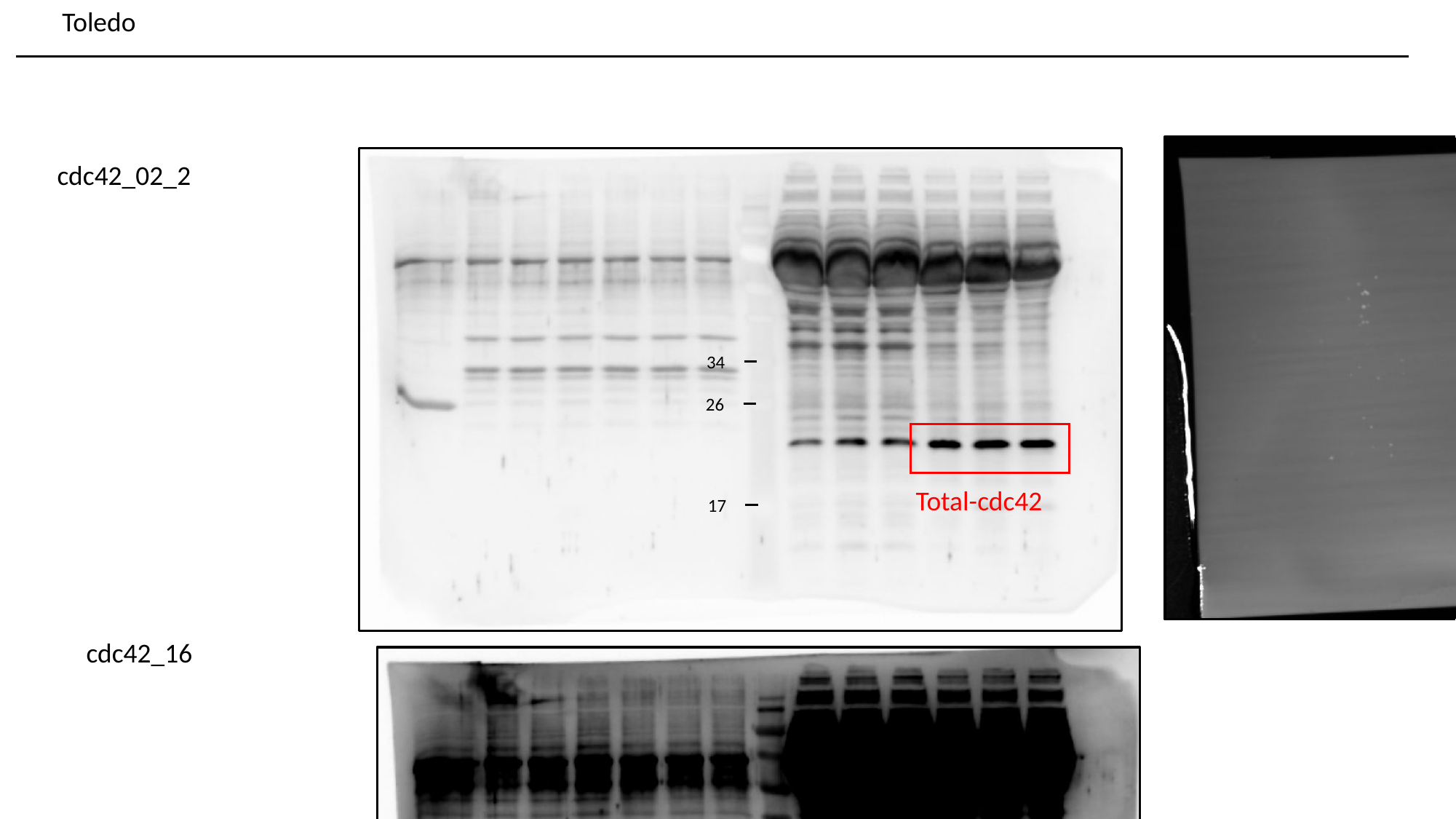

Toledo
cdc42_02_2
34
26
Total-cdc42
17
cdc42_16
34
26
GTP-cdc42
17

## Slide 18
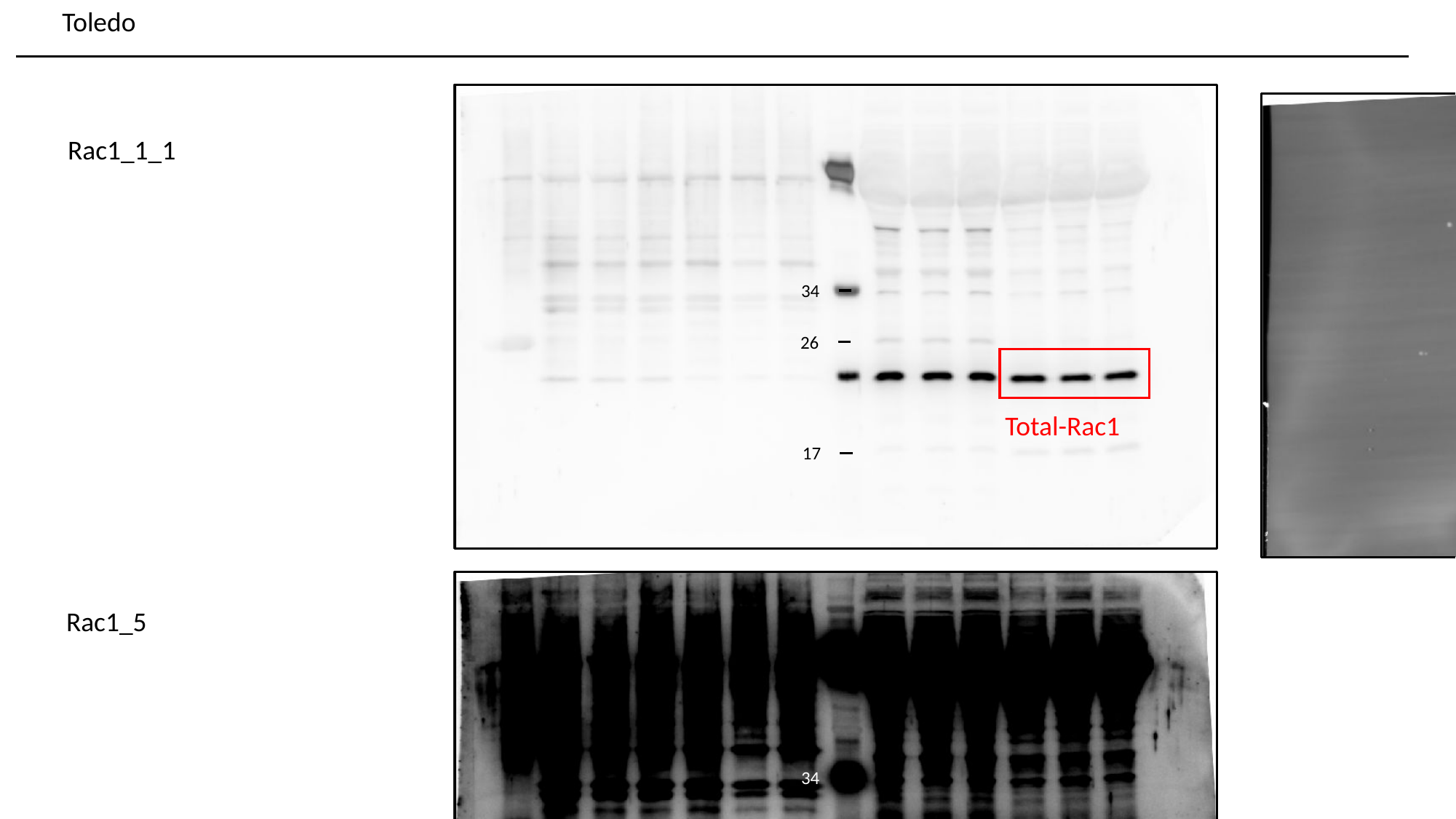

Toledo
Rac1_1_1
34
26
Total-Rac1
17
Rac1_5
34
26
GTP-Rac1
17

## Slide 19
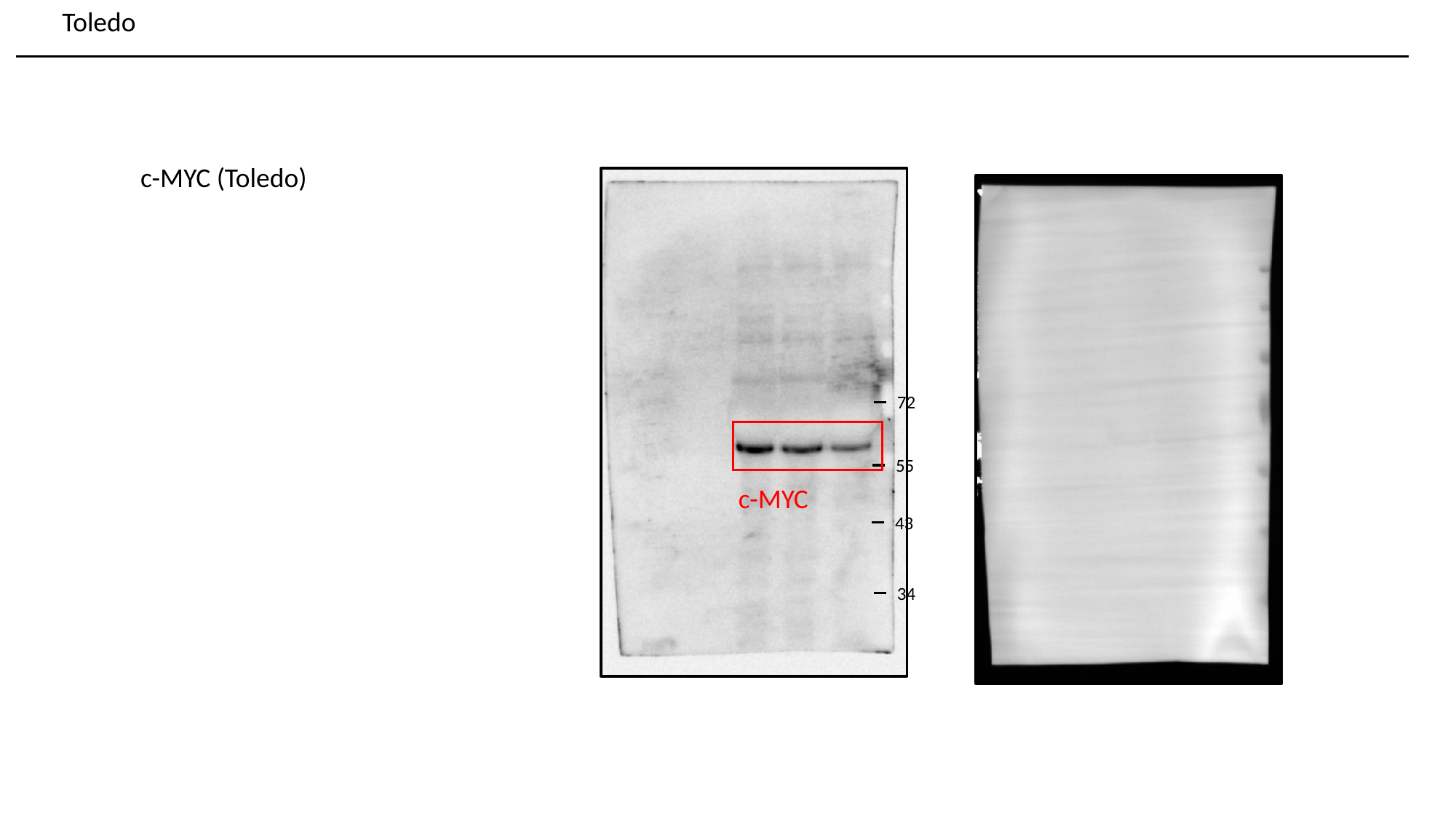

Toledo
c-MYC (Toledo)
72
55
c-MYC
43
34

## Slide 20
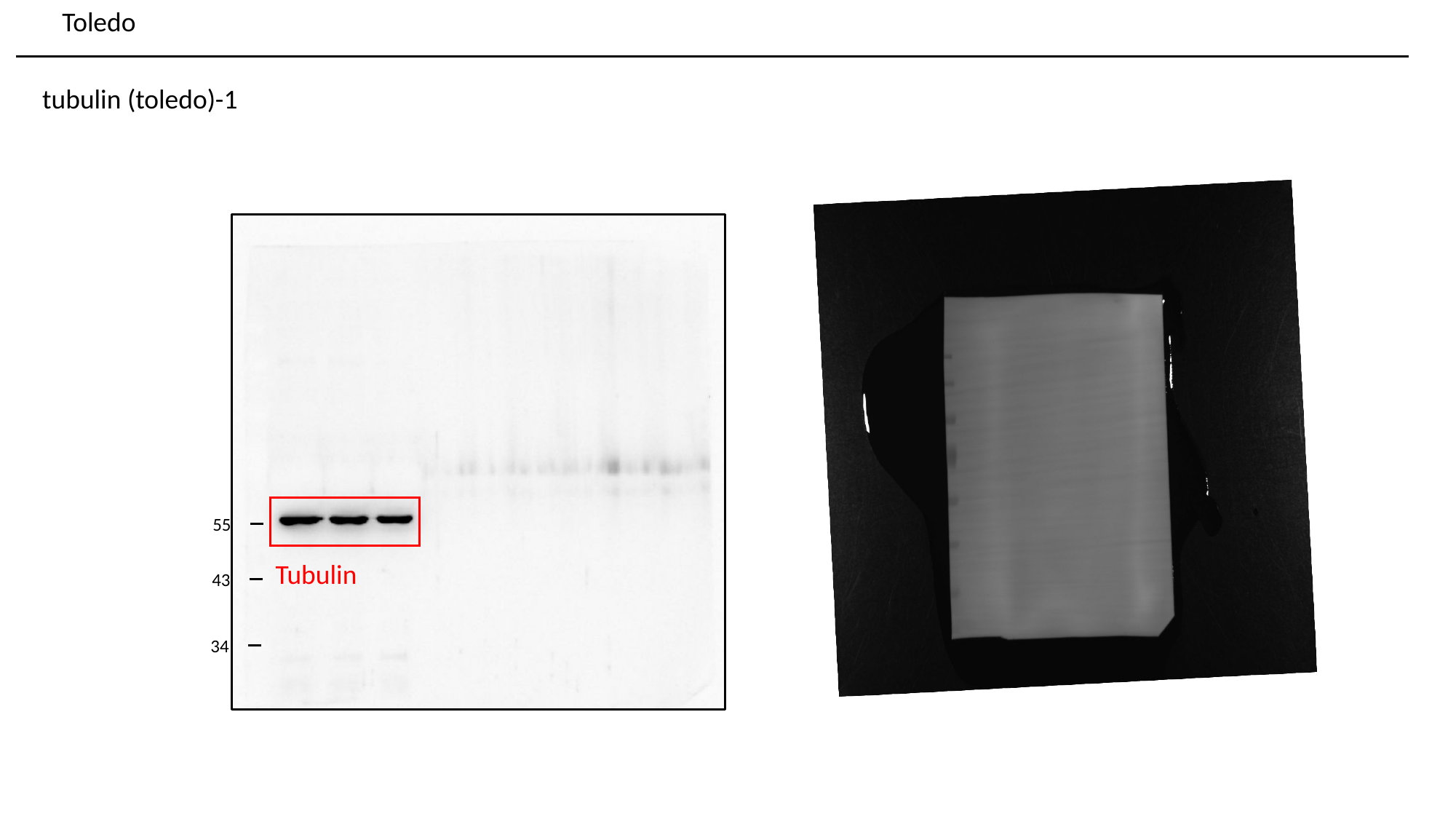

Toledo
tubulin (toledo)-1
55
Tubulin
43
34

## Slide 21
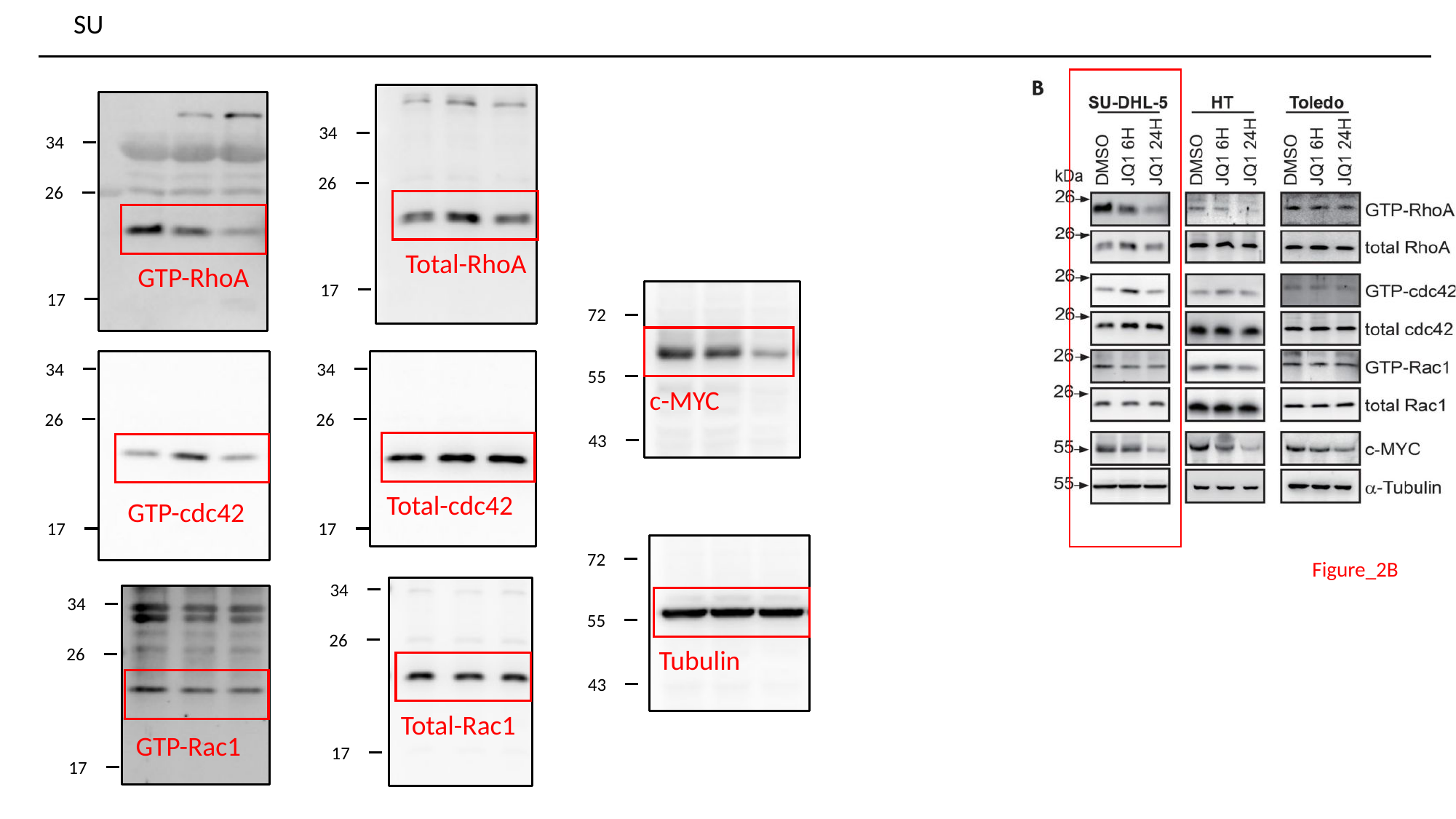

SU
34
34
26
26
Total-RhoA
GTP-RhoA
17
17
72
34
34
55
c-MYC
26
26
43
Total-cdc42
GTP-cdc42
17
17
72
Figure_2B
34
34
55
26
26
Tubulin
43
Total-Rac1
GTP-Rac1
17
17

## Slide 22
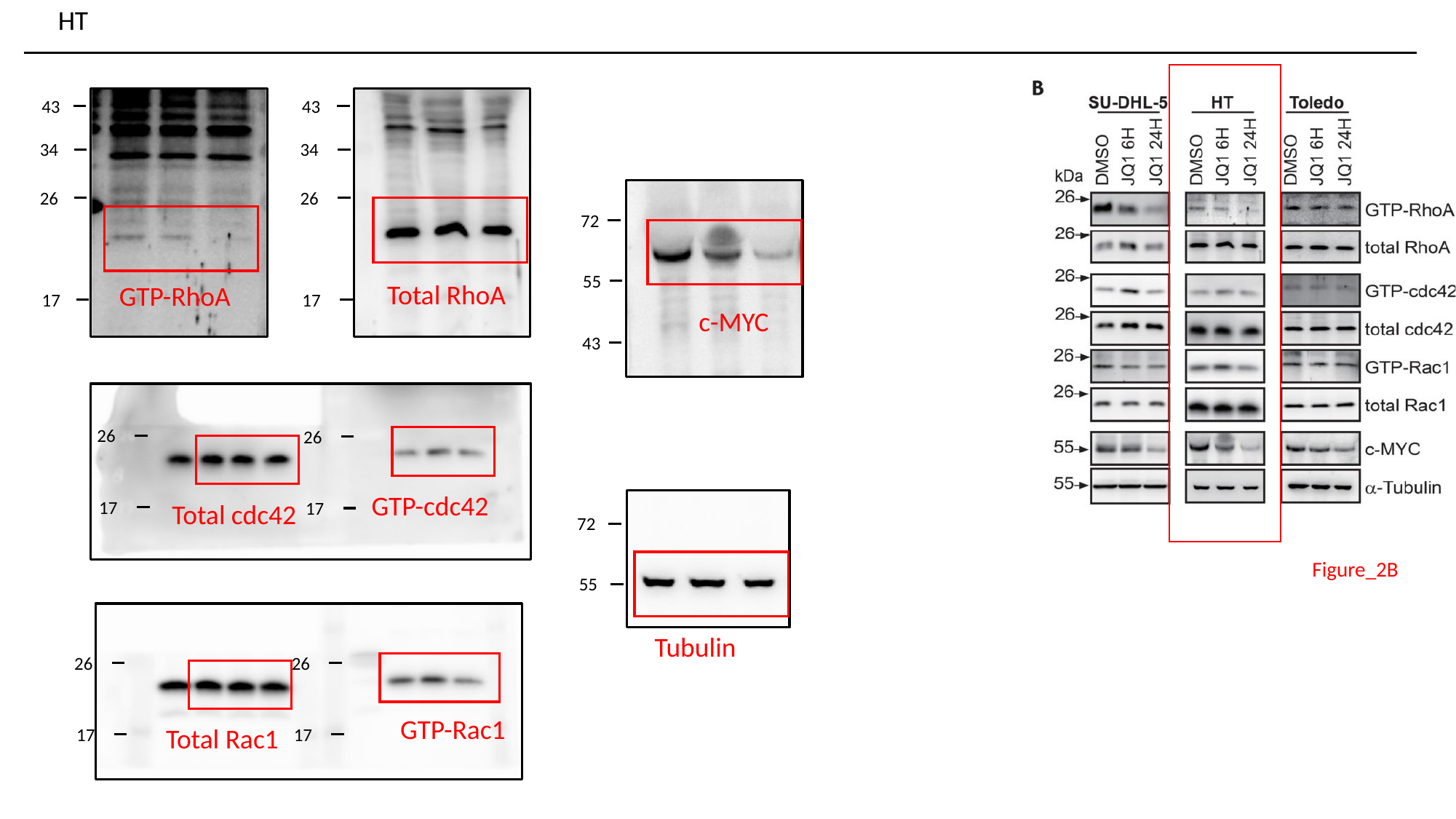

HT
43
43
34
34
26
26
72
55
Total RhoA
GTP-RhoA
17
17
c-MYC
43
26
26
GTP-cdc42
17
17
Total cdc42
72
Figure_2B
55
Tubulin
26
26
GTP-Rac1
Total Rac1
17
17

## Slide 23
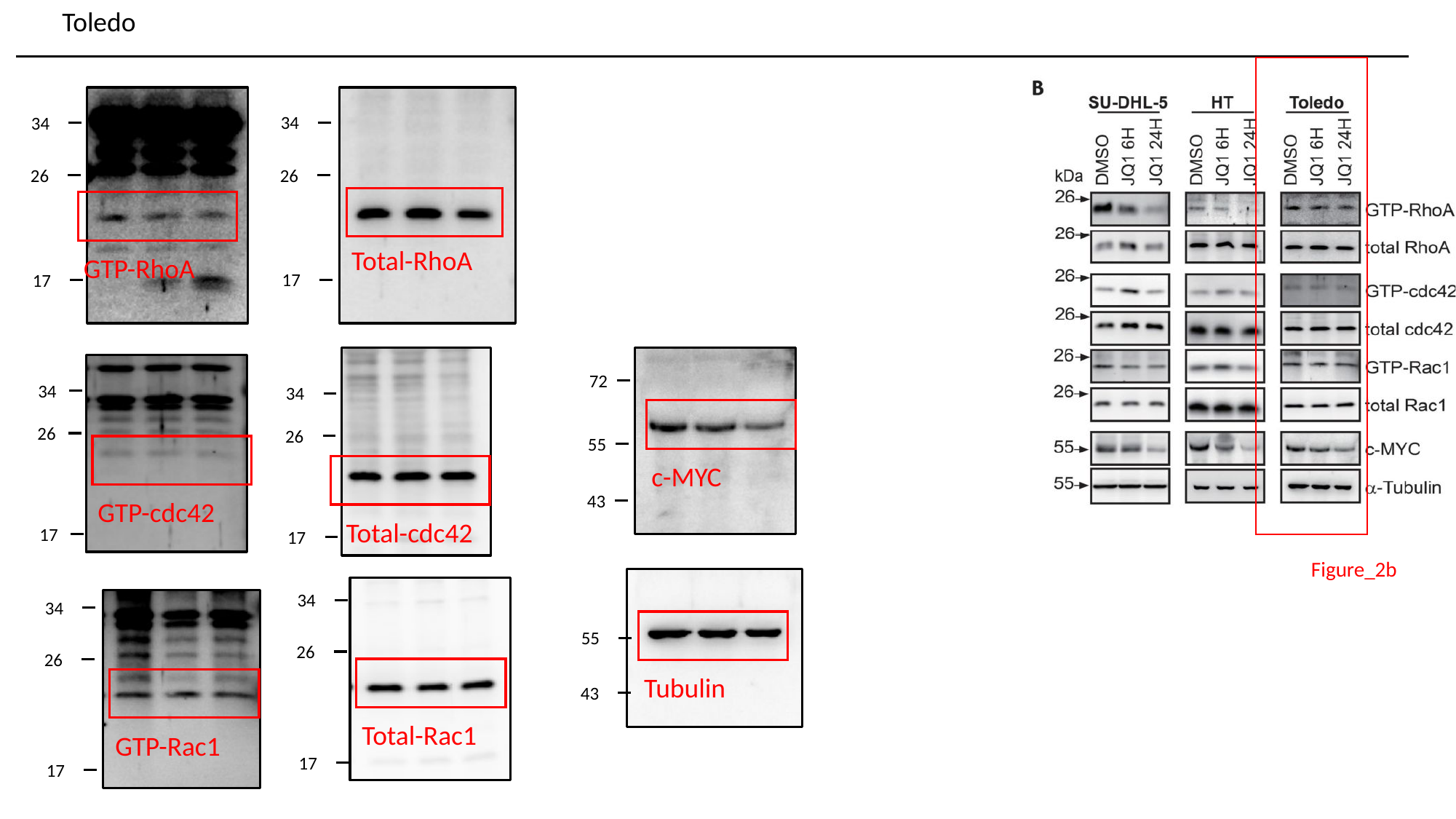

Toledo
34
34
26
26
Total-RhoA
GTP-RhoA
17
17
72
34
34
26
26
55
c-MYC
43
GTP-cdc42
Total-cdc42
17
17
Figure_2b
34
34
55
26
26
Tubulin
43
Total-Rac1
GTP-Rac1
17
17

## Slide 24
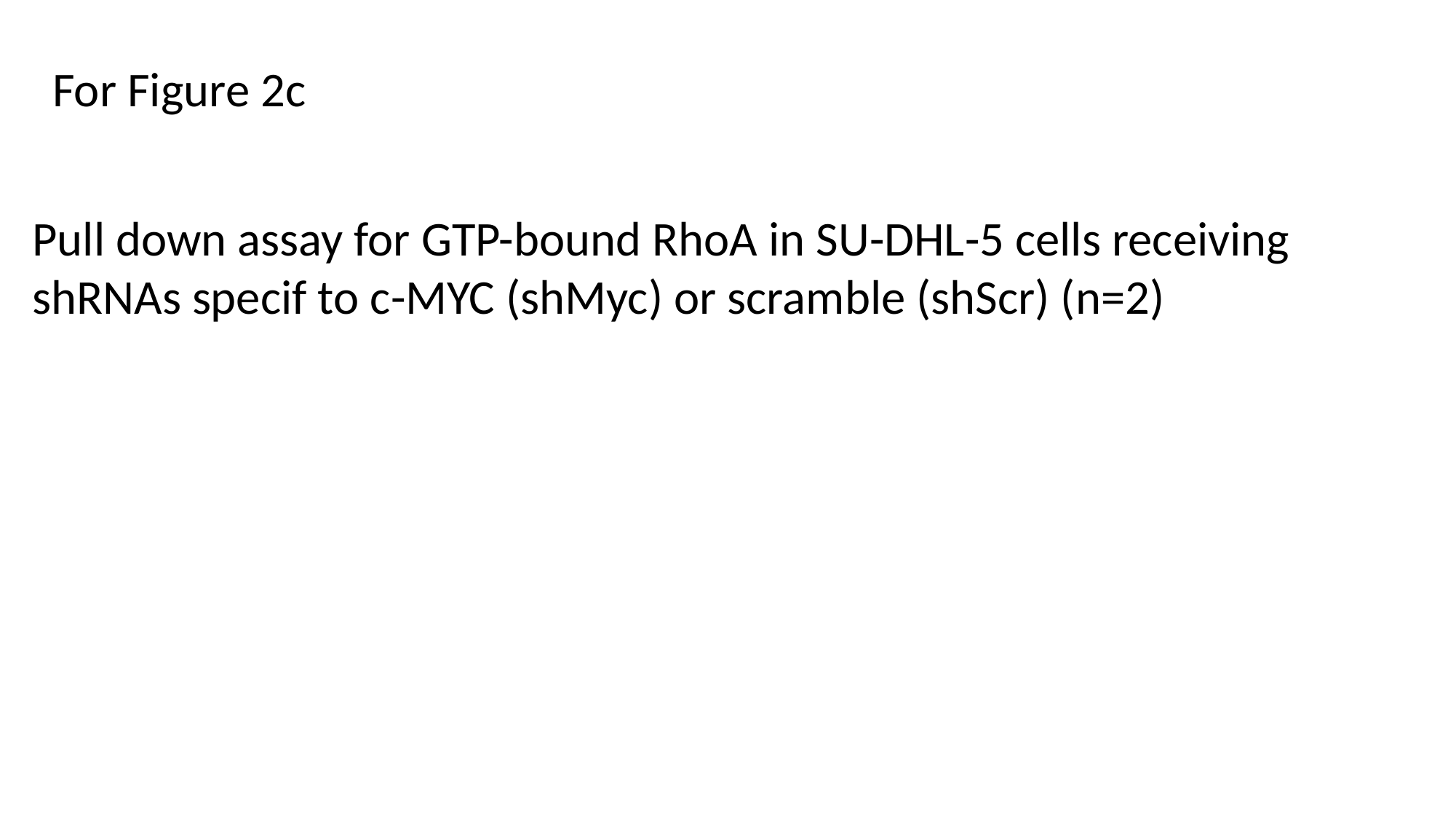

For Figure 2c
Pull down assay for GTP-bound RhoA in SU-DHL-5 cells receiving shRNAs specif to c-MYC (shMyc) or scramble (shScr) (n=2)

## Slide 25
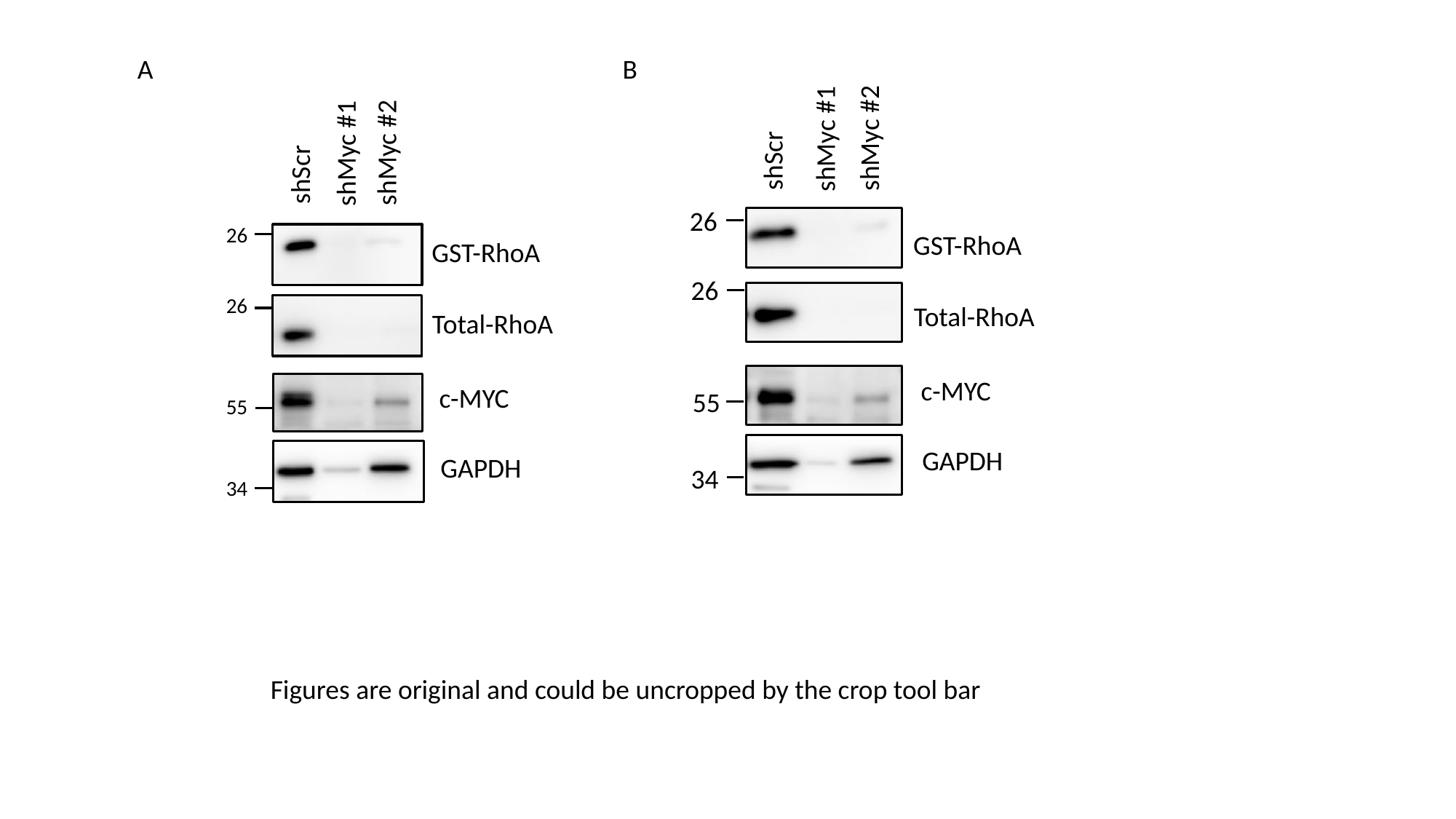

A
B
shMyc #2
shMyc #1
shMyc #2
shMyc #1
shScr
shScr
26
26
GST-RhoA
GST-RhoA
26
26
Total-RhoA
Total-RhoA
c-MYC
c-MYC
55
55
GAPDH
GAPDH
34
34
Figures are original and could be uncropped by the crop tool bar

## Slide 26
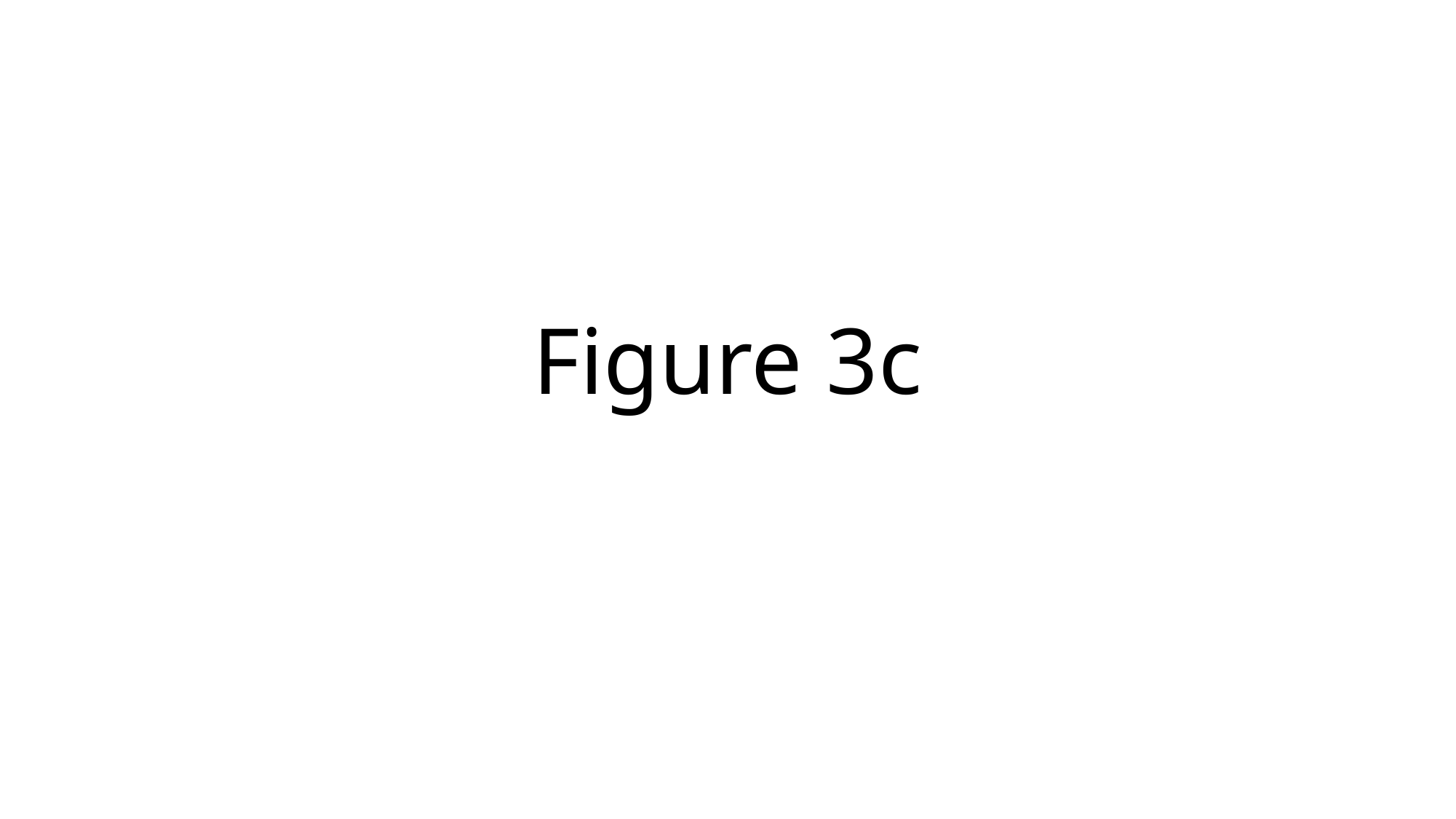

# Figure 3c

## Slide 27
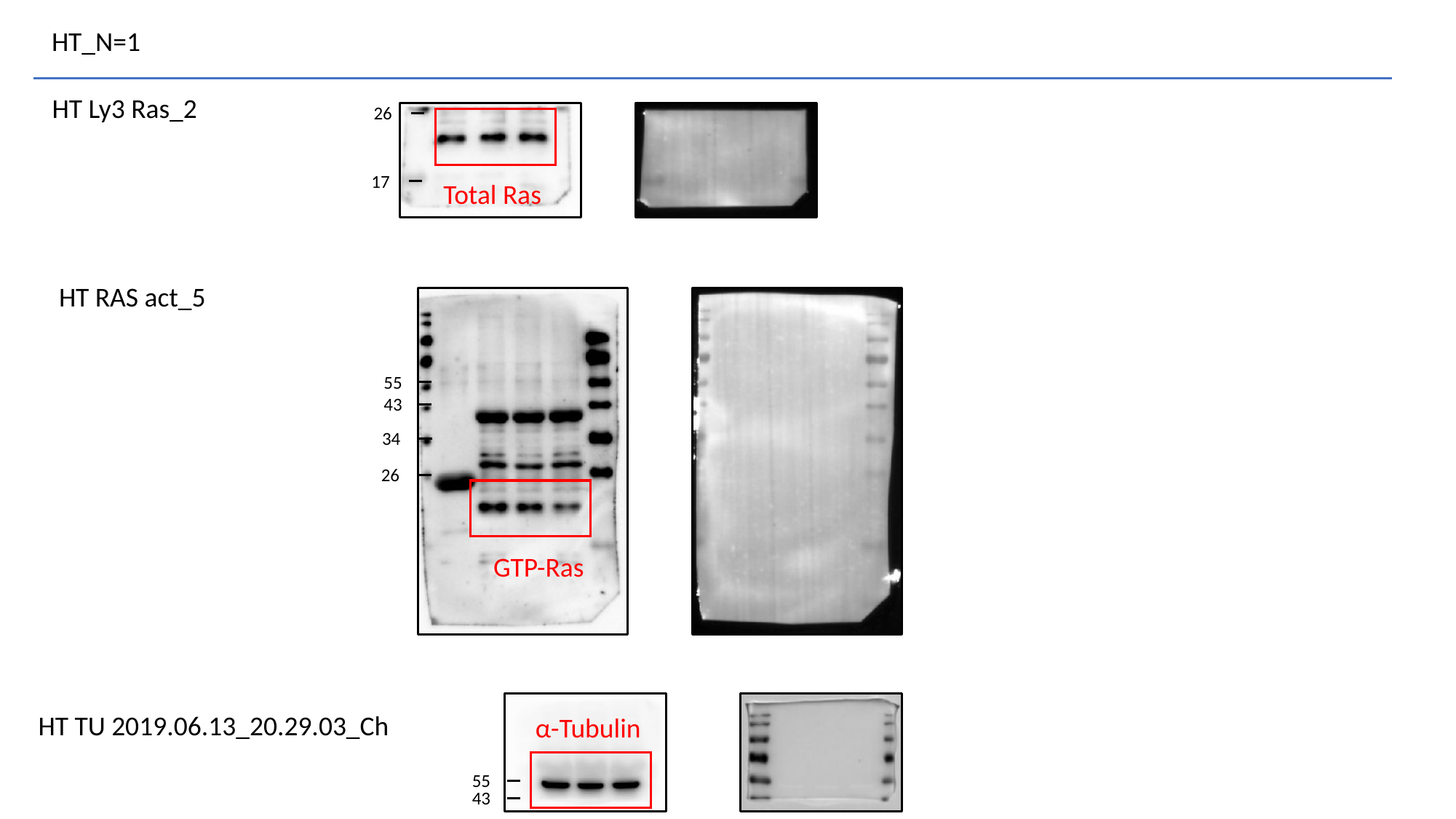

HT_N=1
HT Ly3 Ras_2
26
17
Total Ras
HT RAS act_5
55
43
34
26
GTP-Ras
HT TU 2019.06.13_20.29.03_Ch
α-Tubulin
55
43

## Slide 28
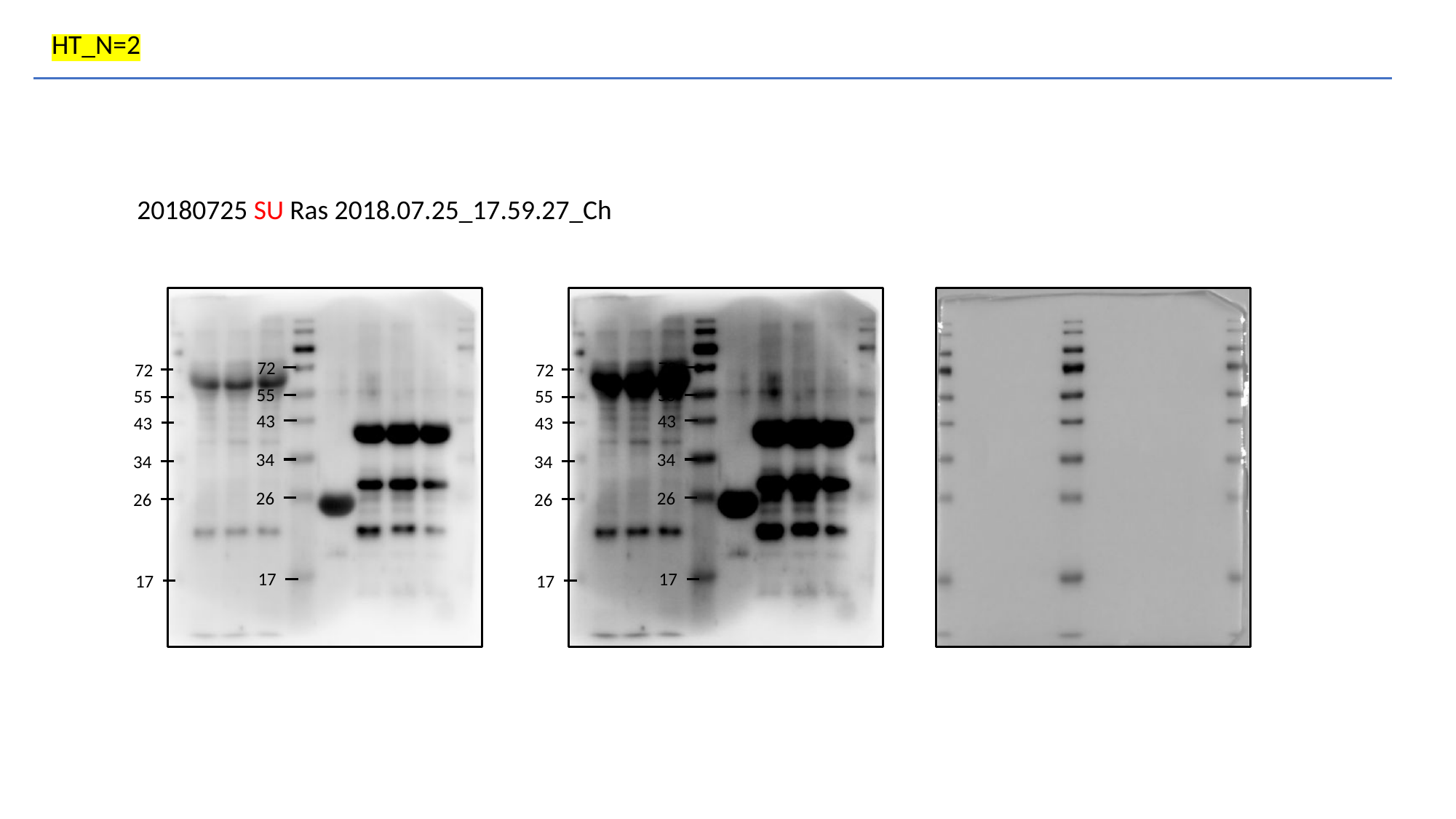

HT_N=2
20180725 SU Ras 2018.07.25_17.59.27_Ch
72
72
72
72
55
55
55
55
43
43
43
43
34
34
34
34
26
26
26
26
17
17
17
17

## Slide 29
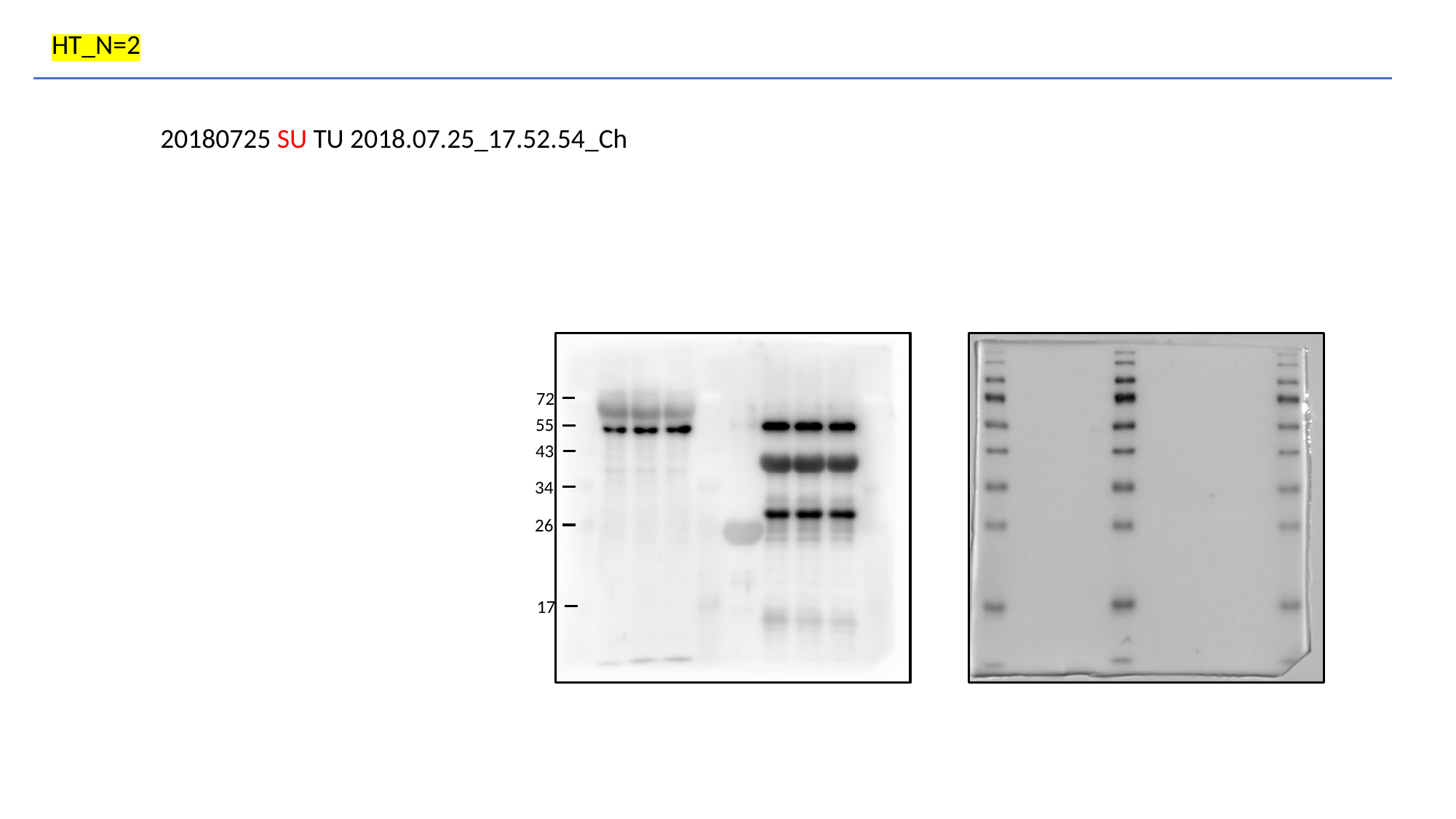

HT_N=2
20180725 SU TU 2018.07.25_17.52.54_Ch
72
55
43
34
26
17

## Slide 30
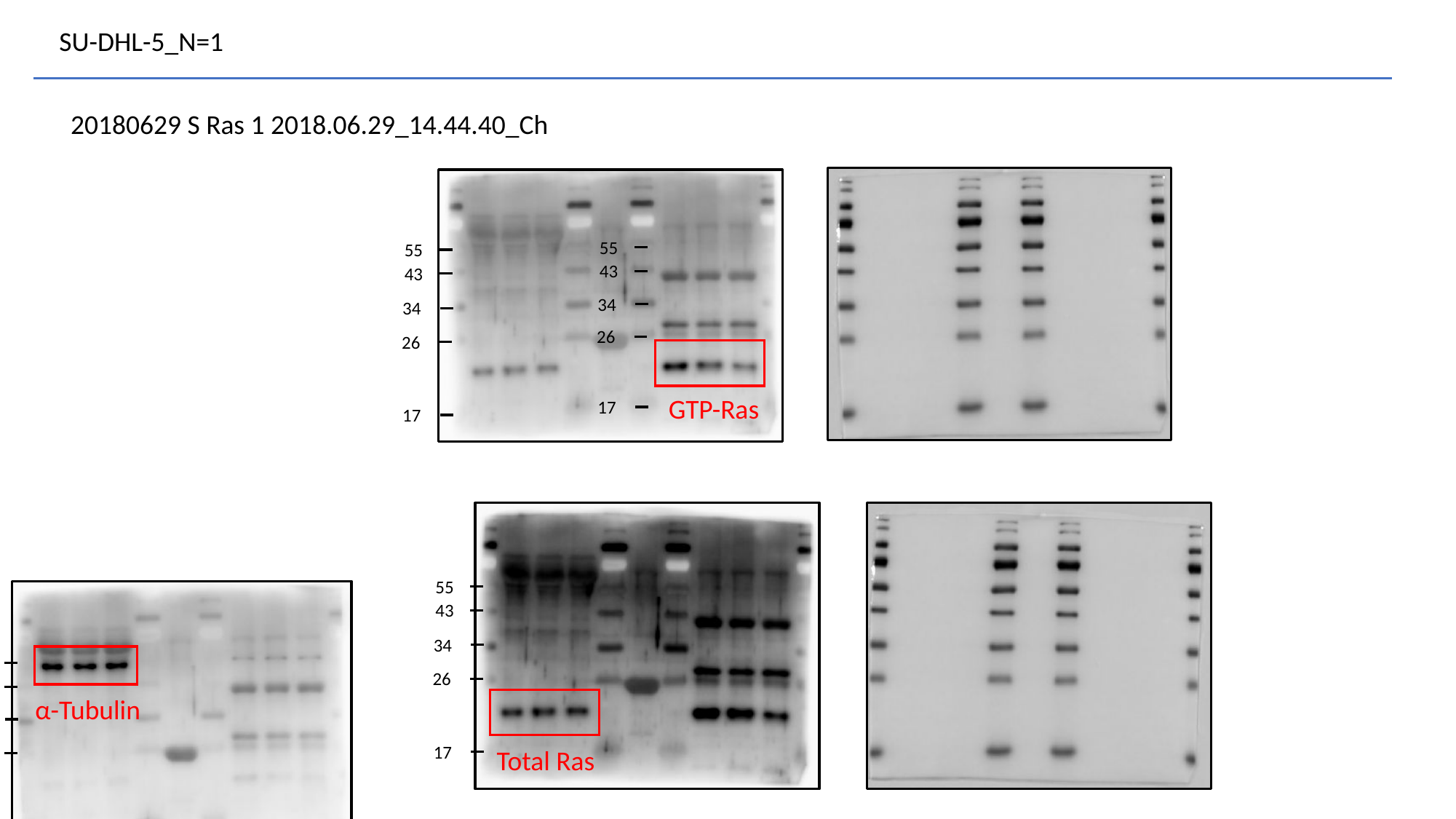

SU-DHL-5_N=1
20180629 S Ras 1 2018.06.29_14.44.40_Ch
55
55
43
43
34
34
26
26
GTP-Ras
17
17
55
43
34
55
26
43
α-Tubulin
34
17
26
Total Ras
17

## Slide 31
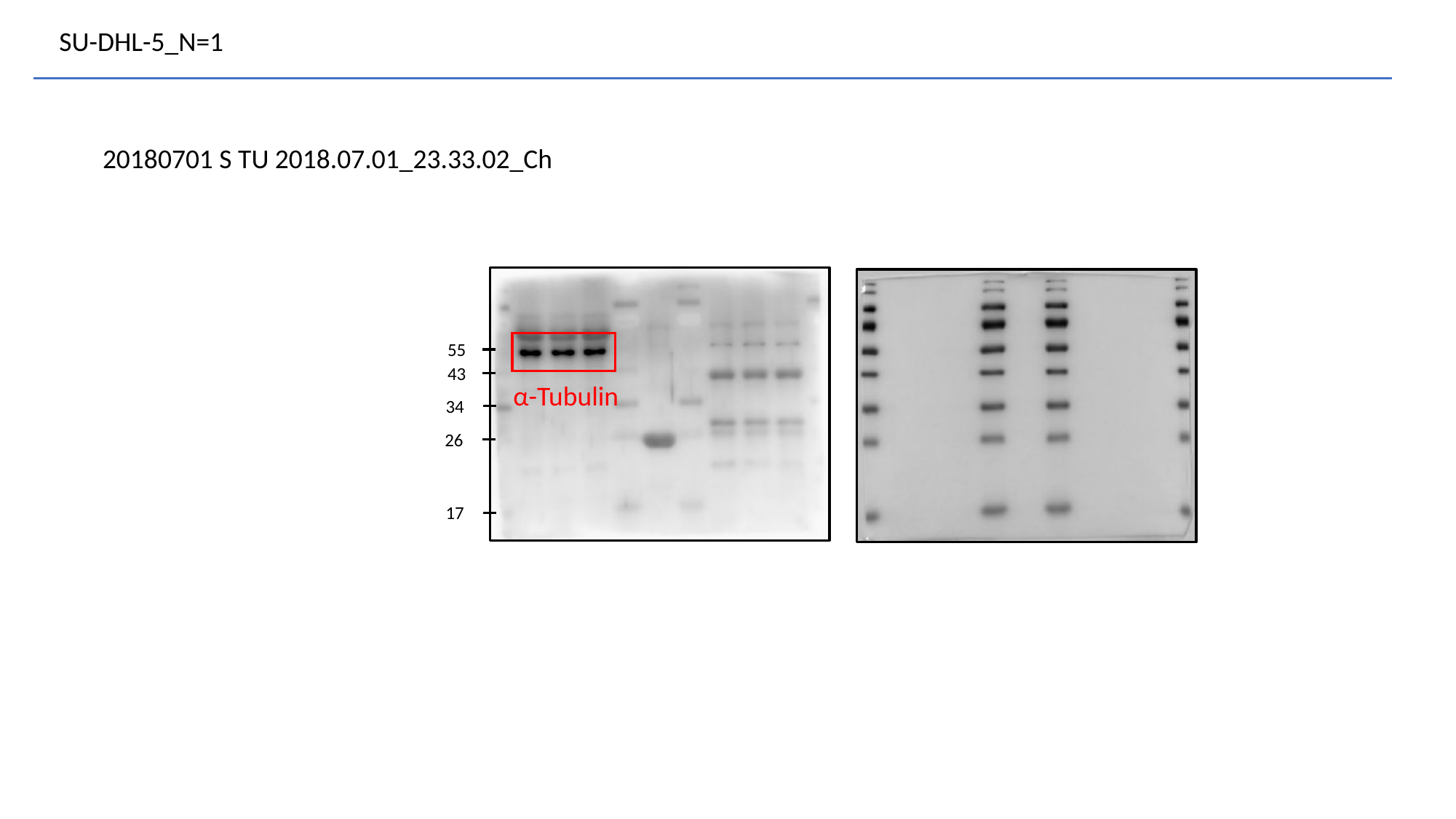

SU-DHL-5_N=1
20180701 S TU 2018.07.01_23.33.02_Ch
55
43
α-Tubulin
34
26
17

## Slide 32
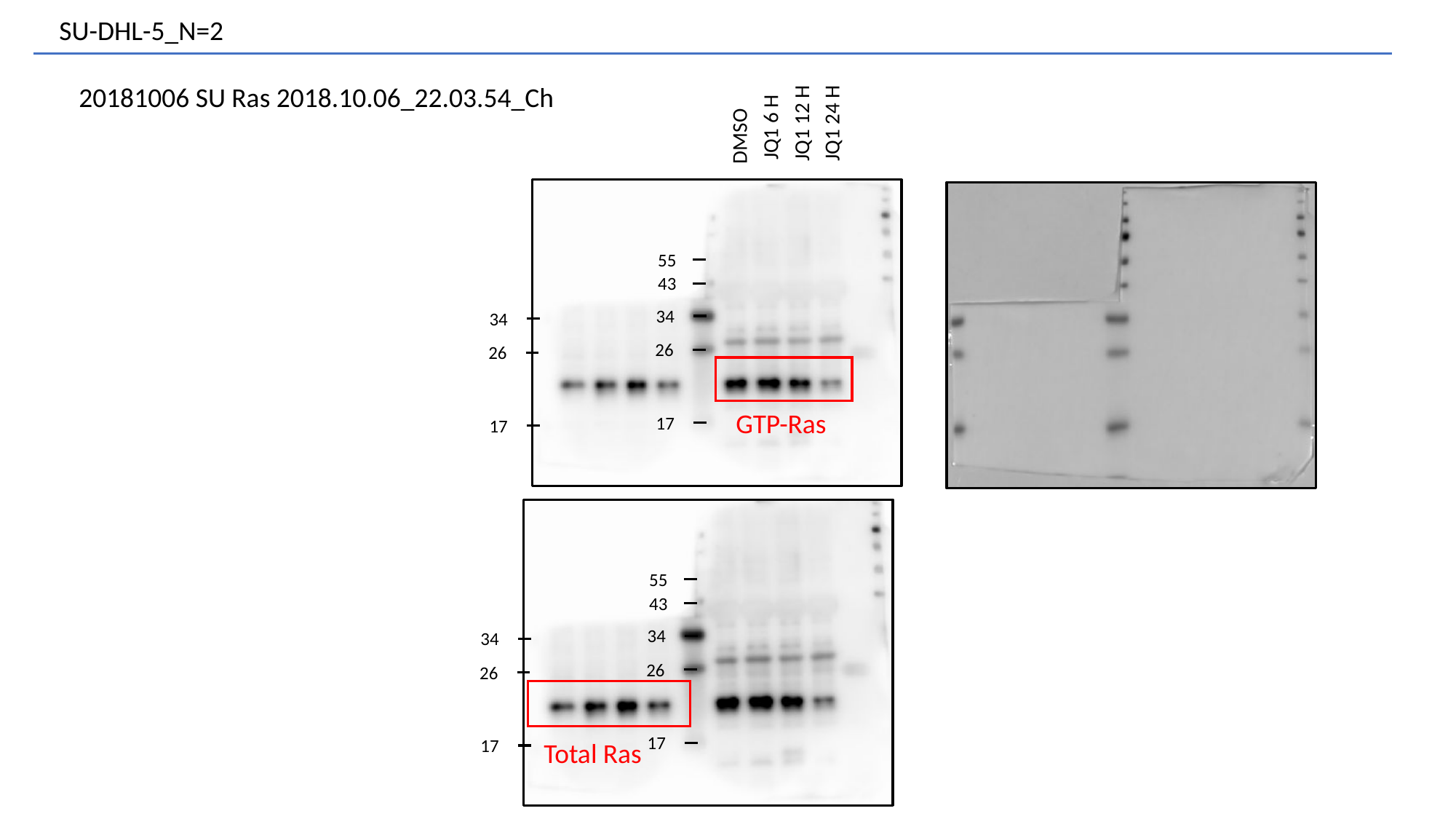

SU-DHL-5_N=2
20181006 SU Ras 2018.10.06_22.03.54_Ch
JQ1 12 H
JQ1 24 H
JQ1 6 H
DMSO
55
43
34
34
26
26
GTP-Ras
17
17
55
43
34
34
26
26
17
17
Total Ras

## Slide 33
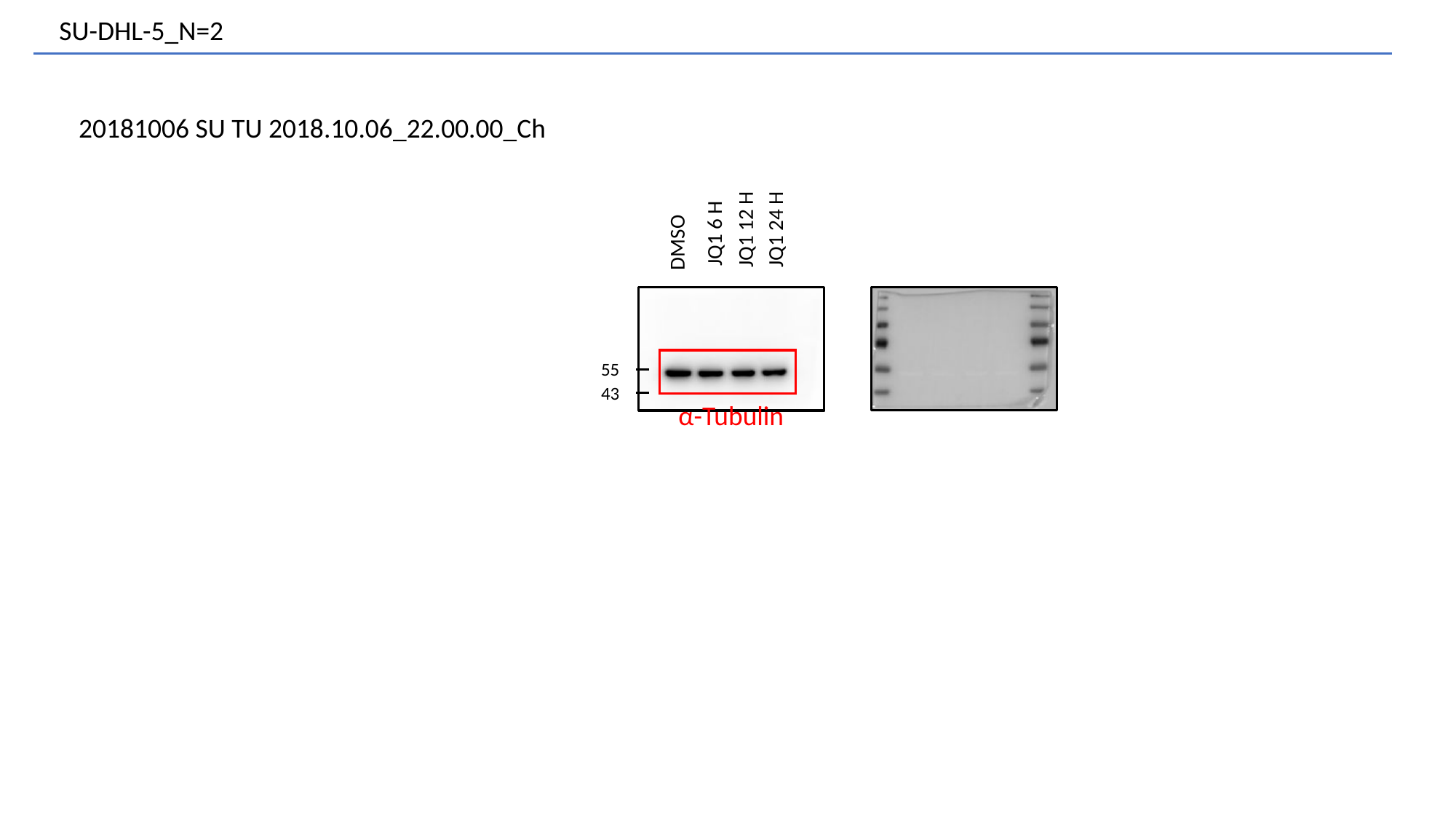

SU-DHL-5_N=2
20181006 SU TU 2018.10.06_22.00.00_Ch
JQ1 12 H
JQ1 24 H
JQ1 6 H
DMSO
55
43
α-Tubulin

## Slide 34
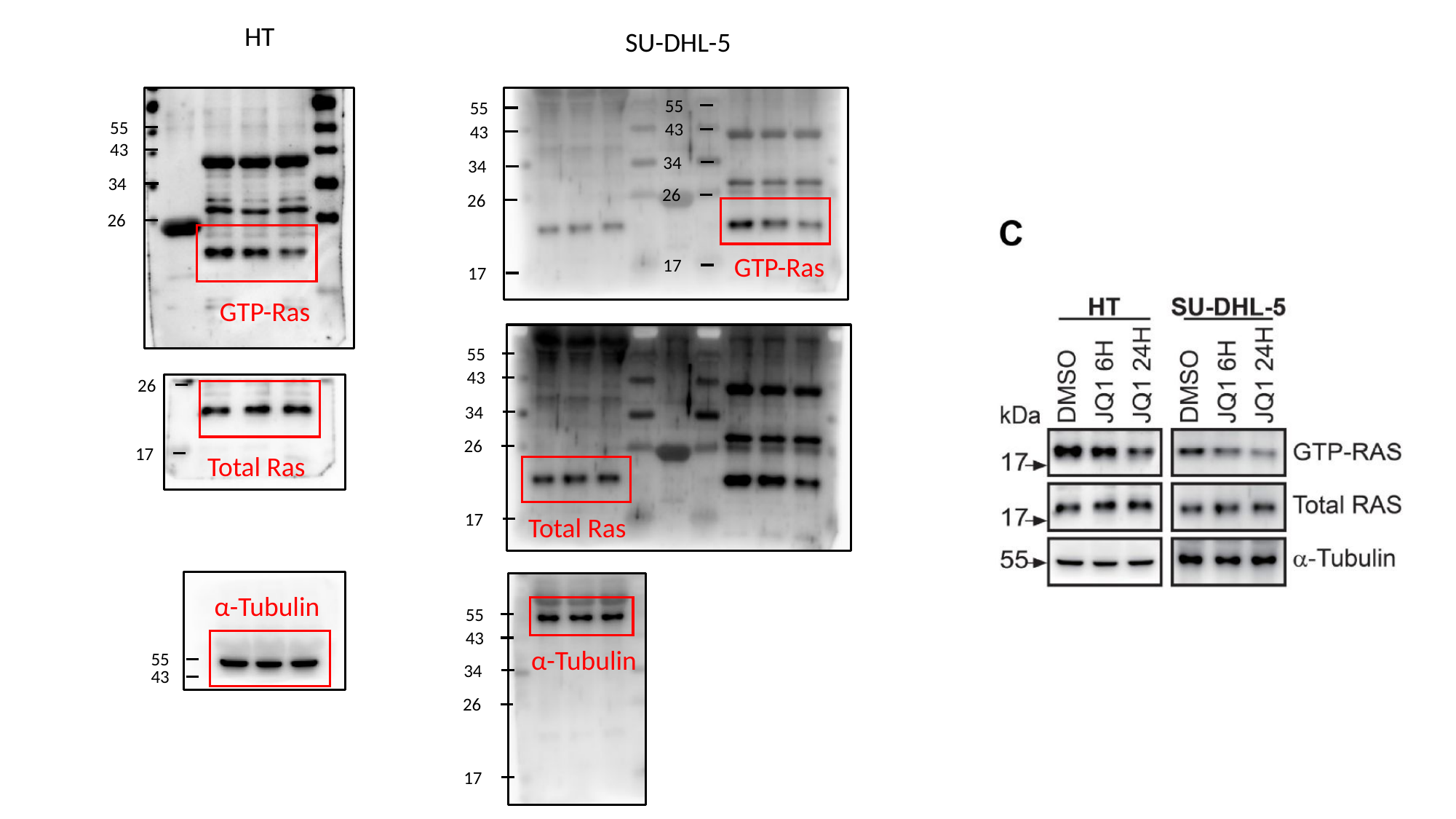

HT
SU-DHL-5
55
55
55
43
43
43
34
34
34
26
26
26
GTP-Ras
17
17
GTP-Ras
55
43
26
34
26
17
Total Ras
17
Total Ras
α-Tubulin
55
43
α-Tubulin
55
34
43
26
17

## Slide 35
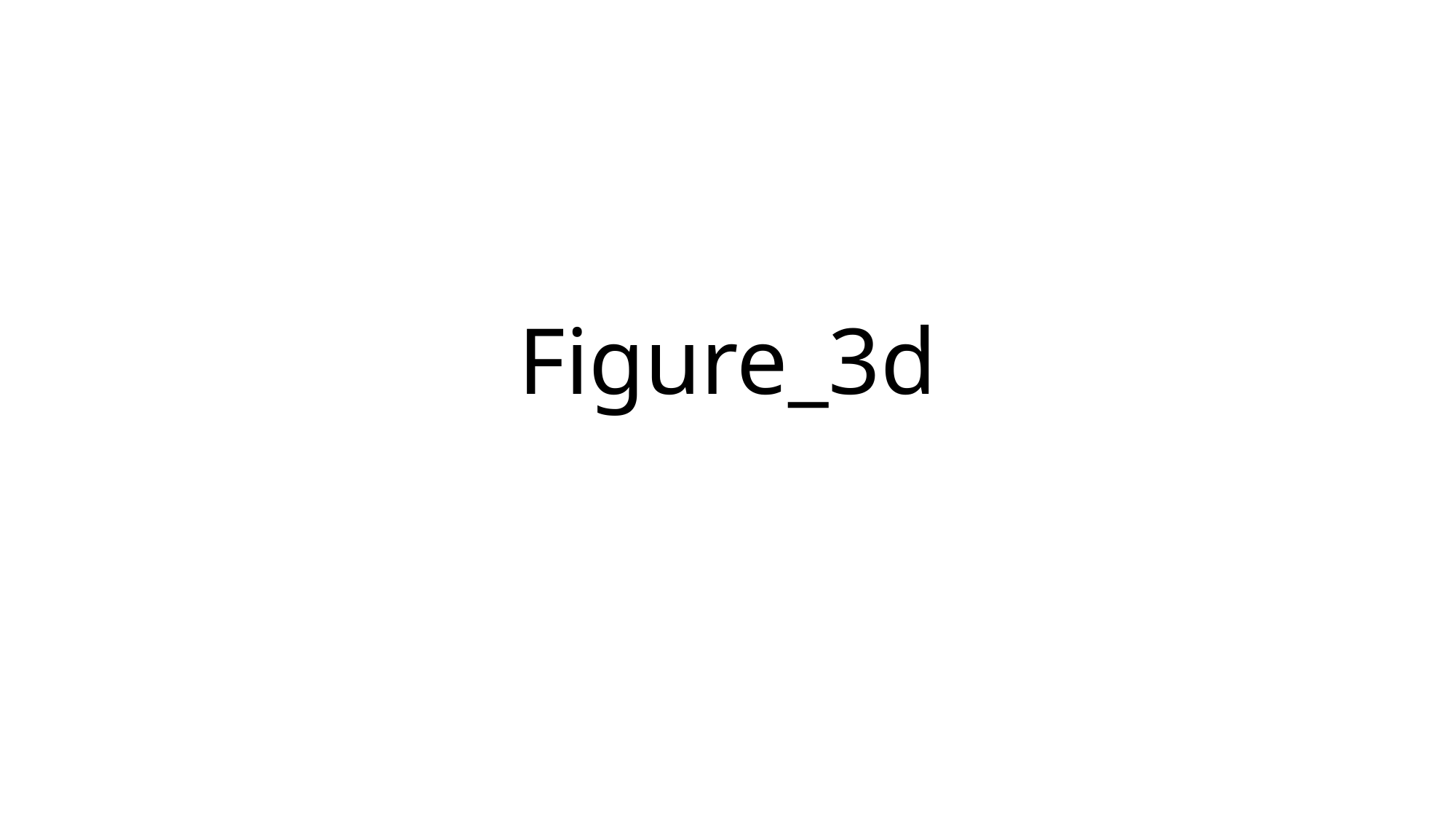

# Figure_3d

## Slide 36
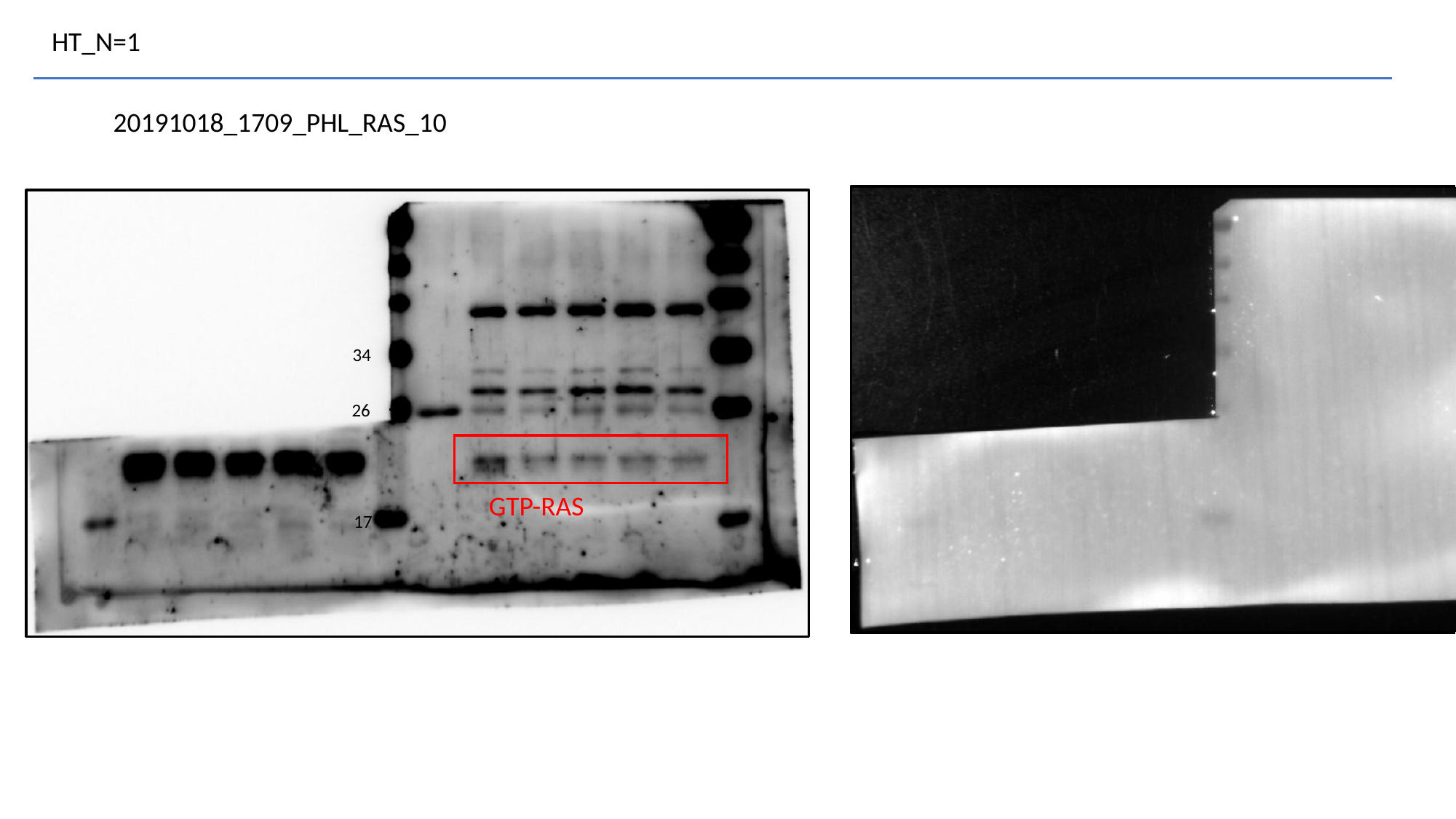

HT_N=1
20191018_1709_PHL_RAS_10
34
26
GTP-RAS
17

## Slide 37
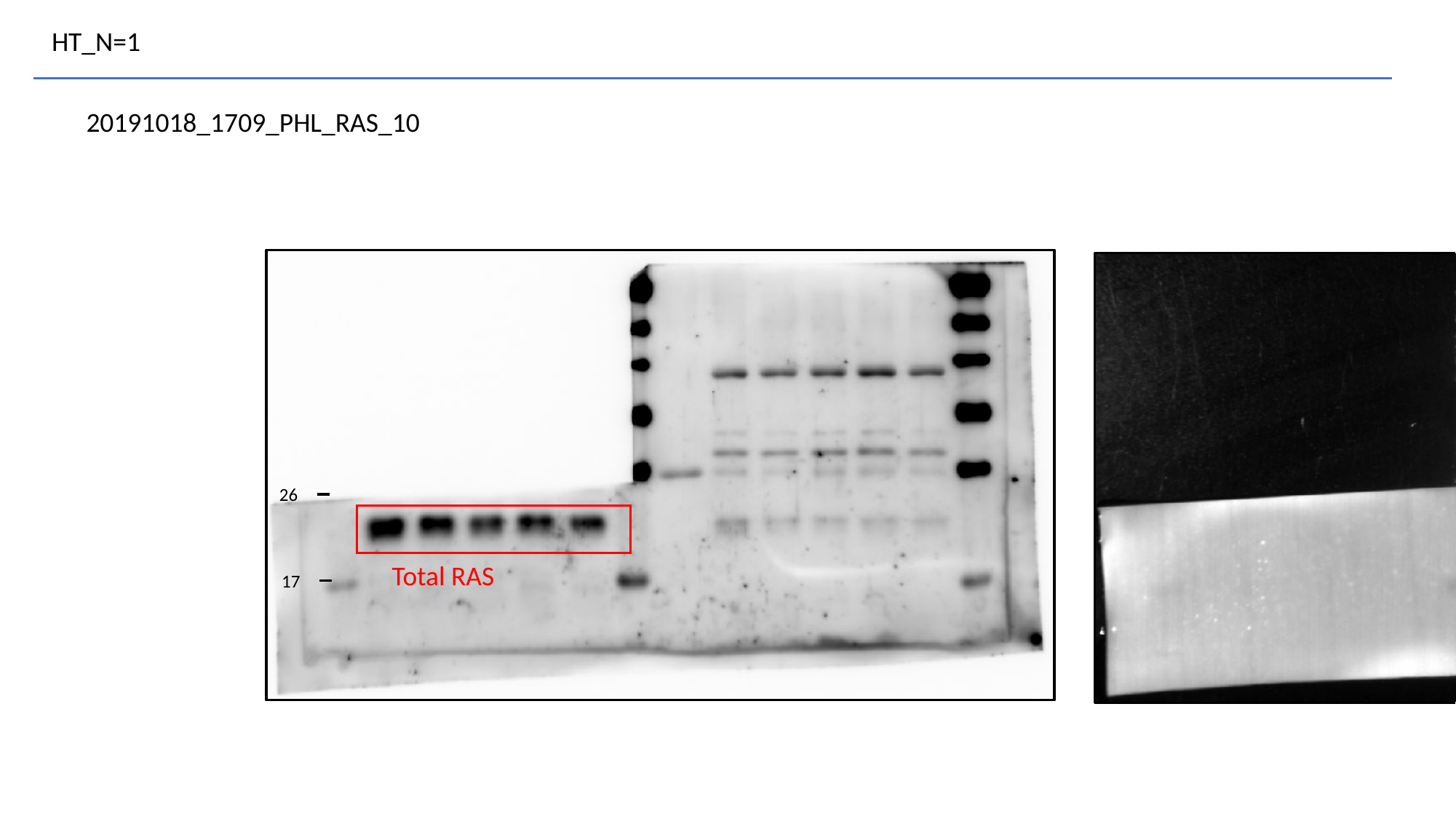

HT_N=1
20191018_1709_PHL_RAS_10
26
Total RAS
17

## Slide 38
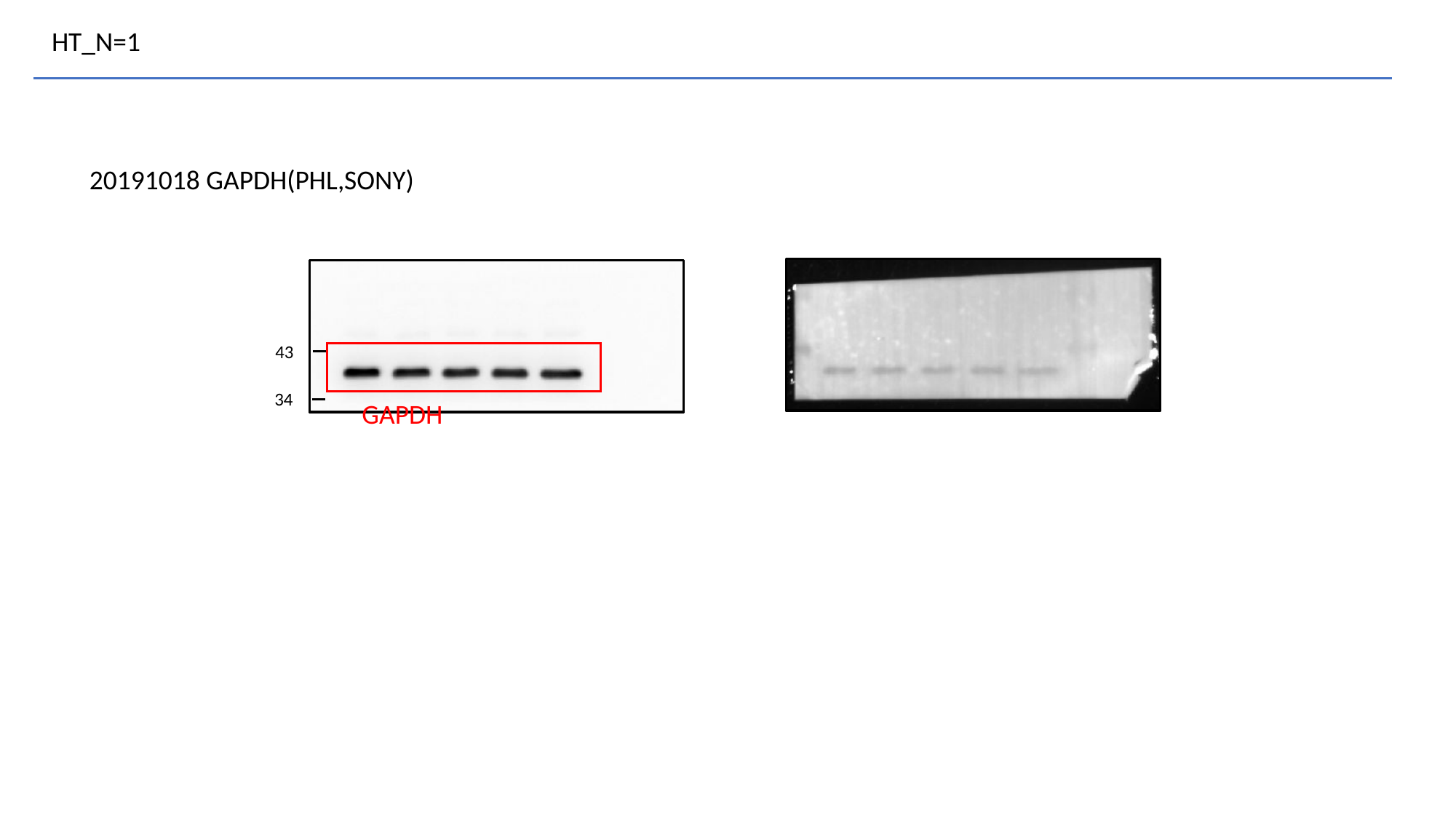

HT_N=1
20191018 GAPDH(PHL,SONY)
43
34
GAPDH

## Slide 39
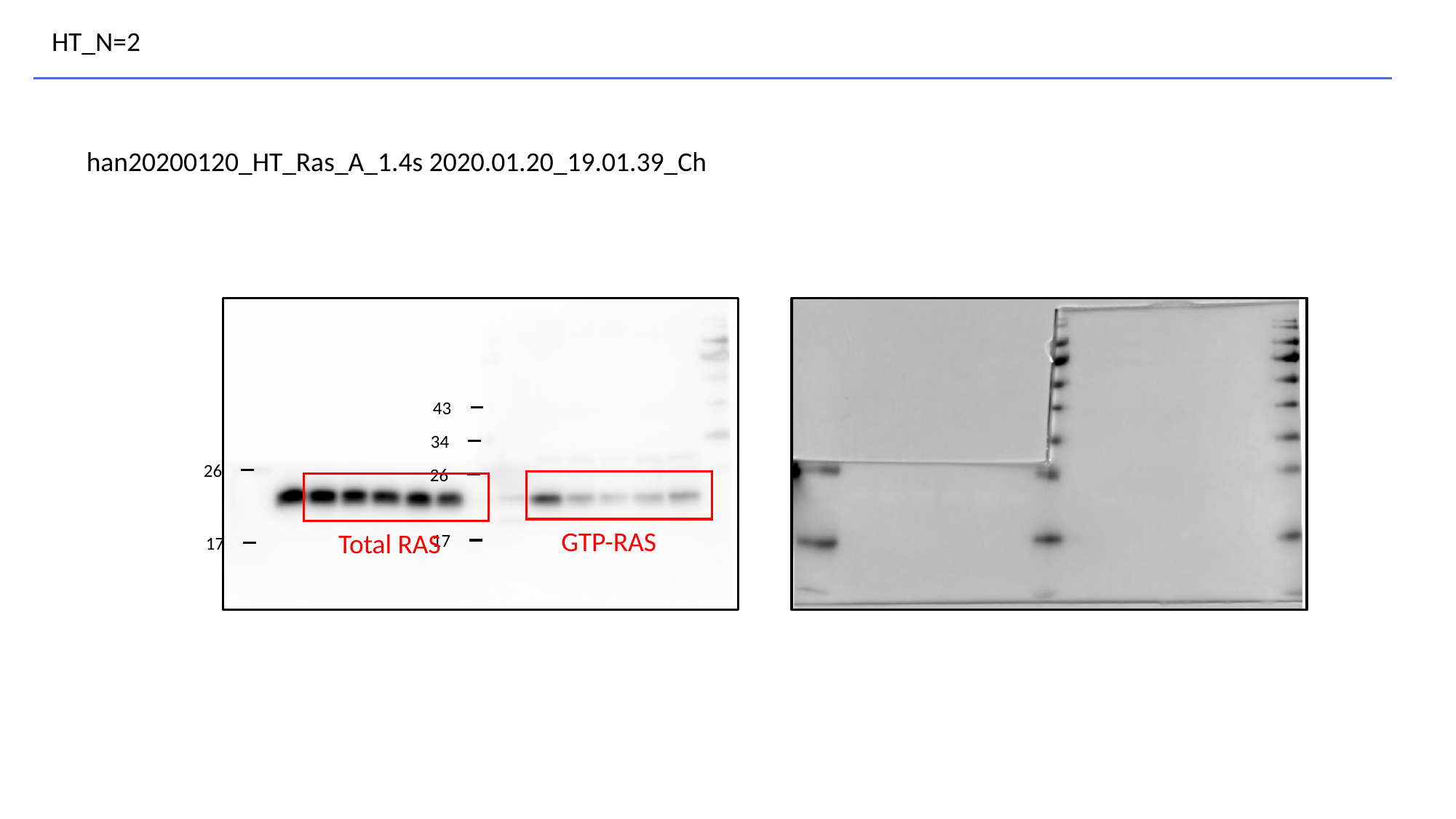

HT_N=2
han20200120_HT_Ras_A_1.4s 2020.01.20_19.01.39_Ch
43
34
26
26
GTP-RAS
Total RAS
17
17

## Slide 40
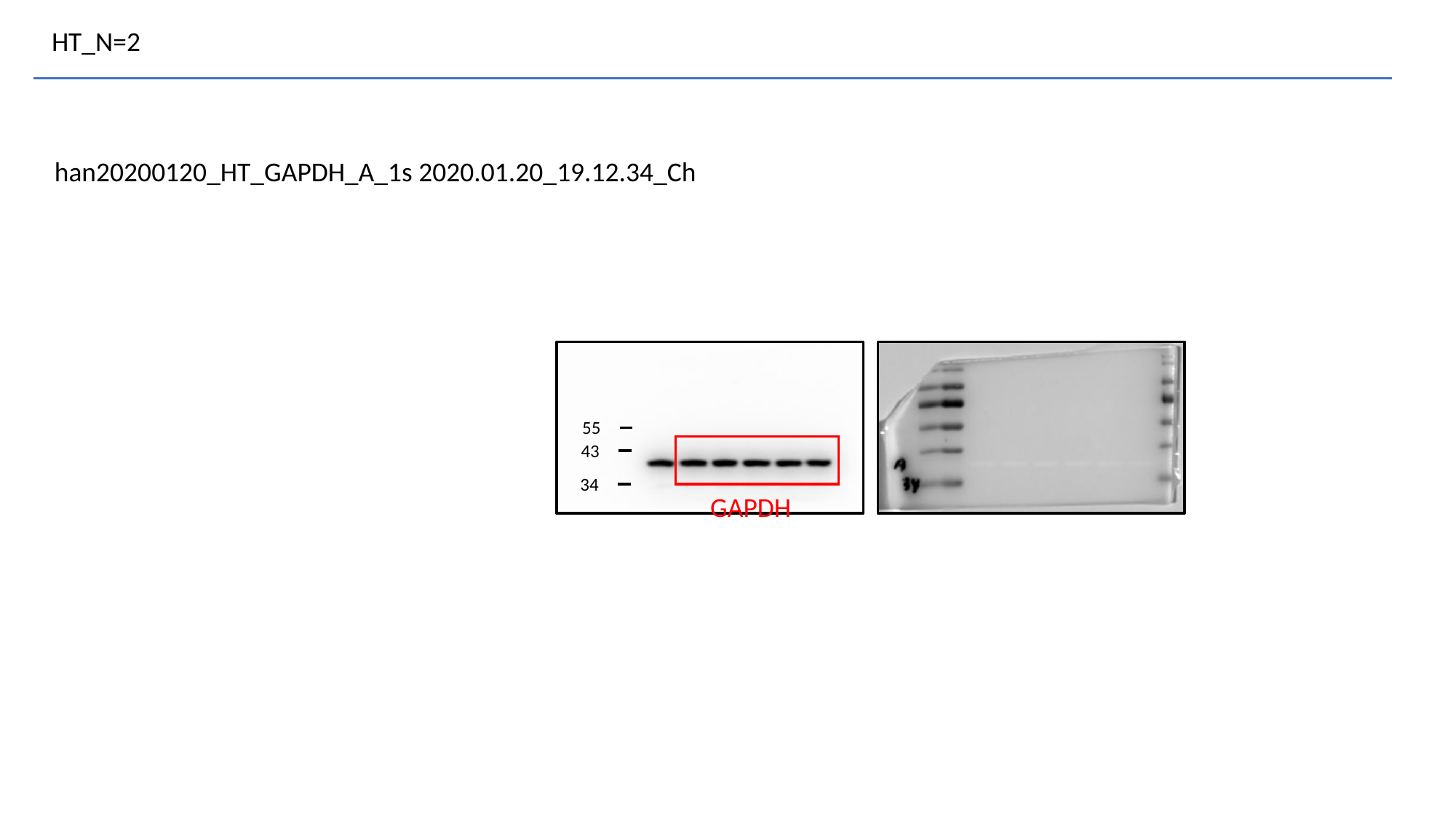

HT_N=2
han20200120_HT_GAPDH_A_1s 2020.01.20_19.12.34_Ch
55
43
34
GAPDH

## Slide 41
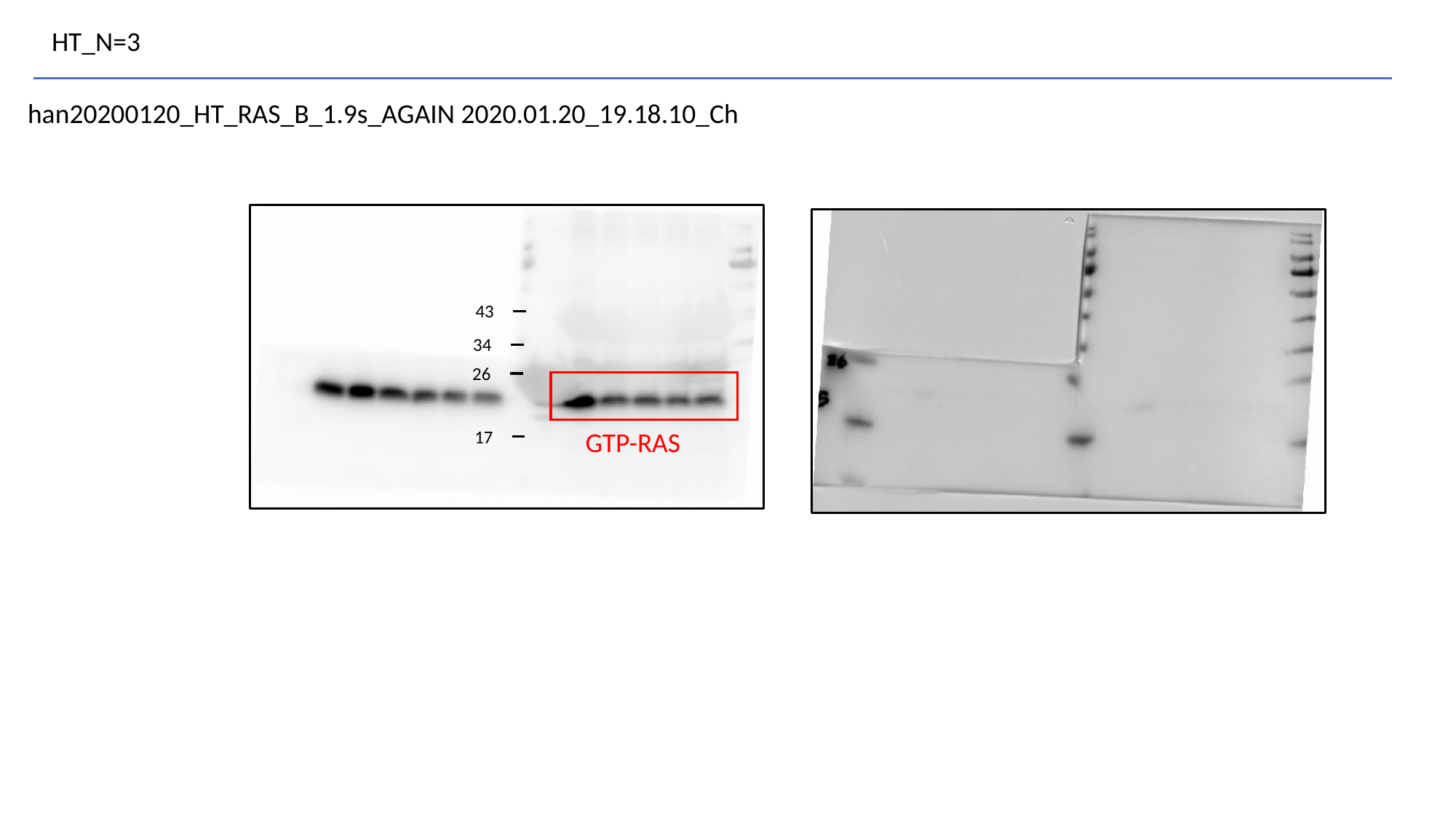

HT_N=3
han20200120_HT_RAS_B_1.9s_AGAIN 2020.01.20_19.18.10_Ch
43
34
26
GTP-RAS
17

## Slide 42
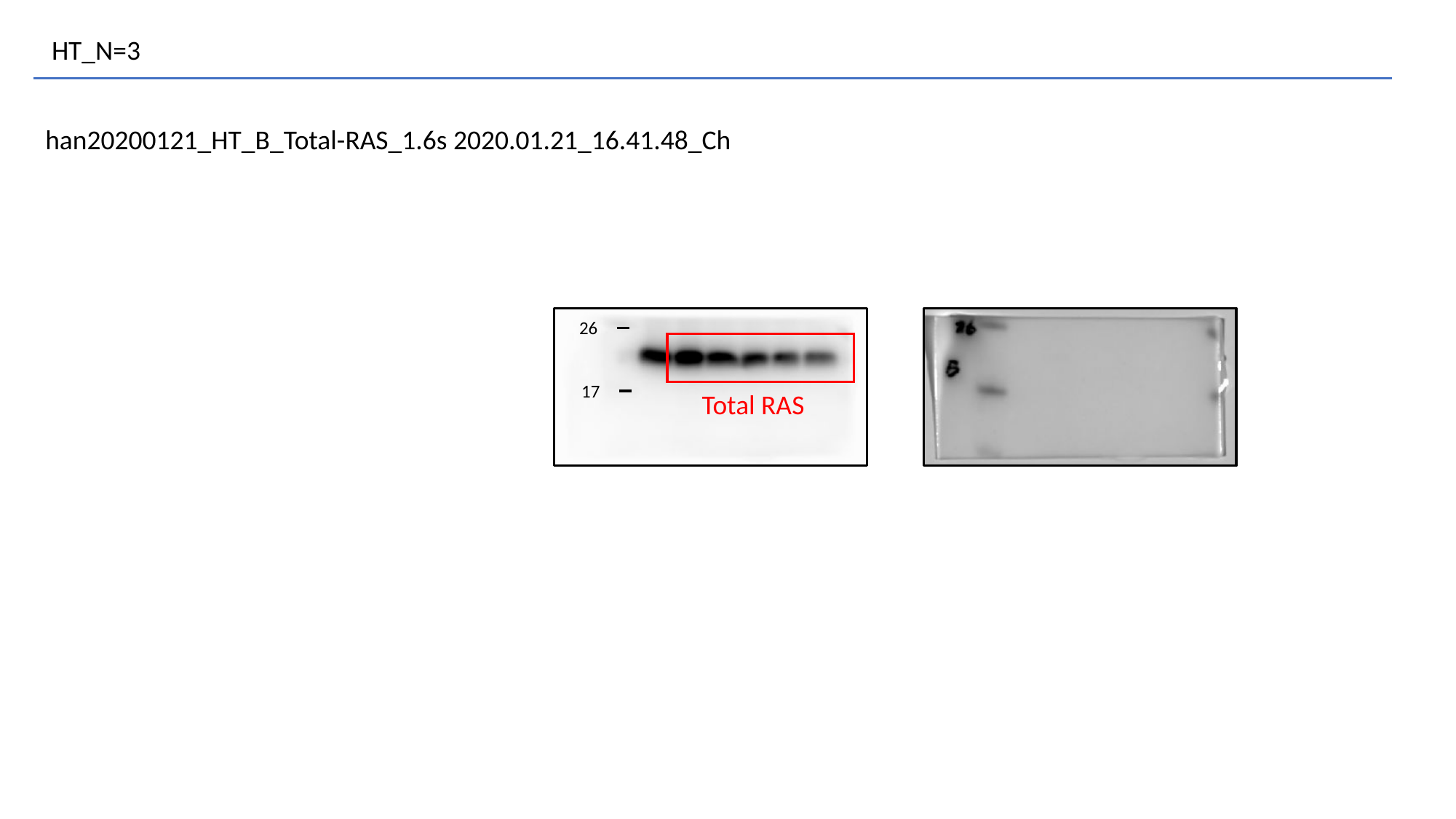

HT_N=3
han20200121_HT_B_Total-RAS_1.6s 2020.01.21_16.41.48_Ch
26
17
Total RAS

## Slide 43
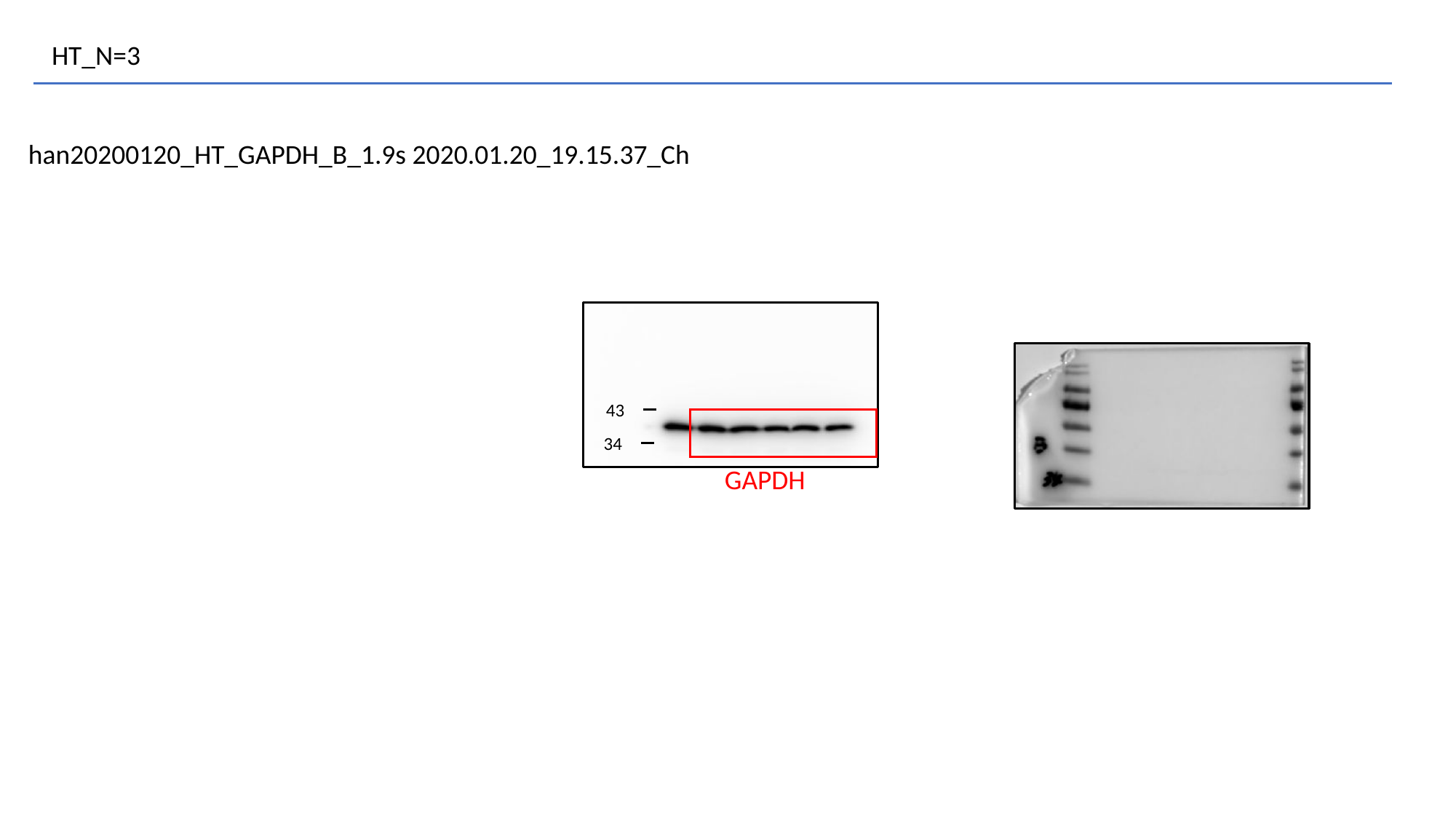

HT_N=3
han20200120_HT_GAPDH_B_1.9s 2020.01.20_19.15.37_Ch
43
34
GAPDH

## Slide 44
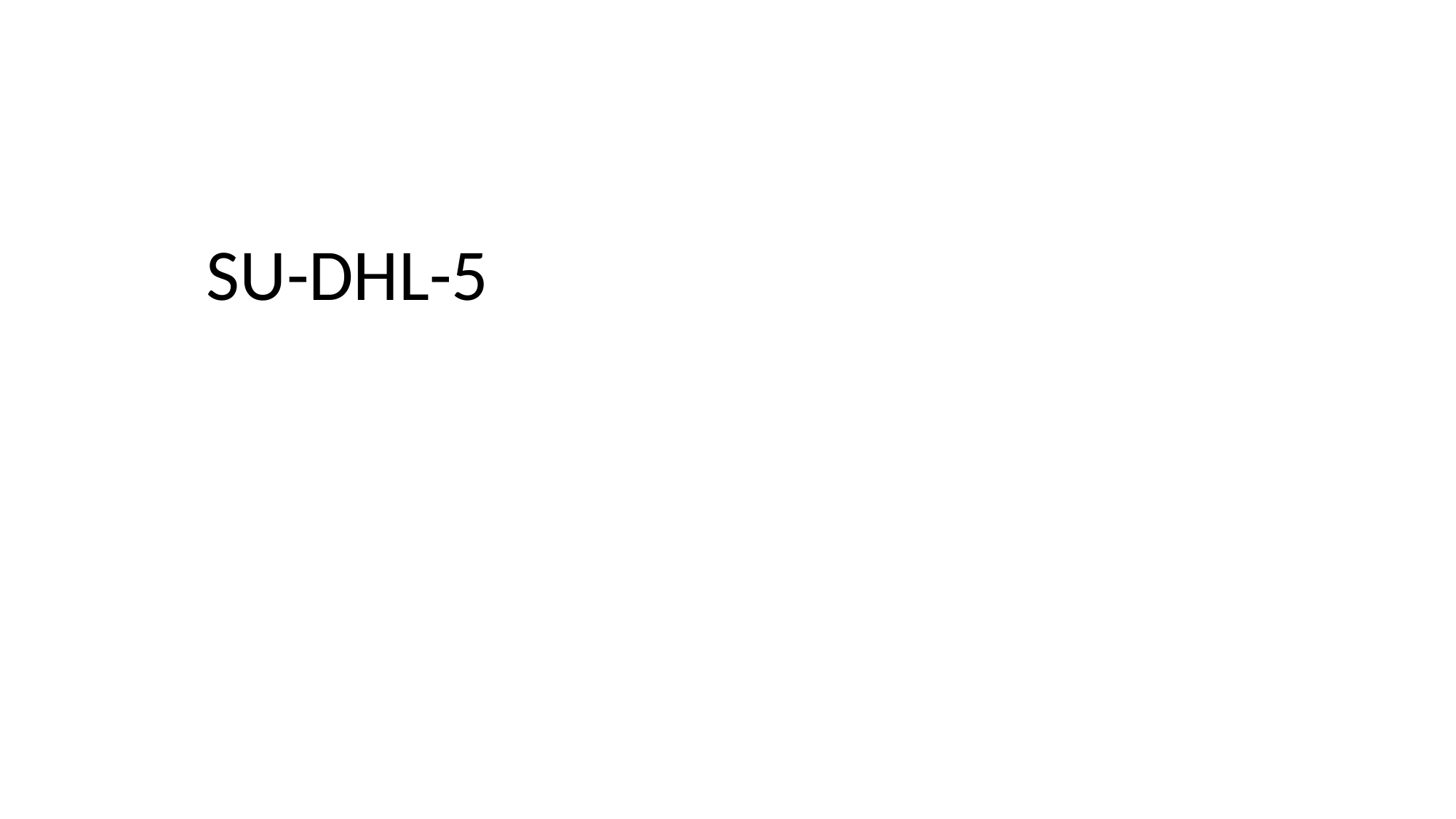

SU-DHL-5

## Slide 45
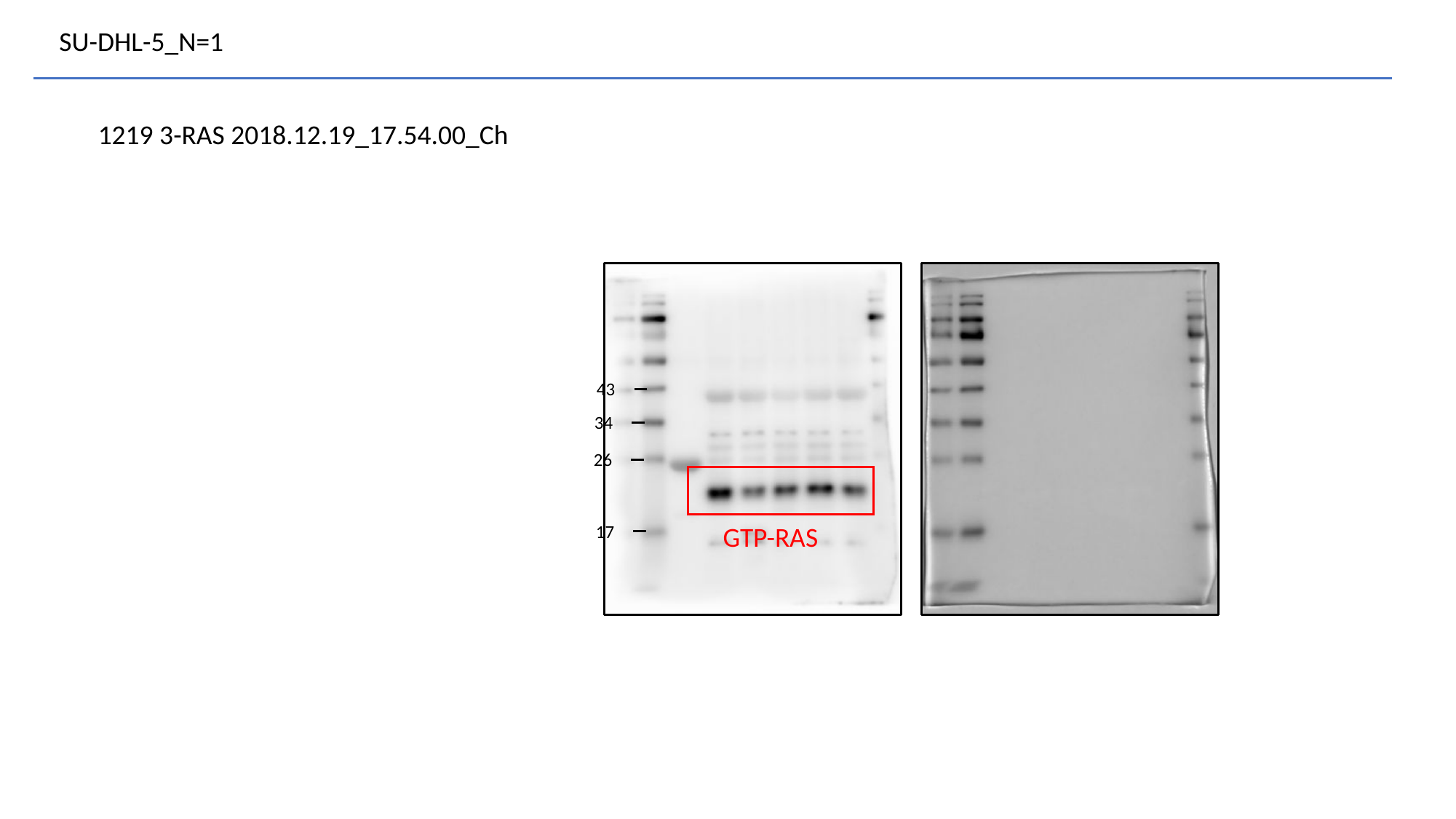

SU-DHL-5_N=1
1219 3-RAS 2018.12.19_17.54.00_Ch
43
34
26
17
GTP-RAS

## Slide 46
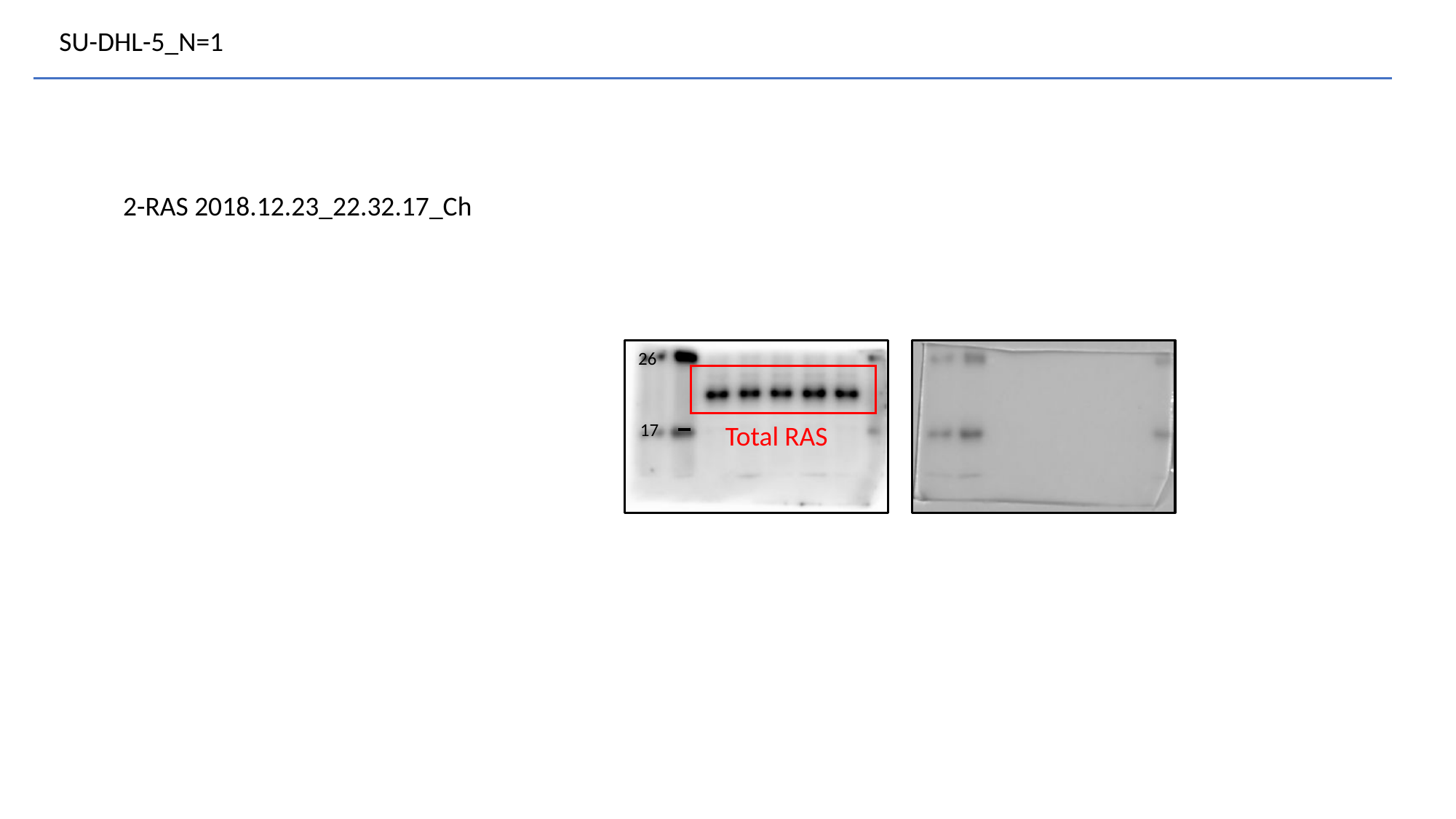

SU-DHL-5_N=1
2-RAS 2018.12.23_22.32.17_Ch
26
17
Total RAS

## Slide 47
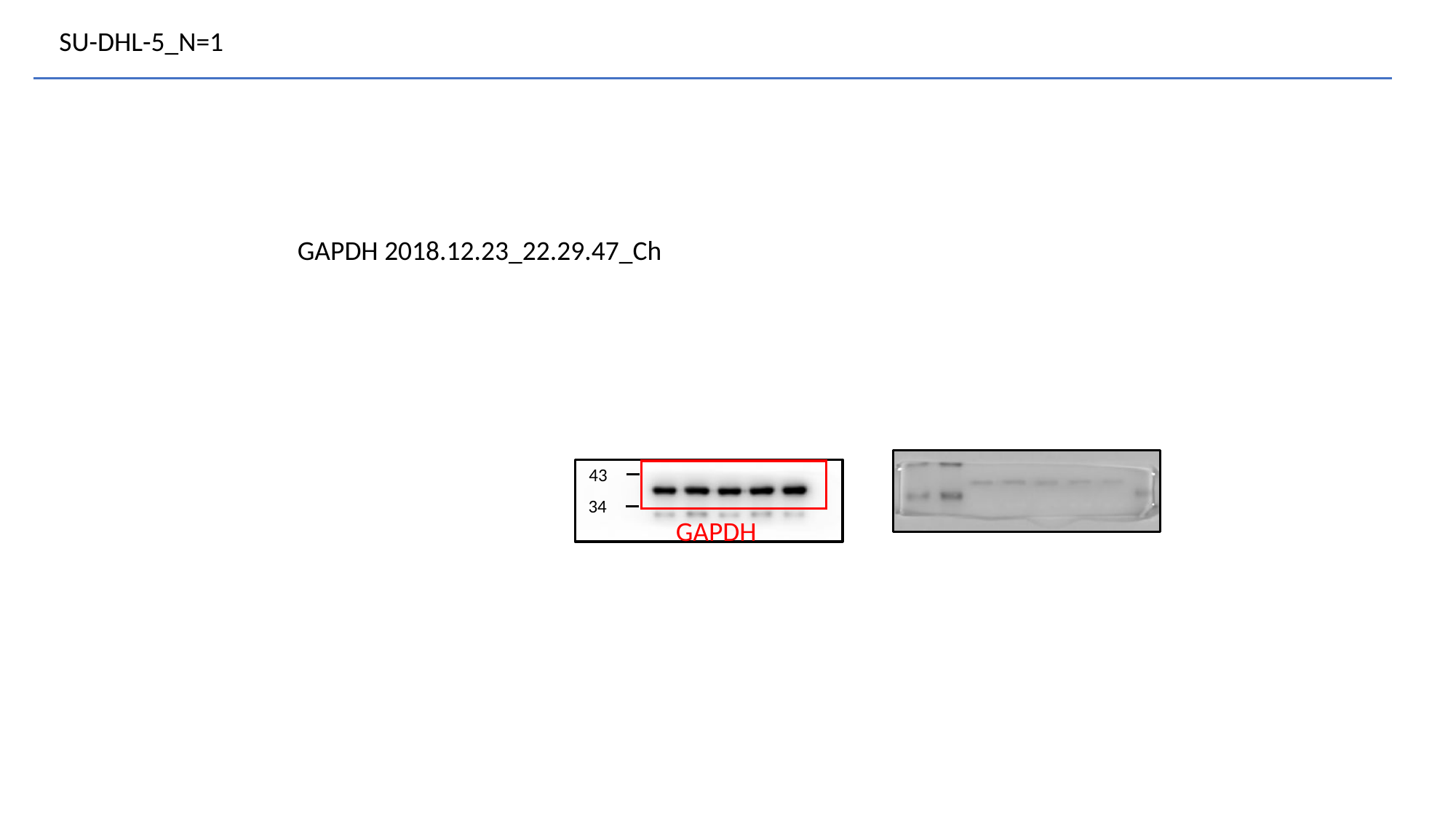

SU-DHL-5_N=1
GAPDH 2018.12.23_22.29.47_Ch
43
34
GAPDH

## Slide 48
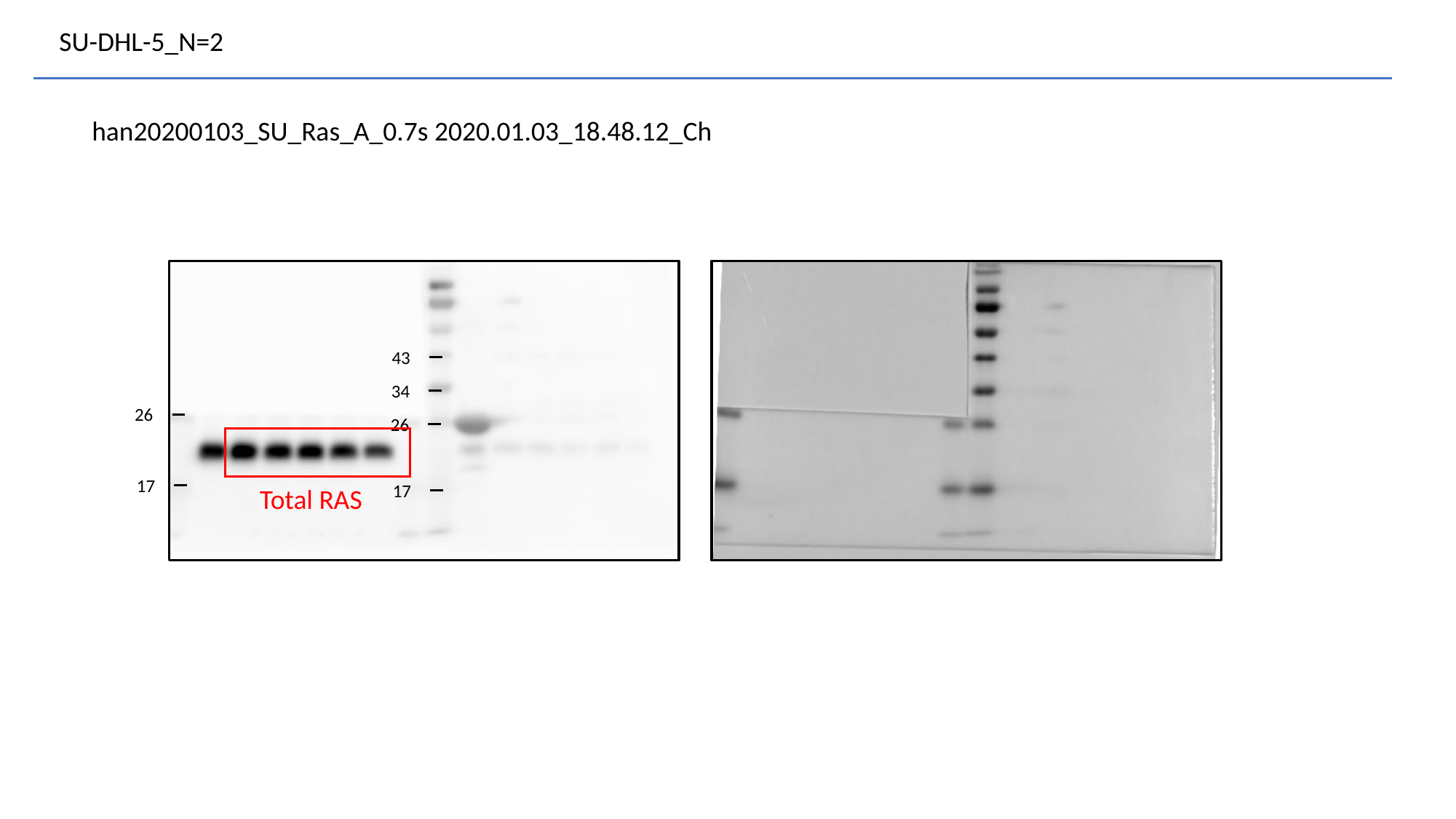

SU-DHL-5_N=2
han20200103_SU_Ras_A_0.7s 2020.01.03_18.48.12_Ch
43
34
26
26
17
17
Total RAS

## Slide 49
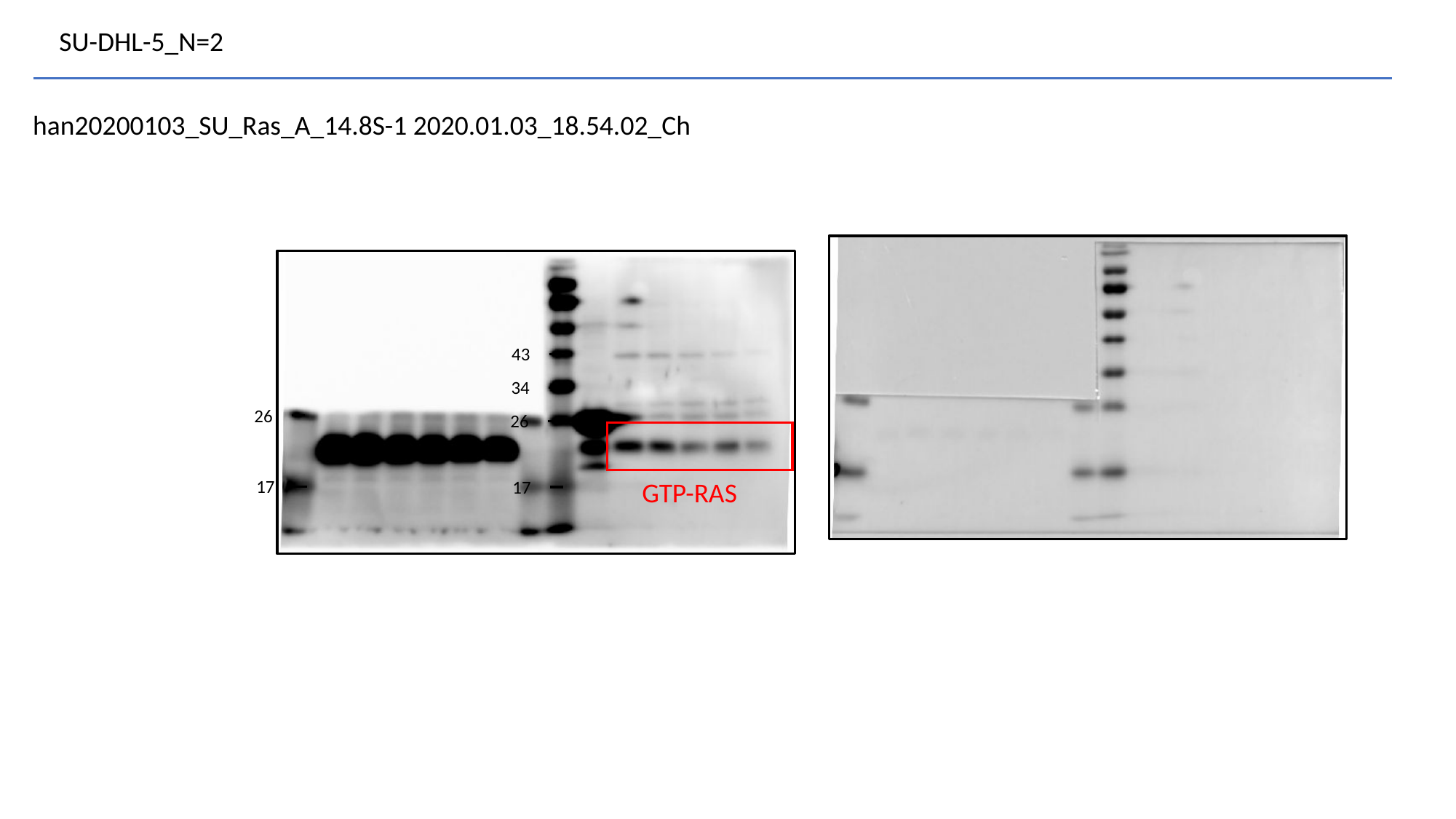

SU-DHL-5_N=2
han20200103_SU_Ras_A_14.8S-1 2020.01.03_18.54.02_Ch
43
34
26
26
17
GTP-RAS
17

## Slide 50
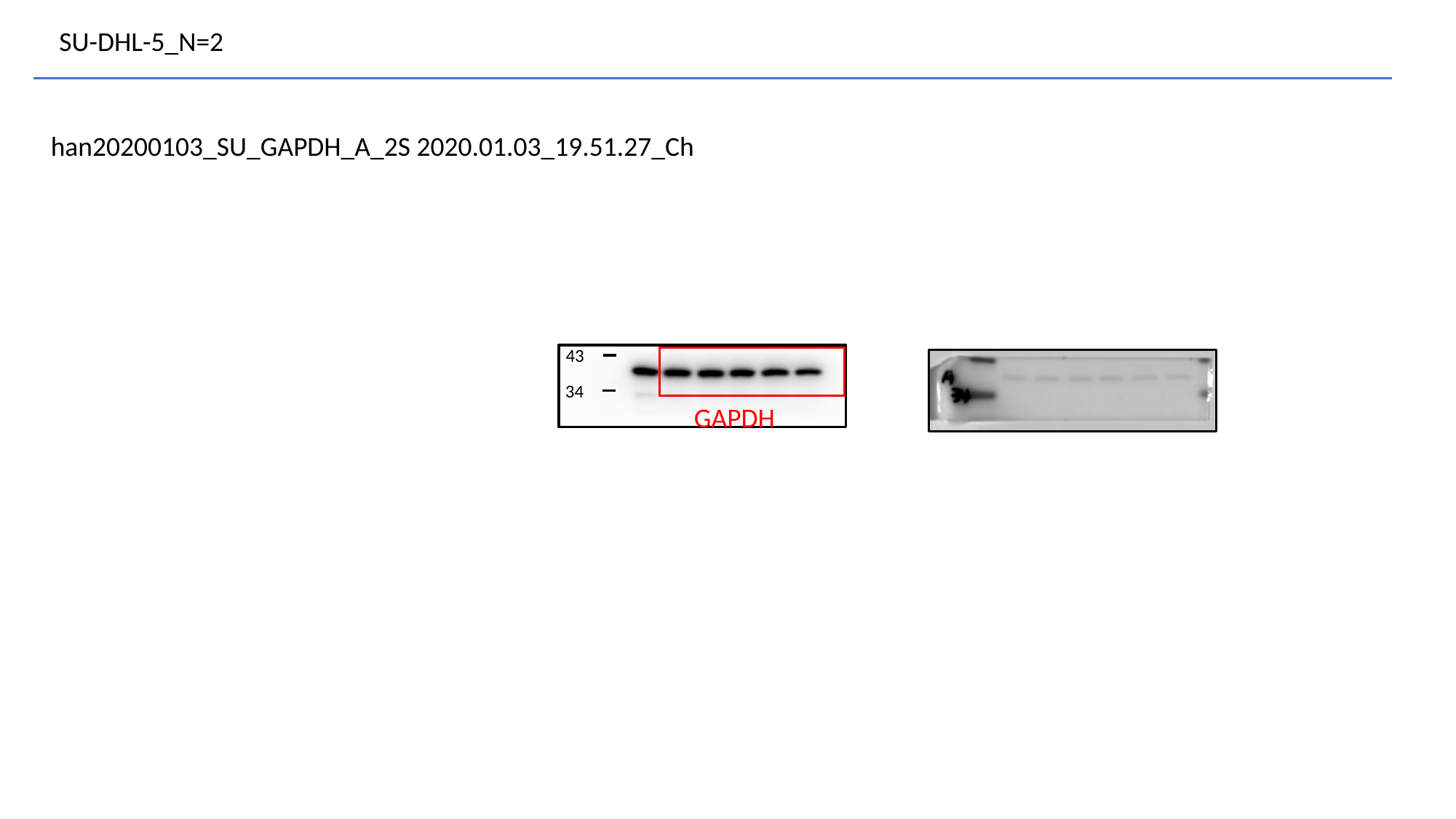

SU-DHL-5_N=2
han20200103_SU_GAPDH_A_2S 2020.01.03_19.51.27_Ch
43
34
GAPDH

## Slide 51
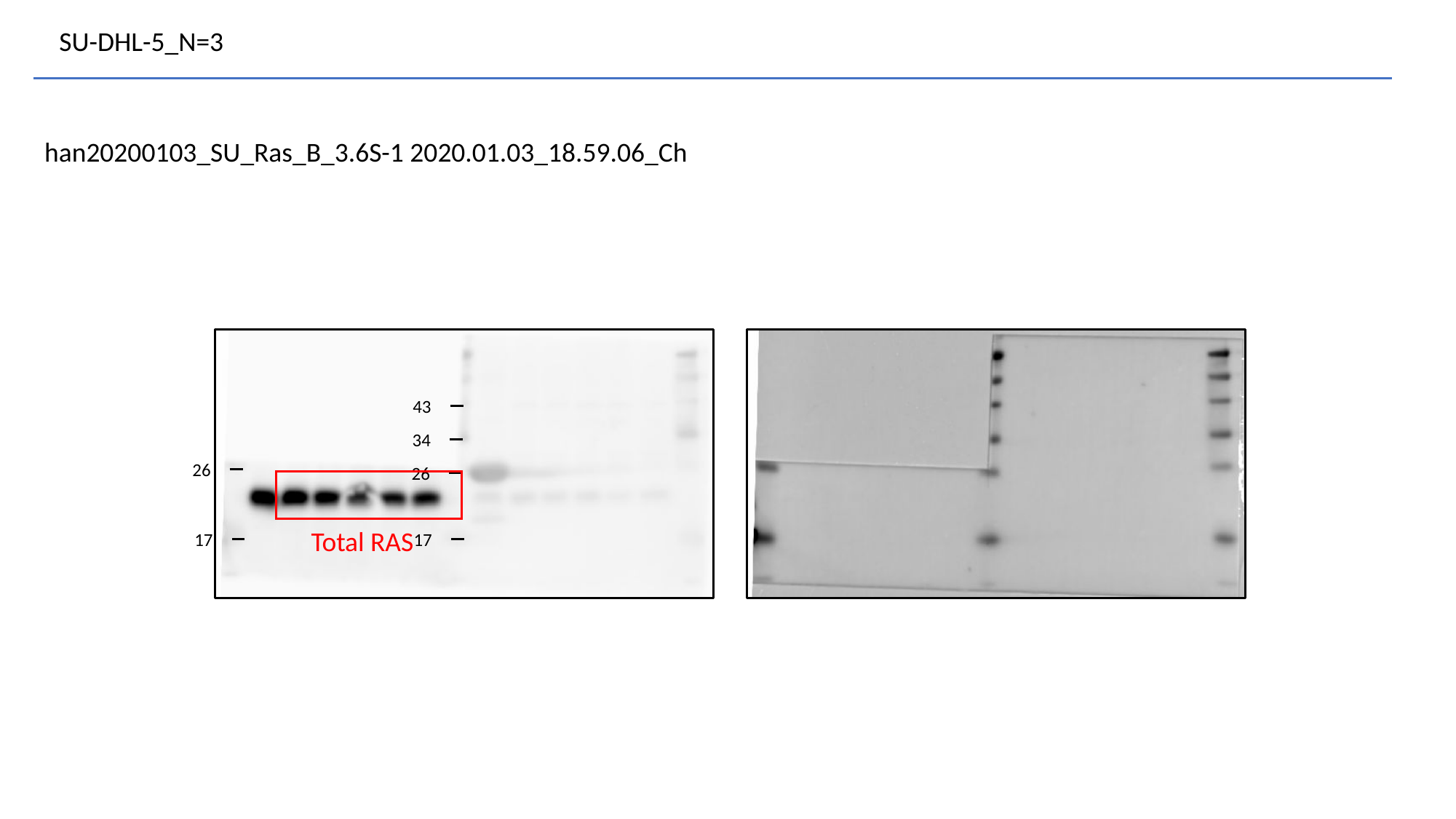

SU-DHL-5_N=3
han20200103_SU_Ras_B_3.6S-1 2020.01.03_18.59.06_Ch
43
34
26
26
Total RAS
17
17

## Slide 52
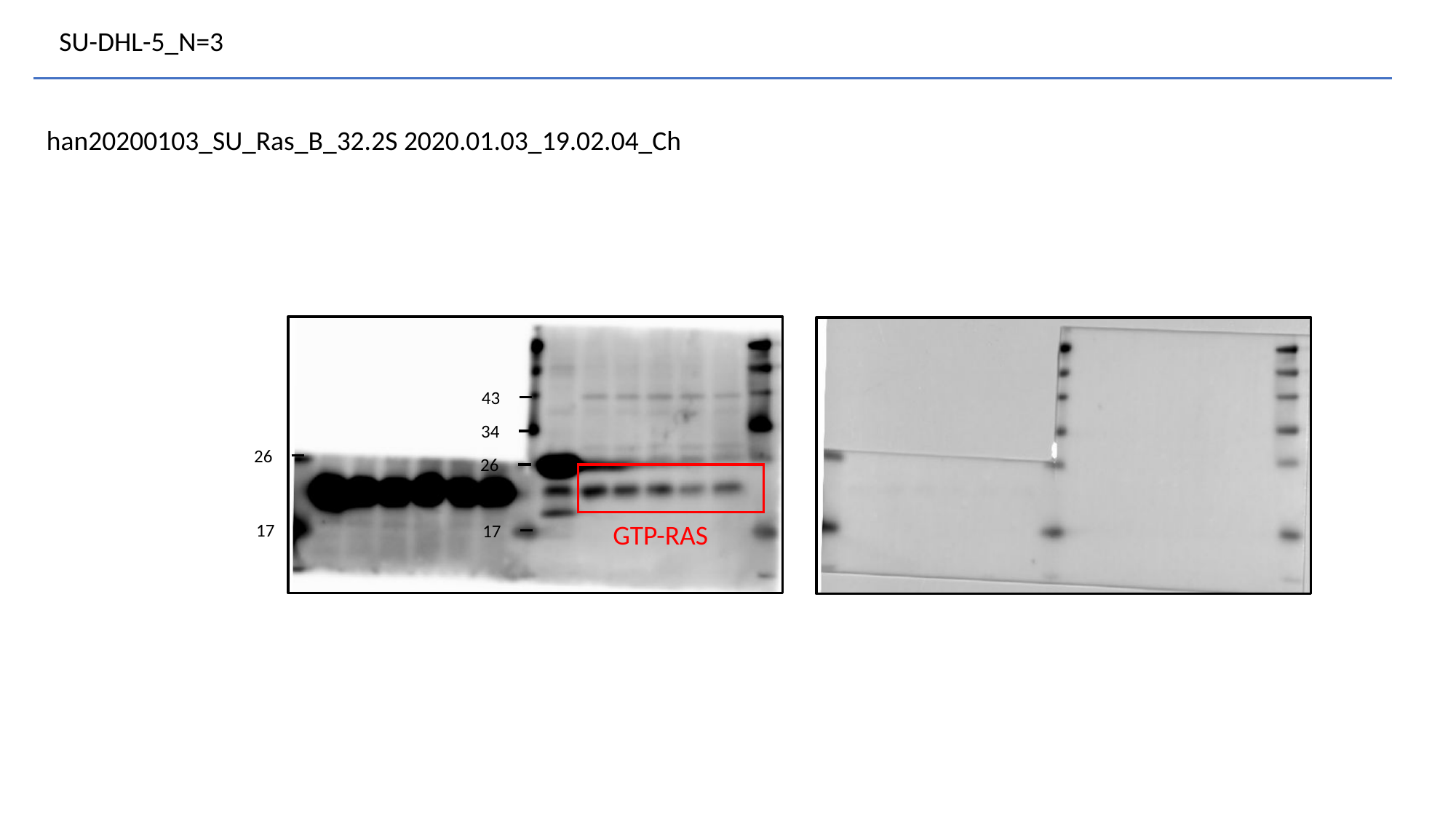

SU-DHL-5_N=3
han20200103_SU_Ras_B_32.2S 2020.01.03_19.02.04_Ch
43
34
26
26
GTP-RAS
17
17

## Slide 53
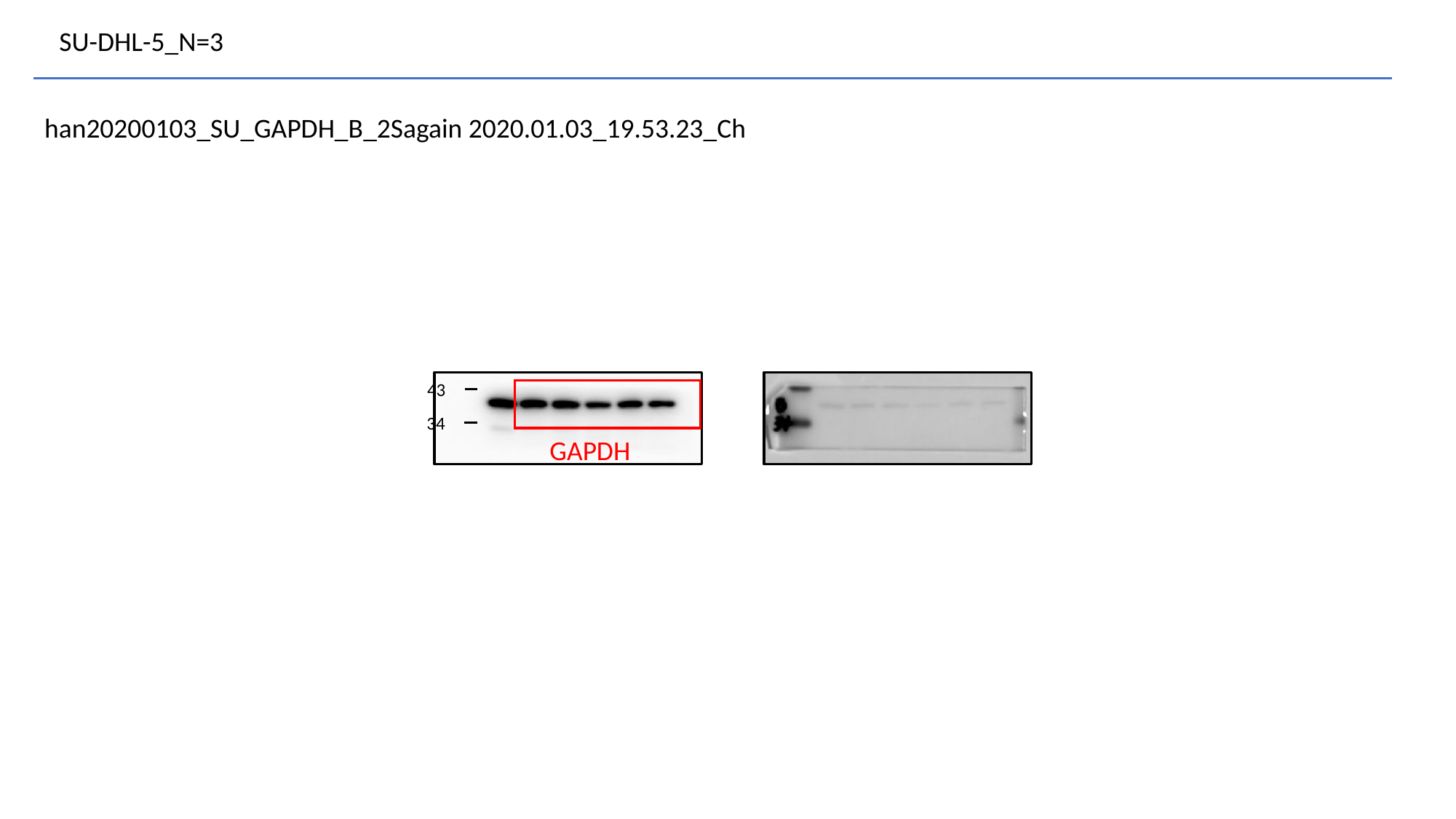

SU-DHL-5_N=3
han20200103_SU_GAPDH_B_2Sagain 2020.01.03_19.53.23_Ch
43
34
GAPDH

## Slide 54
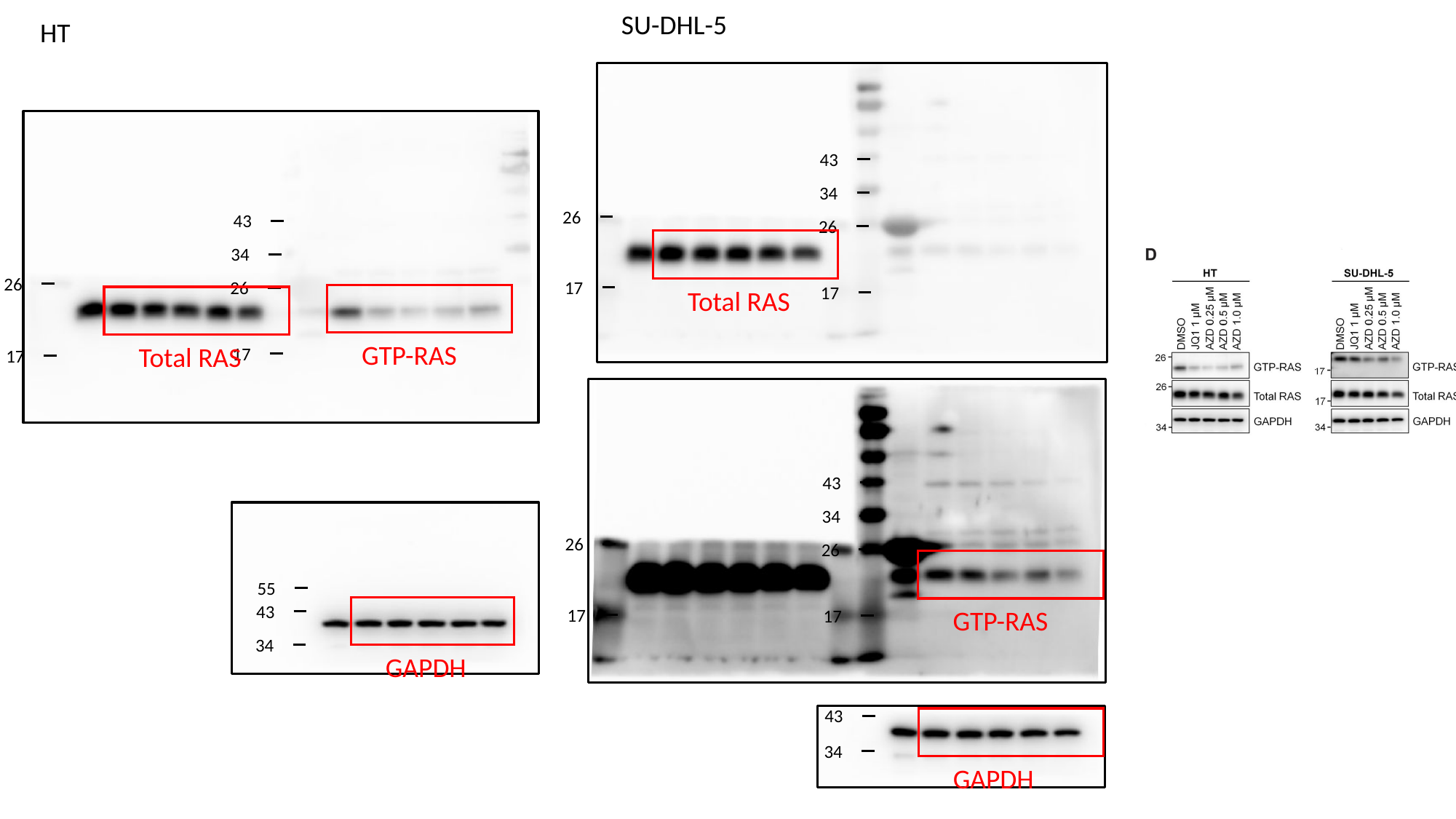

SU-DHL-5
HT
43
34
26
43
26
34
26
17
26
17
Total RAS
GTP-RAS
Total RAS
17
17
43
34
26
26
55
43
17
GTP-RAS
17
34
GAPDH
43
34
GAPDH

## Slide 55
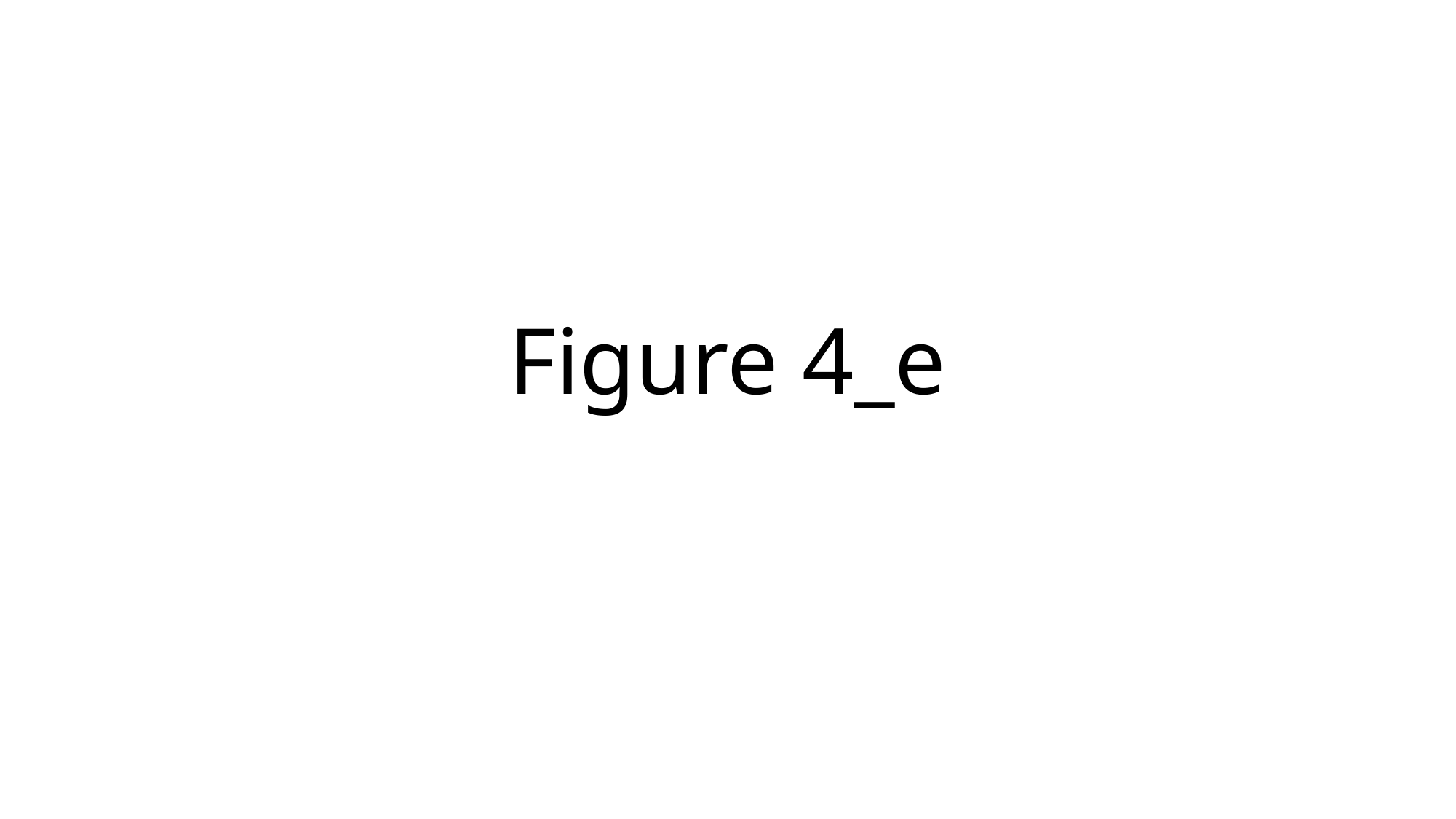

# Figure 4_e

## Slide 56
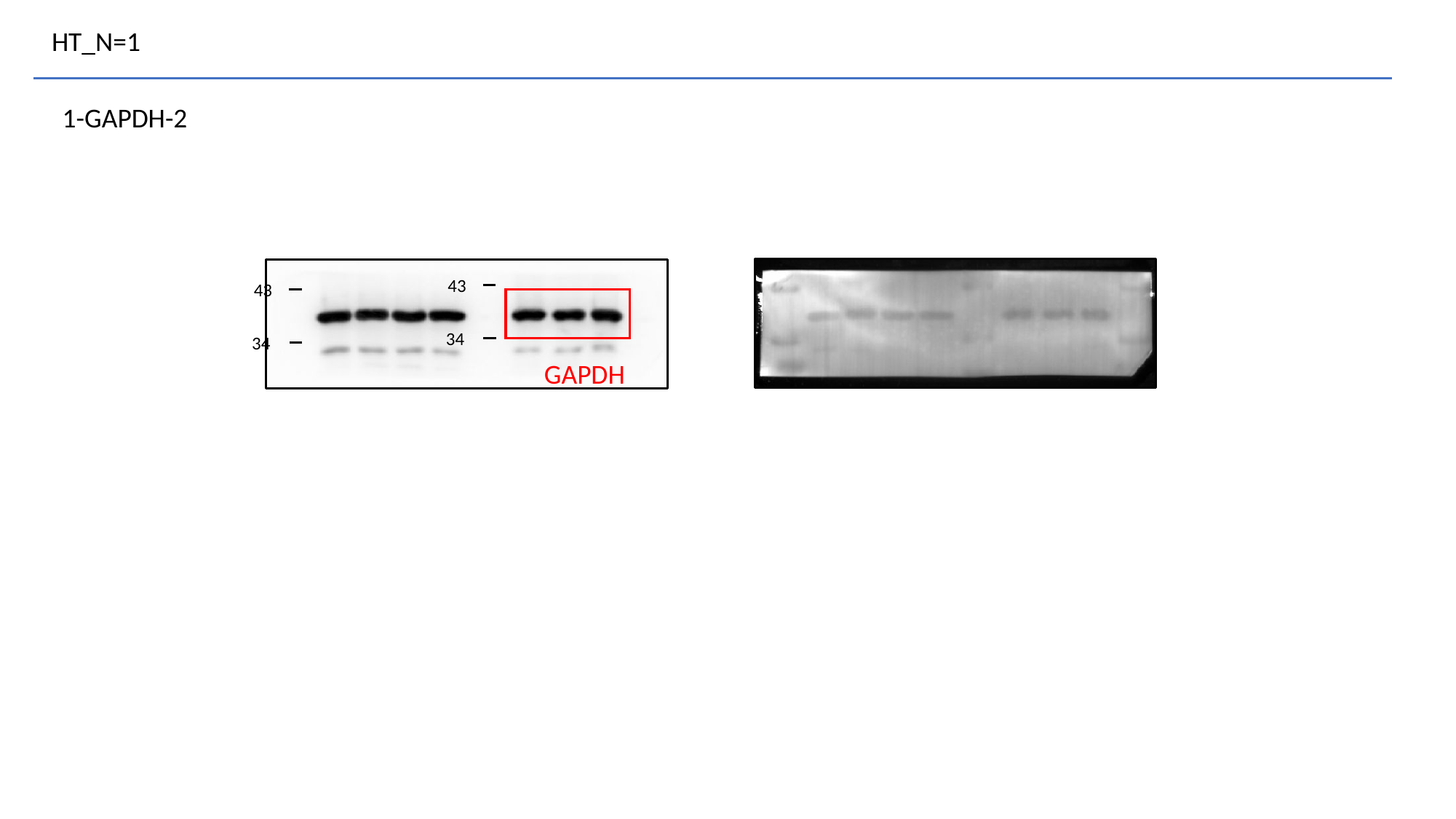

HT_N=1
1-GAPDH-2
43
43
34
34
GAPDH

## Slide 57
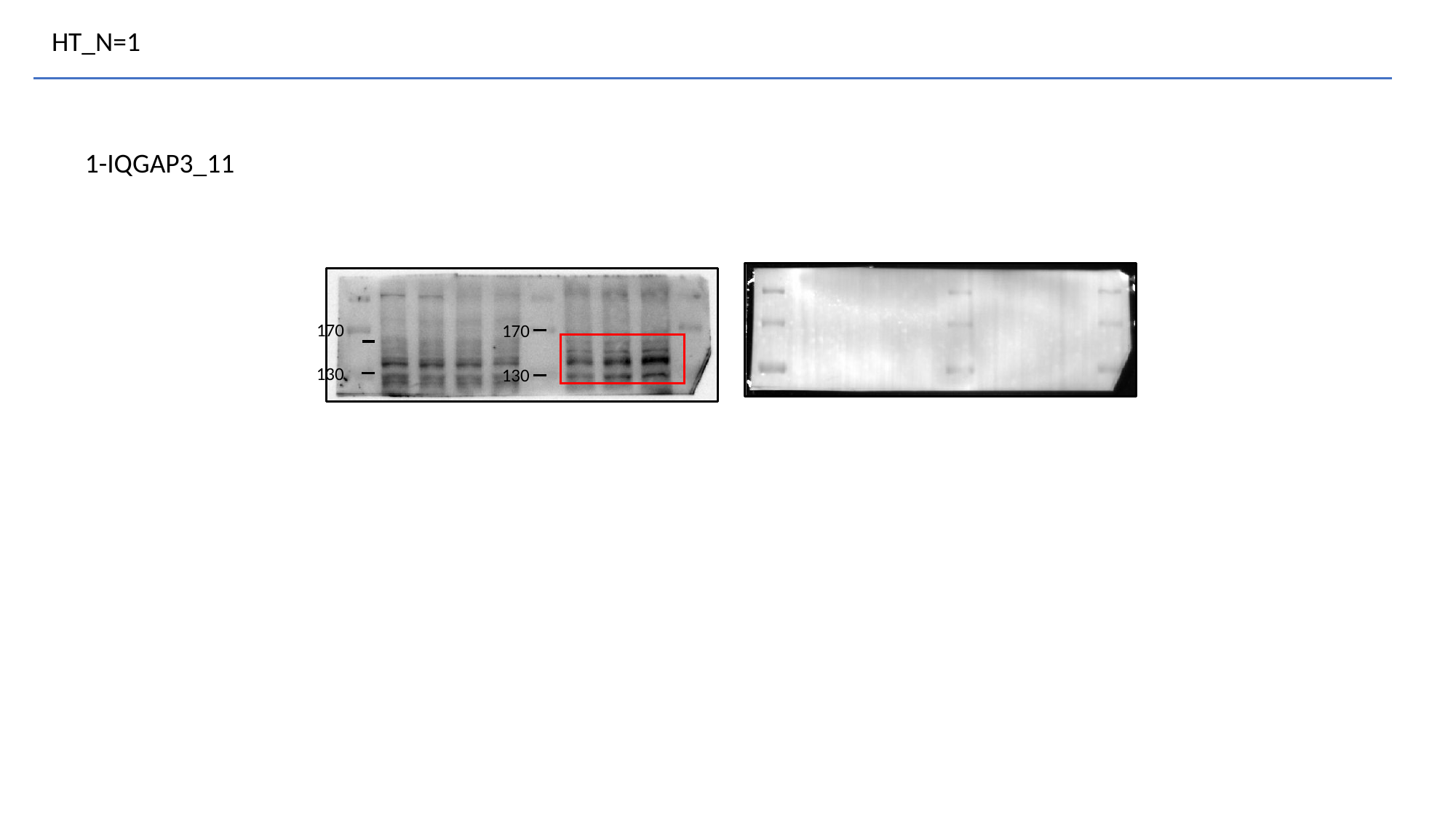

HT_N=1
43
43
1-GAPDH-2
34
34
GAPDH
1-IQGAP3_11
170
170
170
170
130
130
130
130
1-IQGAP3_11
95
95

## Slide 58
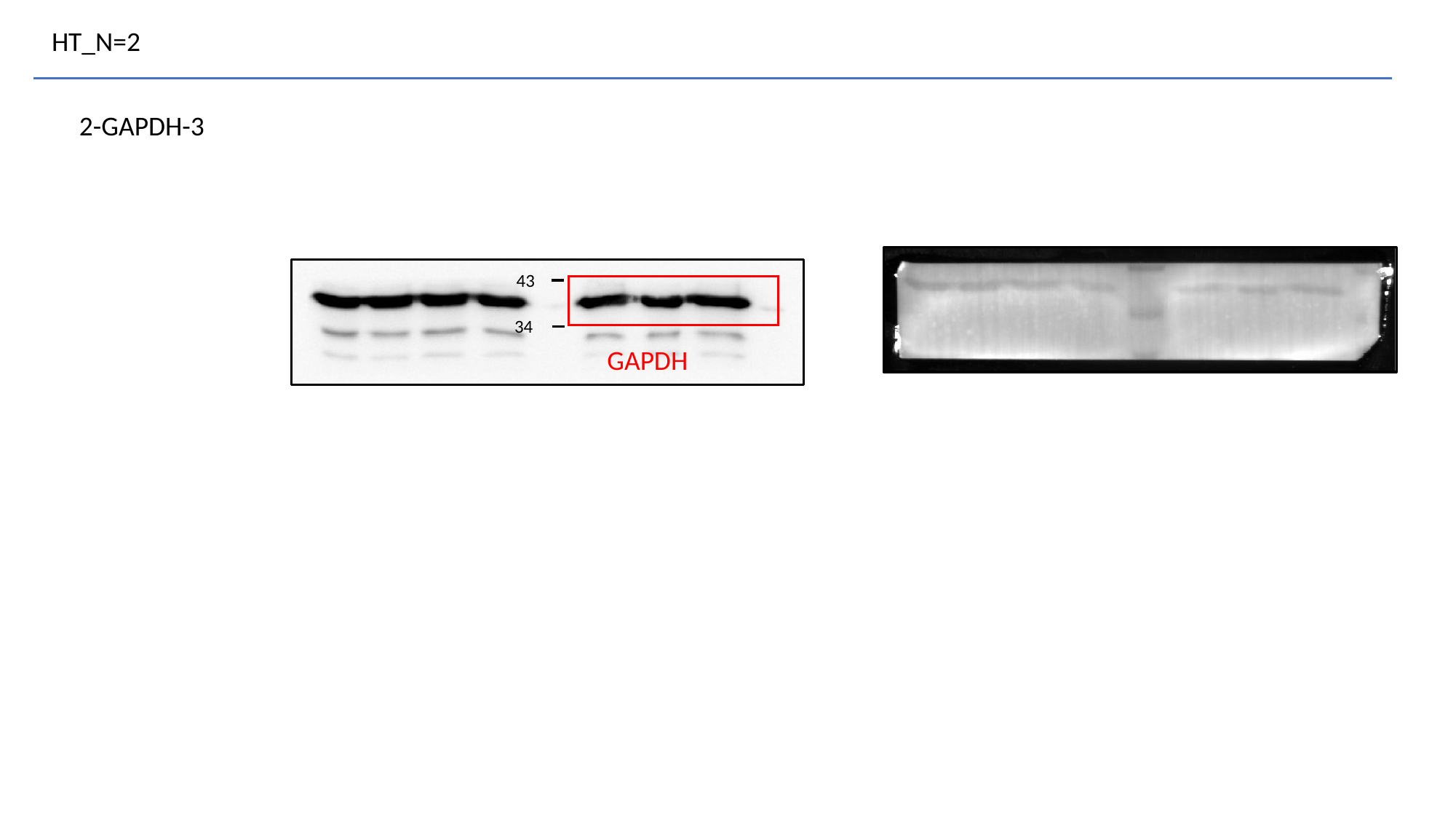

HT_N=2
2-GAPDH-3
43
34
GAPDH

## Slide 59
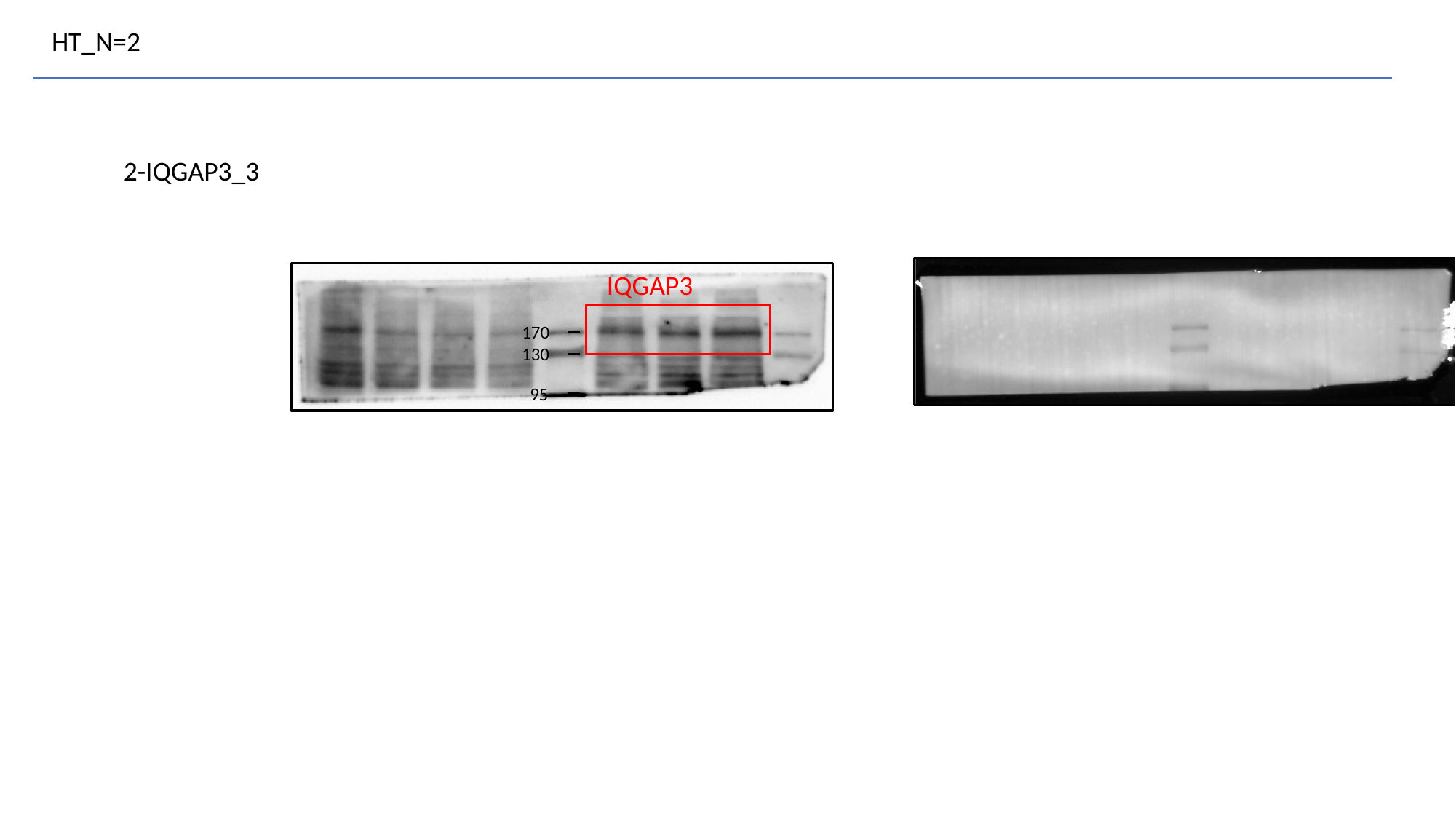

HT_N=2
2-IQGAP3_3
IQGAP3
170
130
95

## Slide 60
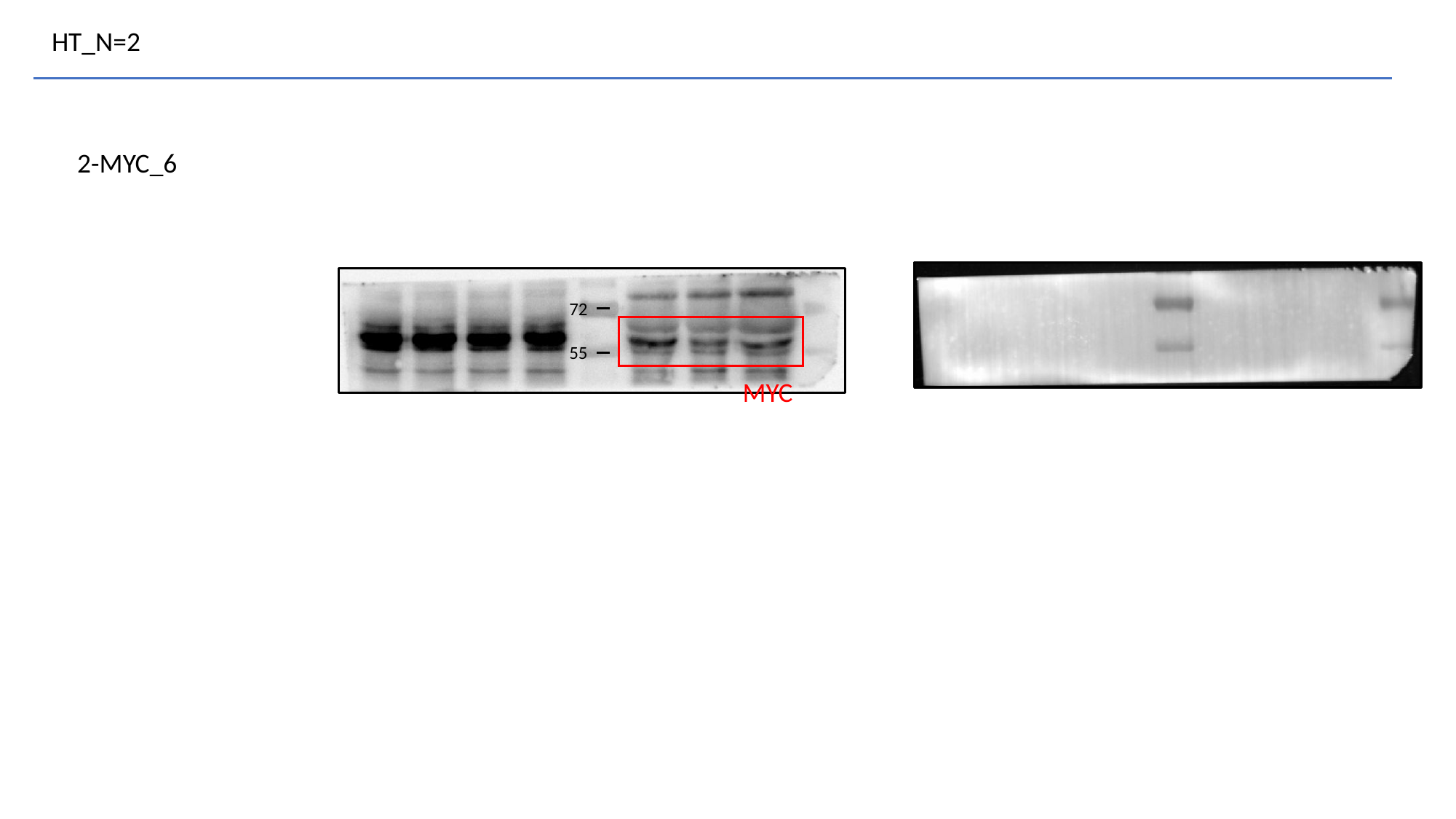

HT_N=2
2-MYC_6
72
55
MYC

## Slide 61
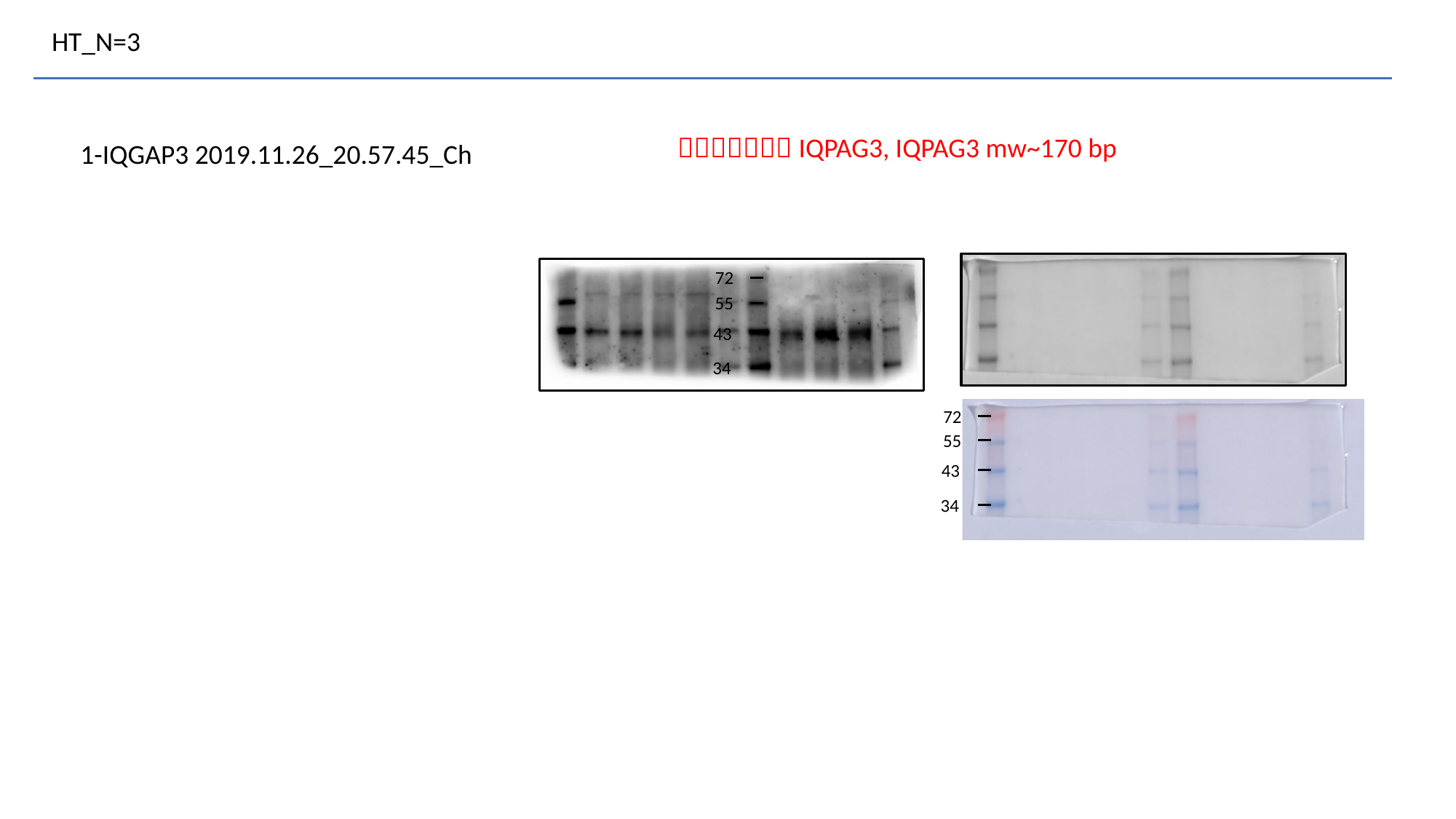

HT_N=3
我不認為這張是IQPAG3, IQPAG3 mw~170 bp
1-IQGAP3 2019.11.26_20.57.45_Ch
72
55
43
34
72
55
43
34

## Slide 62
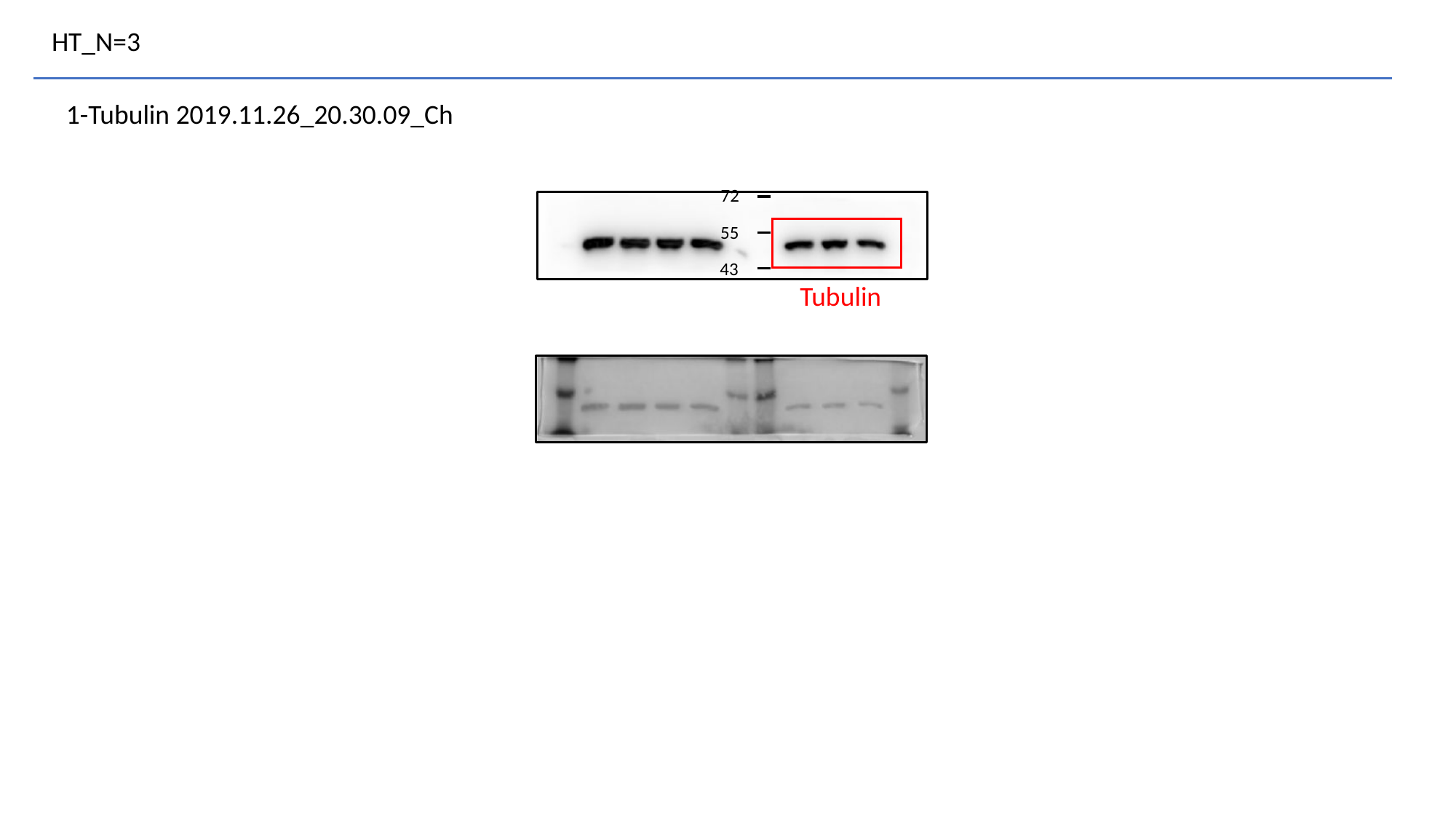

HT_N=3
1-Tubulin 2019.11.26_20.30.09_Ch
72
55
43
Tubulin

## Slide 63
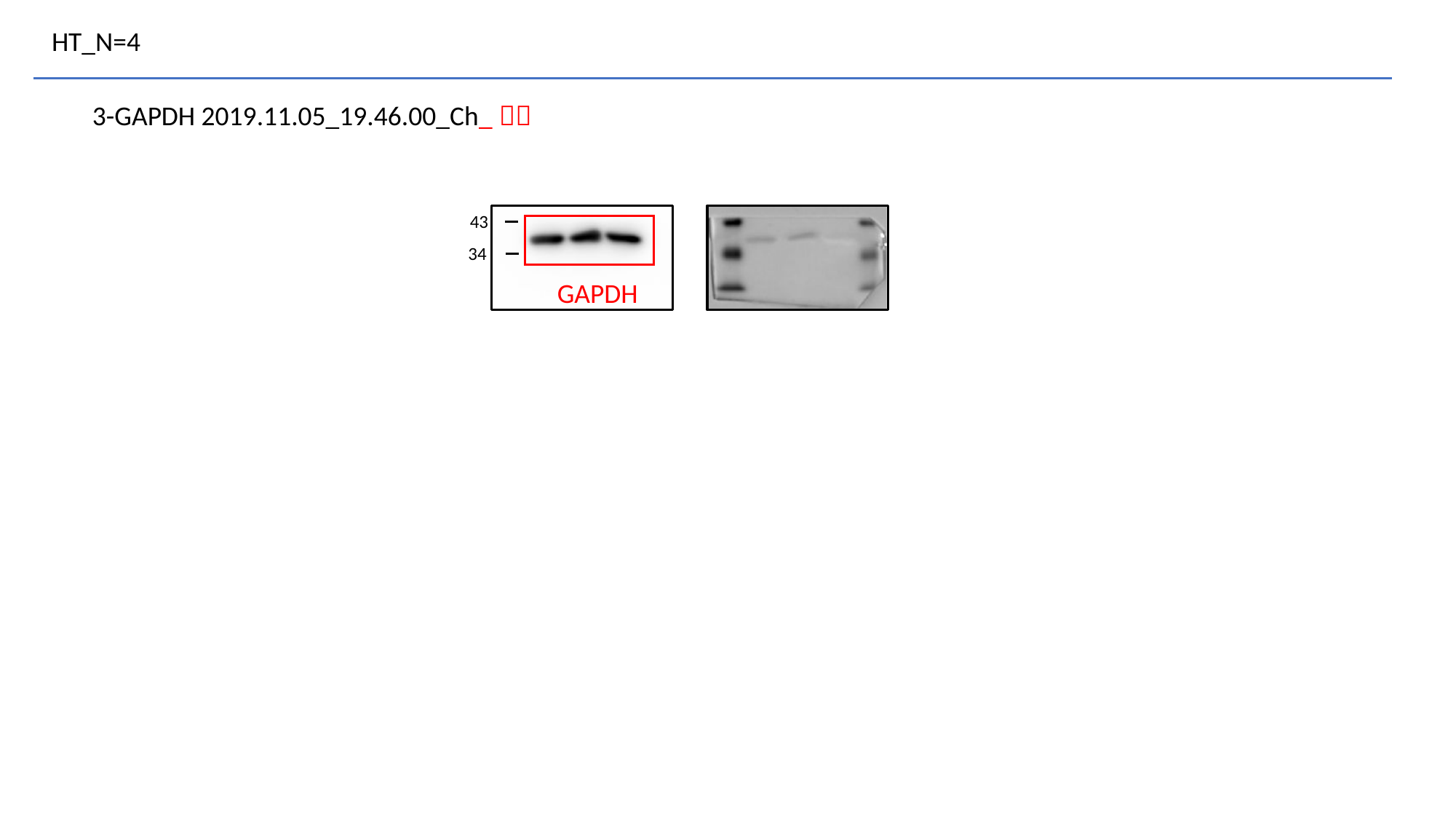

HT_N=4
3-GAPDH 2019.11.05_19.46.00_Ch_下圖
43
34
GAPDH

## Slide 64
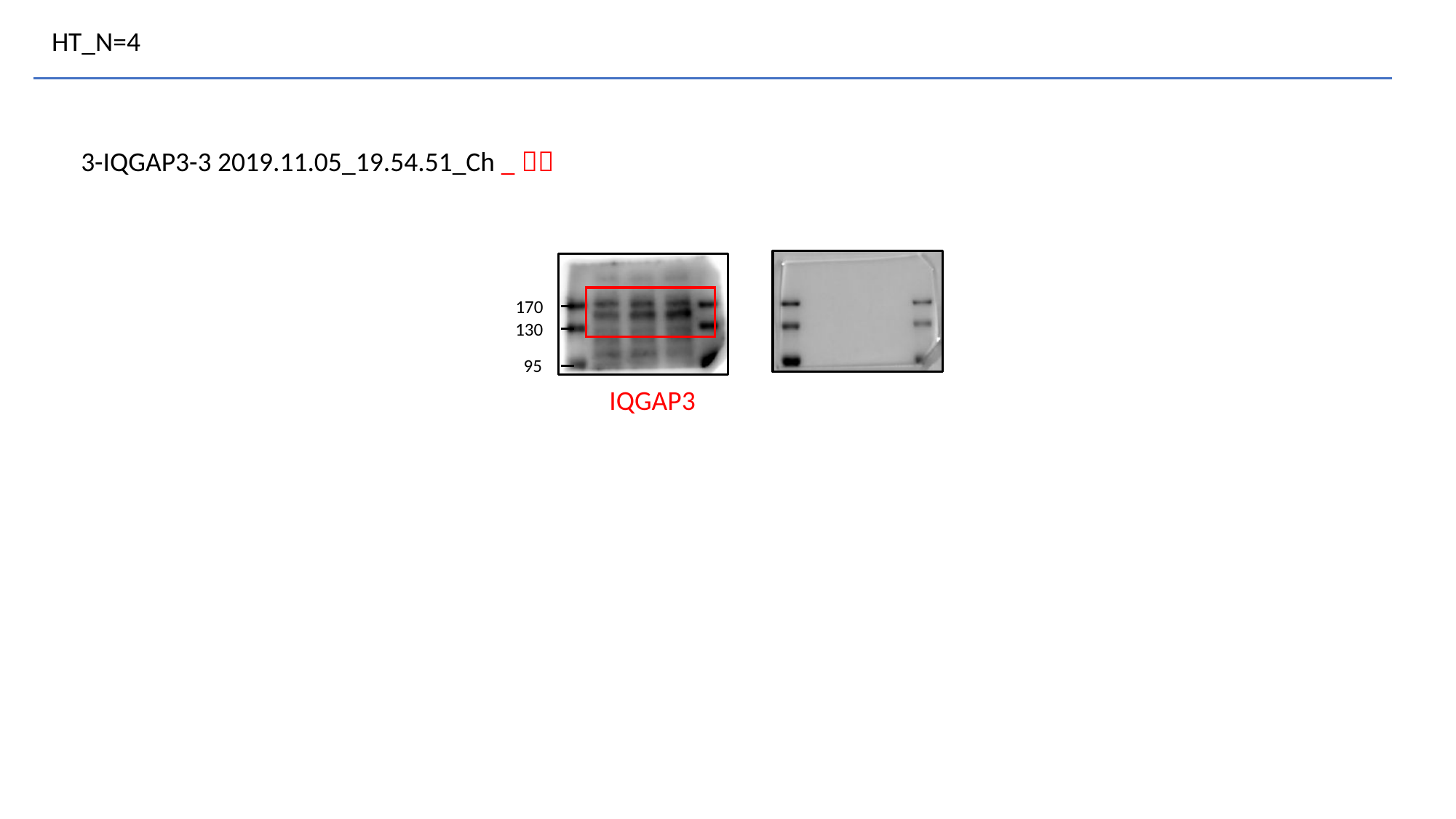

HT_N=4
3-IQGAP3-3 2019.11.05_19.54.51_Ch _下圖
170
130
95
IQGAP3

## Slide 65
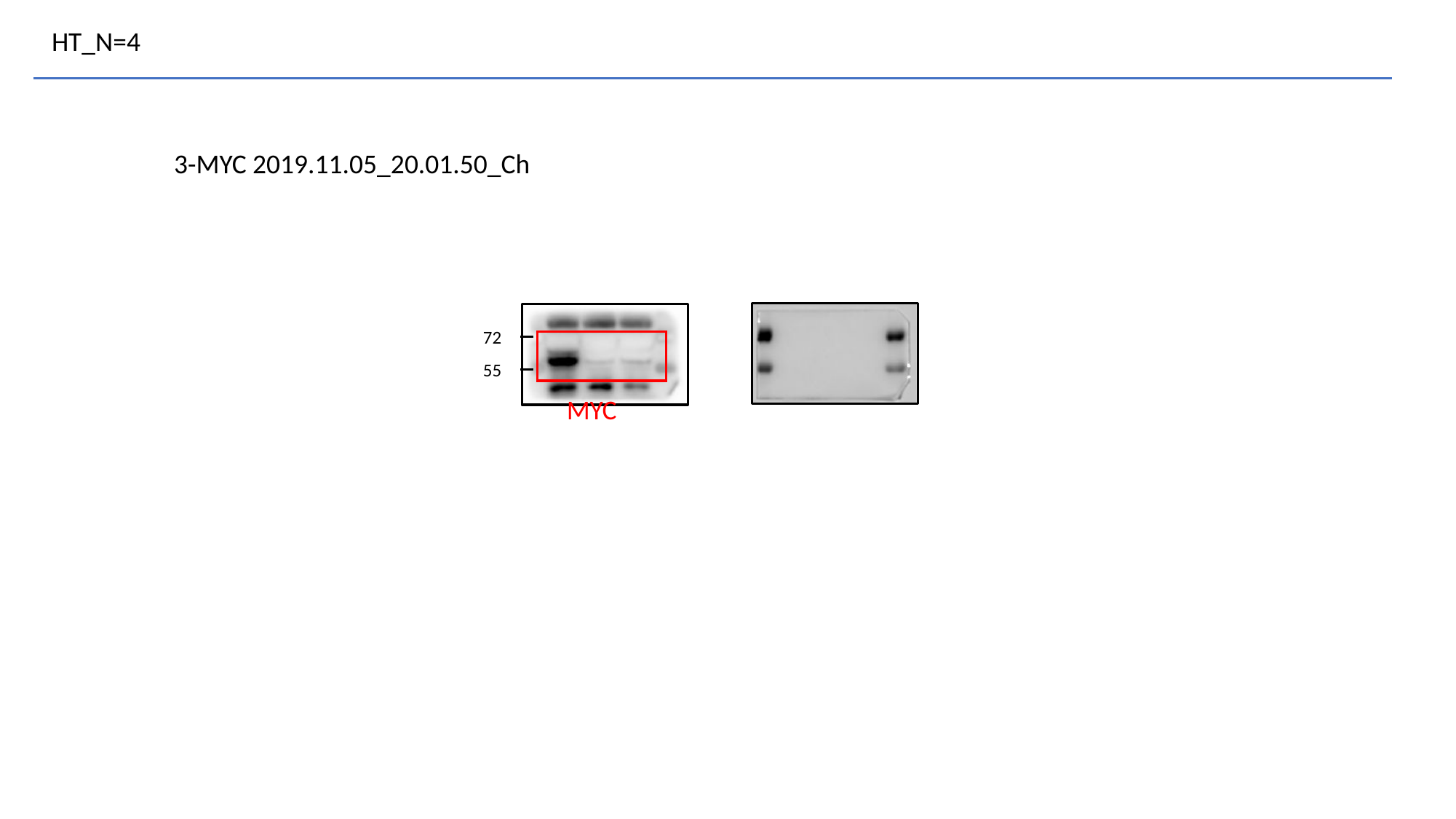

HT_N=4
3-MYC 2019.11.05_20.01.50_Ch
72
55
MYC

## Slide 66
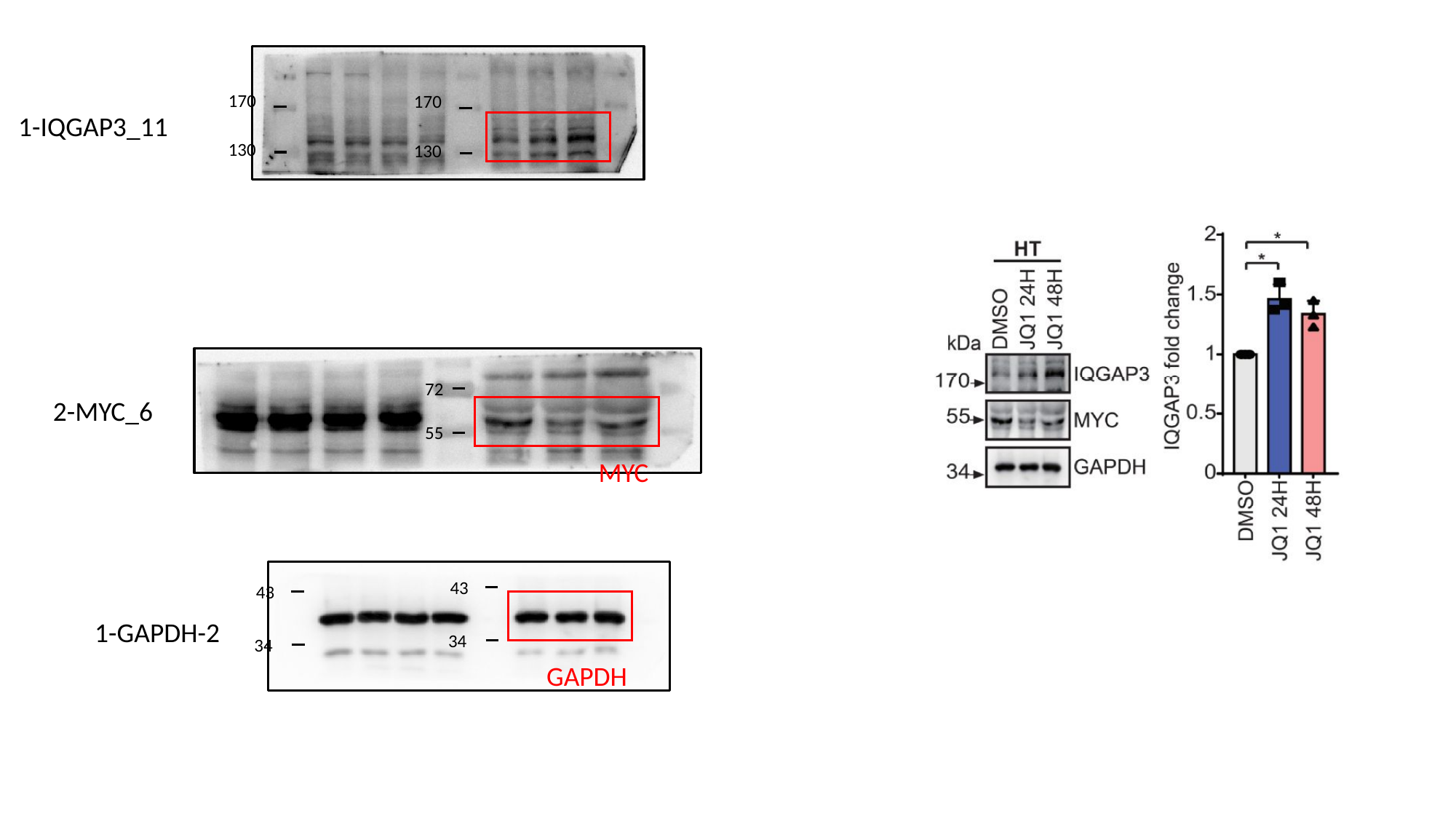

170
170
1-IQGAP3_11
130
130
72
2-MYC_6
55
MYC
43
43
1-GAPDH-2
34
34
GAPDH

## Slide 67
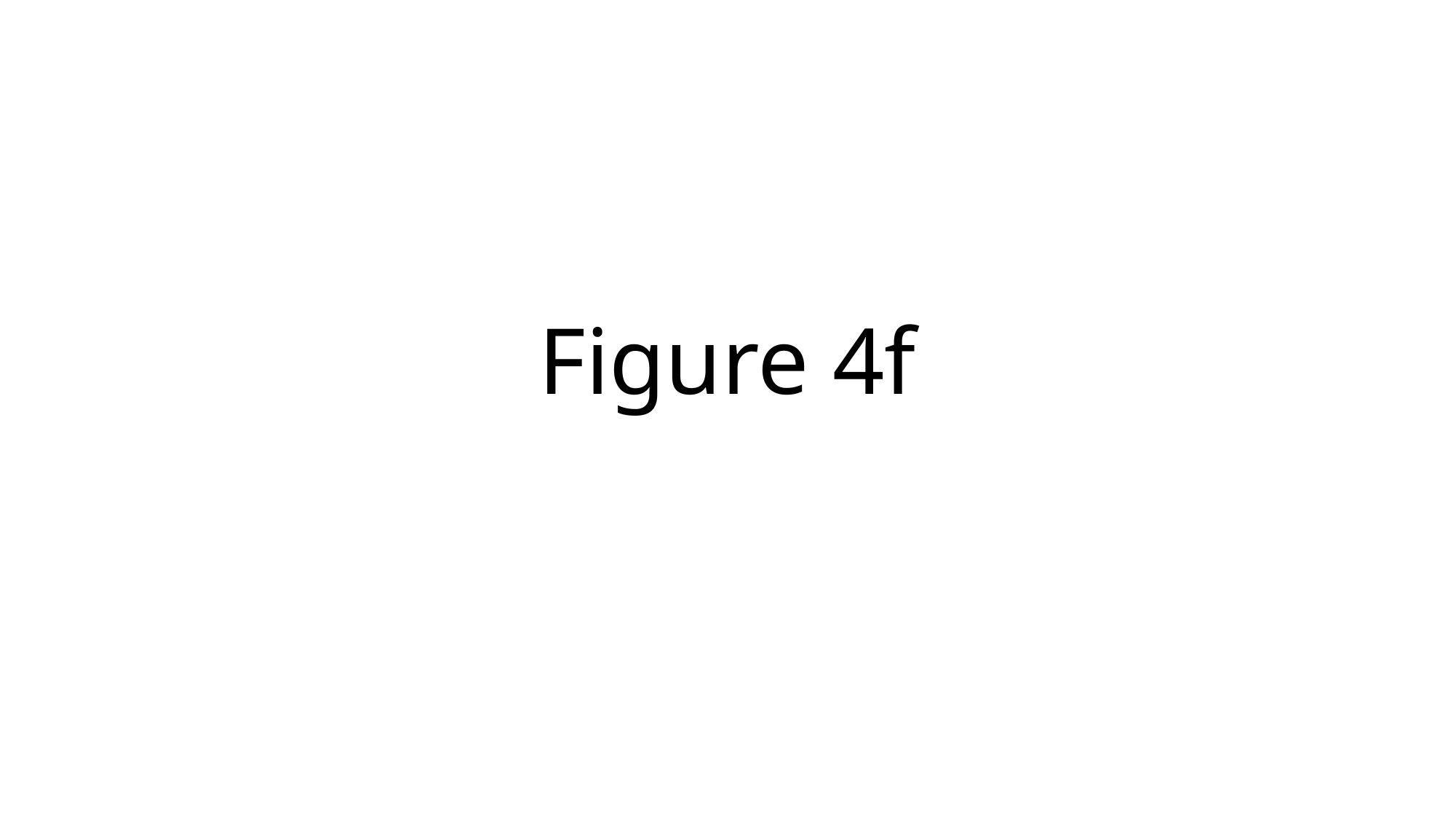

# Figure 4f

## Slide 68
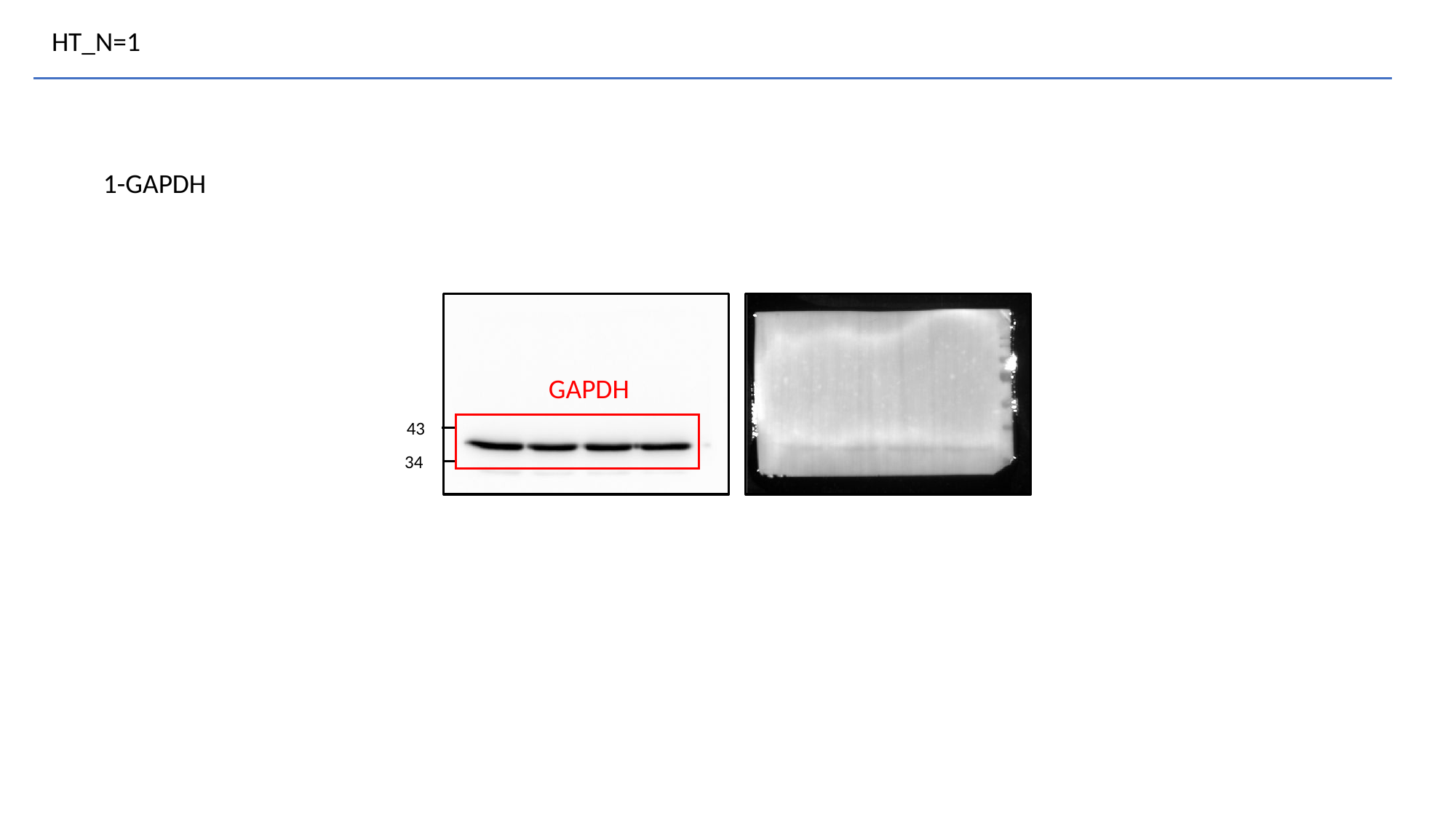

HT_N=1
1-GAPDH
GAPDH
43
34

## Slide 69
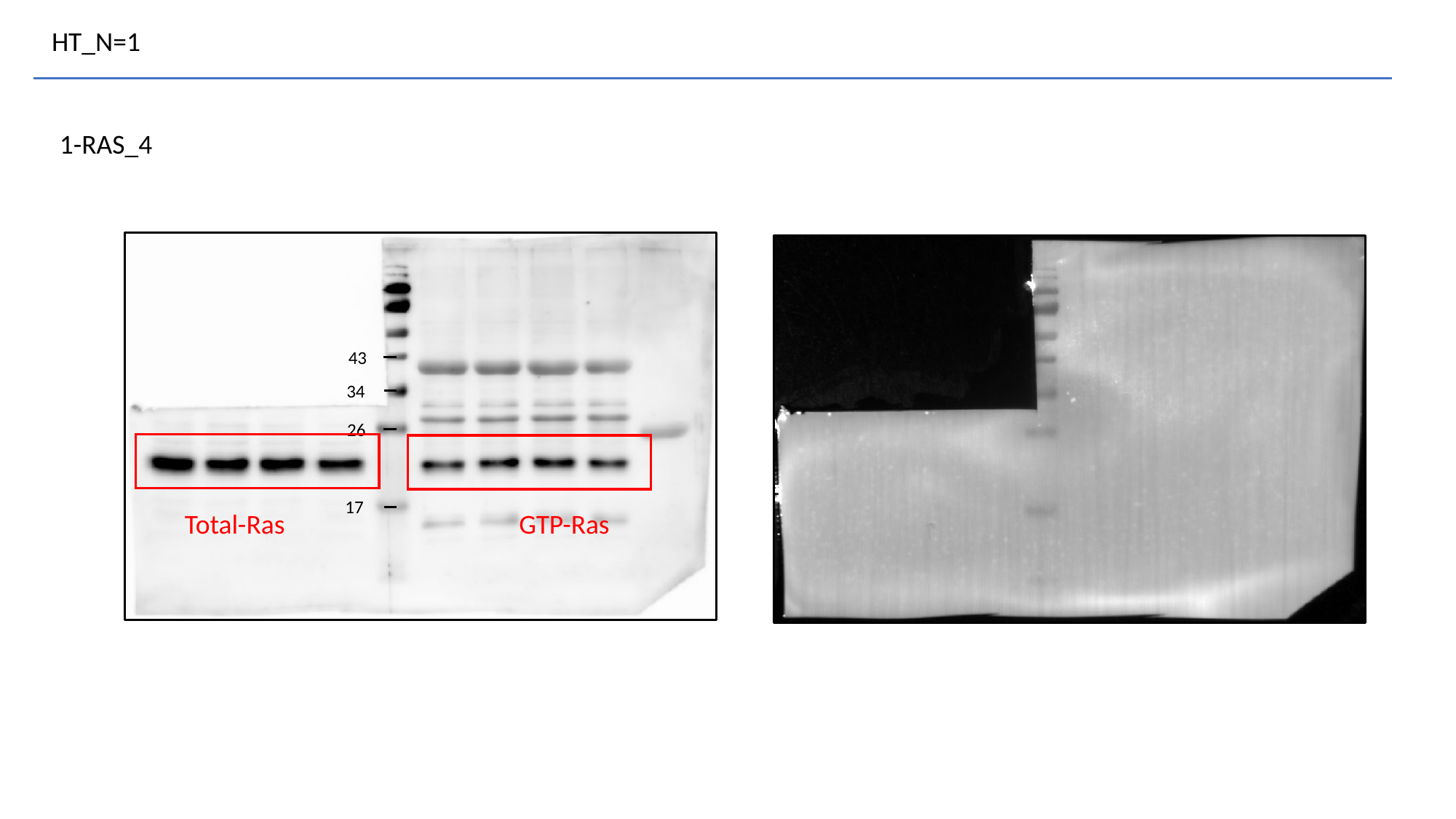

HT_N=1
1-RAS_4
43
34
26
17
Total-Ras
GTP-Ras

## Slide 70
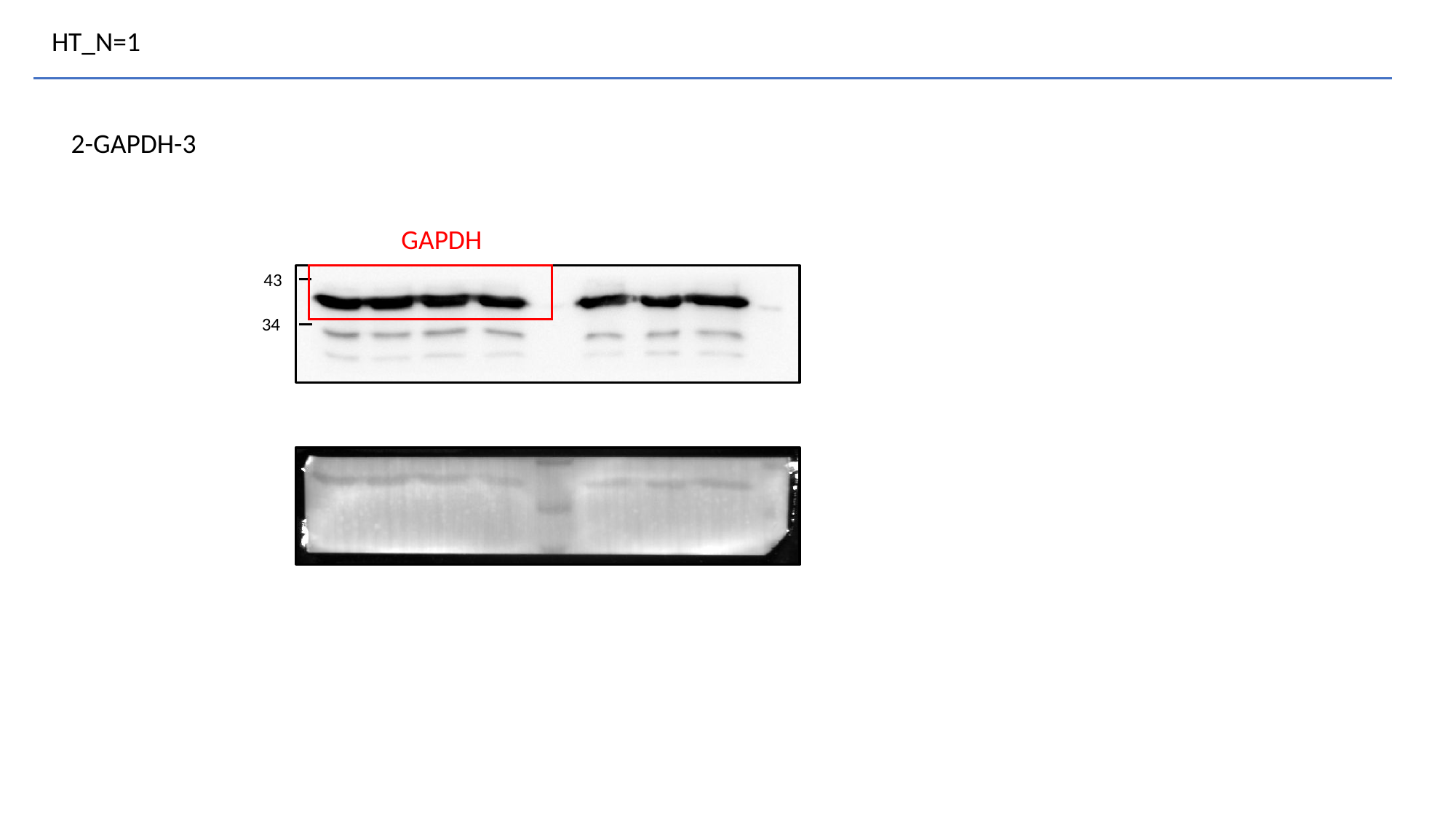

HT_N=1
2-GAPDH-3
GAPDH
43
34

## Slide 71
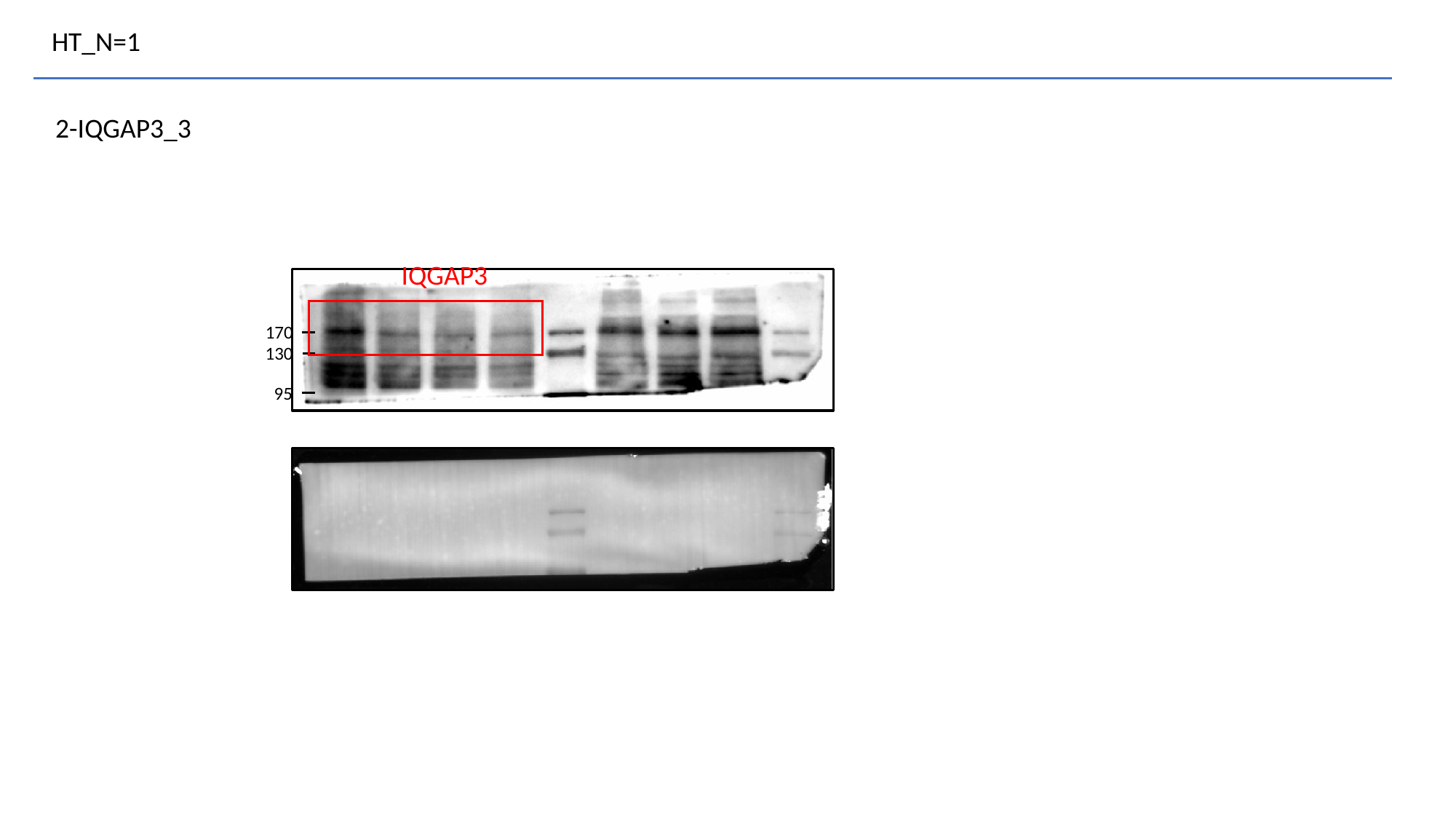

HT_N=1
2-IQGAP3_3
IQGAP3
170
130
95

## Slide 72
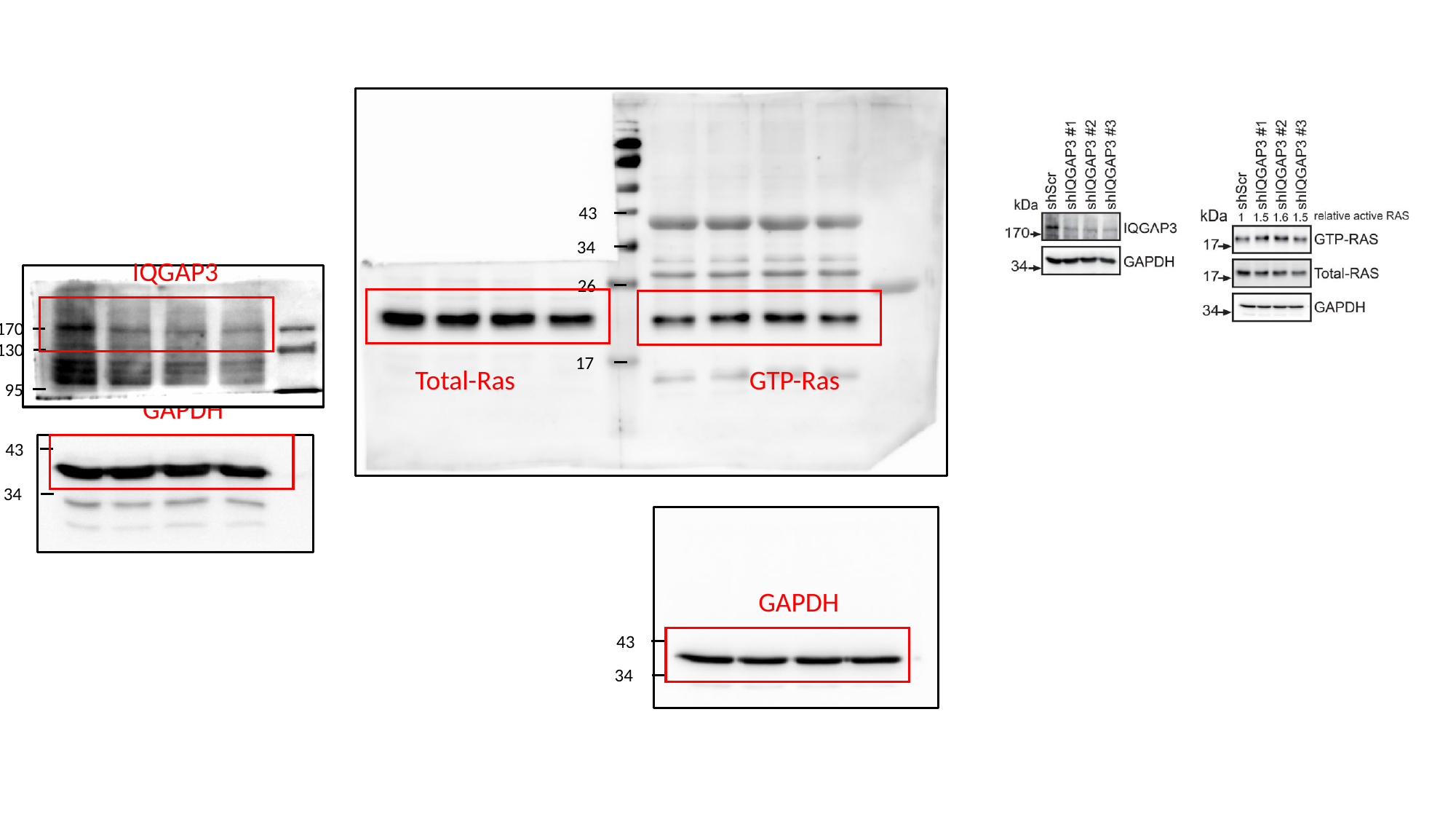

43
34
IQGAP3
26
170
130
17
Total-Ras
GTP-Ras
95
GAPDH
43
34
GAPDH
43
34
