# Supplementary material for: RAS Mediates BET Inhibitor-Endued Repression of Lymphoma Migration and Prognosticates a Novel Proteomics-Based Subgroup of DLBCL through Its Negative Regulator IQGAP3
Source: Cancers (Basel). 2021 Oct 7;13(19):5024. doi: 10.3390/cancers13195024 (PMC8508075; doi:10.3390/cancers13195024)
Supplement: Supplementary file 1 [file cancers-13-05024-s001.zip › cancers 1388839 supplementary figure S1-S5.pdf]

cancers

MDPI

# Supplementary Material: RAS Mediates BET Inhibitor-Endued Repression of Lymphoma Migration and Prognosticates a Novel Proteomics-Based Subgroup of DLBCL Through Its Negative Regulator IQGAP3

**Chih-Cheng Chen, Chia-Chen Hsu, Sung-Lin Chen, Po-Han Lin, Ju-Pei Chen, Yi-Ru Pan, Cih-En Huang, Ying-Ju Chen, Yi-Yang Chen, Yu-Ying Wu and Muh-Hwa Yang**

## Supplementary Methods

*Cell lines*

The cell lines used in this study have been described [1]. The DLBCL cells (SU-DHL-5, HT, Toledo, DB, OCI-Ly3, and U2932) were grown in RPMI medium supplemented with 10% FBS, L-glutamine, penicillin-streptomycin, and sodium pyruvate. The HEK293Ts were grown in DMEM supplemented with 10% FBS and penicillin-streptomycin. The DB, Toledo, and HEK293T cells were purchased from the Bioresource Collections and Research Center of Taiwan. OCI-Ly3 and U2932 cells (originally purchased from Deutsche Sammlung von Mikroorganismen und Zellkulturen, Germany) were obtained from Dr. Chun-Yu Liu (Taipei Veterans General Hospital, Taiwan). The SU-DHL-5 and HT cell lines were provided byfrom Dr. Kung-Chao Chang (National Cheng Kung University, Taiwan). All of the cell lines used in our studies have been tested for Mycoplasma contamination and authenticated by the STR method.

*Trajectory tracking of DLBCL cell migration*

Before starting trajectory tracking, the inhibitor-containing medium was replaced with the inhibitor-free culture medium and then cells were observed in a humidified, $CO_2$ equilibrated chamber with a motorized-stage-equipped Leica DM IRBE microscope (Leica Microsystems, Wetzlar, Germany) for 6 hours. The images were captured every 5 minutes for 6 hours and totally tracked for 73 frames. In stochastically selected high-power fields, we selected individual cells randomly to represent the motility of the plurality of the population using trajectory tracking with ImageJ software. In each independent experiment, 10 cells were counted, and the dates were illustrated with box plots. The cells were not counted when they were out of focus or disappeared in the field during trajectory tracking. Cell motility speed was calculated and is presented as micrometers per minute.

*Plasmids, virus production and infection*

The pCMV-Δ8.9, pMDG, lentiviral expression vectors (pCDH-GFP, pCDH-puro, and pLKO.1-shsScr), and shRNA clones for targeting Myc (TRCN0000039640, and TRCN0000010390) or IQGAP3 (TRCN0000047508, TRCN0000047509, and TRCN0000047510) were purchased from National RNAi Core Facility, Taiwan. The plasmids were co-transfected into HEK293T cells for 16 hours to package the virus. On the Nnext day, the culture medium was renewed and the virus containing supernatant was collected 48 and 72 hours after transfection. For virus transduction, the cells were mixed with virus containing supernatants complemented with 8 μg/mL polybrene (Sigma-Aldrich, St. Louis, MO), and the mixture was centrifuged at 1000g for 120 minutes at 37 °C. After infection with the virus for 24 hours, the infected cells were selected with 1 μg /mL puromycin for three days.

*Small GTPase activity assay and immunoblotting*

For assaying Ras, RhoA, Cdc42, and Rac1 activity, the DLBCL cells were stimulated with additional 10% FBS (total 20%) for 1 day to increase the level of active GTPases. The cells were treated with the indicated inhibitors and were then lysed by 1x Np-40 lysis buffer (1% NP-40, 150 mM NaCl, 1 mM Na3VO4, 50 mM Tris, and 5% Glycerol, pH 7.4), followed by centrifugation at 13,000 x g for 4 min at 4 °C. GTP-bound Ras was pulled down by GST-immobilization Ras binding domain of Raf (Plasmid #13338, Addgene). GTP-bound RhoA was pulled down by GST-immobilization Rho binding domain of Rhotekin. GTP-bound Rac1, cdc42 was pulled down by GST- immobilization p21-activated kinase Rac binding domain of PAK1. All of the effectors (RBD) of small-GTPase were produced in E. coli using lac operon system. The lymphoma cell lysates were incubated with glutathione sepharose beads containing the fusion GST-RBD at 4 °C for 2 hr. The beads were washed once with 1x Np-40 lysis buffer and suspended in SDS-PAGE sample buffer. The whole cell lysates and bound protein were separated by SDS-PAGE on a 12 % polyacrylamide gel, and the blots were probed with monoclonal Antibody for Ras (Ras10, 1:1000 dilution; Thermo Fisher Scientific Inc., Waltham, MA), RhoA (1:500 dilution; Santa Cruz Biotechnology Inc., Dallas, TX), Rac1 (1:1000 dilution; Thermo Fisher Scientific Inc., Waltham, MA), or cdc42 (1:1000 dilution; Thermo Fisher Scientific Inc., Waltham, MA). The antibodies used in this study are listed in Table S1.

*Quantitative RT-PCR (RT-qPCR)*

Total RNA from cell lines were extracted using TRIzol Reagent (Invitrogen Life Technologies, Carlsbad, CA). RNA extracted from cell lines was quantified using a Nanodrop spectrophotometer (Thermo Fisher Scientific Inc., Waltham, MA), and RNA was performed in a total reaction volume of 20 μL containing one microgram of RNA in HiScript I Reverse Transcriptase buffer (Bionovas Biotechnology Co. Ltd., Toronto, Canada) for cDNA synthesis. For real- time RCR, the reaction mixtures (20 μL) contained 10 μL of SYBR Green (Roche Molecular System, Inc., Pleasanton, CA, USA), 0.5 μM forward and reverse primers, and 0.2 μL of cDNA wereas used. The reaction and signal selection were detected by StepOnePlus TM Real-Time PCR System (Thermo Fisher Scientific Inc., Waltham, MA, USA) with two programs: initial denaturation cycle (95 °C for 20 s) and was amplified at 95 °C for 3 s and 60 °C for 30 s total of 40 PCR cycles. The Ggene expression was normalized to GAPDH expression. The primers used in this study are listed in Table S2.

*Flow cytometry analysis of cell cycle distribution*

The HT and SU-DHL-5 cells were seeded in 2 × 106 cells in six6-well culture plates, respectively. The Ccells were treated with the challenged media for 6 and 24 h as indicated in the legend of Supplementary Figure 1. At the end of incubation, the cells were collected together with culture media into 15 mL tubes. The Ccell pellets were harvested by centrifugation, and resuspended in 0.5 mL 1× PBS and then mixed with 75% ethanol at −20°C overnight. Next, the cell pellets were centrifuged and resuspended in propidium iodide (PI) mixture solution (2 mg DNAse-free RNAse A and 0.4 mL of 500 μg/mL PI wereas added to 10 mL of 0.1% Triton X-100 in PBS) at room temperature for 30 min. The density of fluorescence in cell samples was collected (a minimum of 10,000 cells per sample) using the Beckman Coulter CytoFLEX flow cytometer (Beckman Coulter, Brea, CA) and analyzed using the CytExpert software (version 2.2.0.97, Beckman Coulter).

*Mouse experiments*

HT-L1 cells were transfected with piRFP plasmid (plasmid #31857, Addgene, Watertown, MA, USA) to obtain stably expressing near infrared fluorescent reporter (iRFP713) cells (HT-L1-iRFP). For the animal experiment, $1x10^6$ HT-L1-iRFP cells were injected into the splenic parenchyma of the 6-week-old SCID/beige mice. After tumor inoculation for 3 weeks, the mice began to receive intraperitoneal injection of either JQ1 (25 mg/kg, dissolved in 50% ethanol, Figure 1D,F), AZD5153 (25mg/kg, dissolved in 20 %

DMSO, Figure 6C, middle panel), or a combination of AZD5153 (15mg/kg) and copanlisib (1mg/kg, dissolved in 1% trifluoroacetic acid water solution, Figure 6C, right panel). The respective solvent of each inhibitor was used as the control. The intraperitoneal drug/solvent injection was performed twice a week for a total of 9 weeks. Ten minutes after each drug/solvent injection, 0.9% NaCl was injected intraperitoneally to prevent ethanol-induced intestinal perforation or to avert DMSO toxicity. The near infrared fluorescent signals in the mice were detected weekly using a FMT Fluorescence Tomography in vivo Imaging Systems 4000 (PerkinElmer, Inc. Waltham, MA, USA). To eliminate potential background auto-fluorescence at 680nm excitation, mice were supplied with an in vivo imaging diet (Oriental Yeast, Inc, Japan) throughout the experiment. The mice underwent 12-hour fasting before they were subjected to image acquisition.

*High-throughput RNA sequencing (RNA-seq) and bioinformatics analysis*

Total RNA was prepared from SU-DHL-5 and Toledo cells treated with JQ1/vehicle control using TRIzol (Invitrogen, 15596018). Using Illumina HiSeq 2500, 100-bp paired-end RNA-seq was performed by sequencing core facility of Cancer Progression Research Center at National Yang-Ming University. The raw reads were aligned to human reference genome GRCh37/hg19 using CLC Genomics Workbench 11. The transcript levels were expressed as reads per kilobase of transcript per million mapped reads (RPKM) with mRNA information obtained from RefSeq82 using Partek Genomics Suite v.7.0 (Partek, St. Louis, MO). RPKM > 0.1 was defined as expressed mRNAs. Differential expression of mRNAs was analyzed by comparing RPKM and calculated as fold change. The expression profile was compared by hierarchical clustering using dChip software. The differential expressed genes/transcripts were subjected to Gene Ontology classification analysis for canonical pathways analysis. The datasets obtained from the RNA sequencing of the SU-DHL-5 cells with/without JQ1 treatment and Toledo cells with/without JQ1 treatment were deposited in the Gene Expression Omnibus (GEO) database under accession number GSE144821.

*Patients and sample collection*

We retrospectively enrolled 53 patients with DLBCLs who were treated with immunochemotherapy (rituximab, cyclophosphamide, doxorubicin, vincristine, and prednisolone) at our institute. Six healthy adults were also included as control. The study was approved by the Institutional Review Board of Chang-Gung Memorial Hospital (Taiwan) in accordance with the Declaration of Helsinki. The DLBCL samples were supplied in formalin-fixed paraffin-embedded (FFPE) sections, whereas the control samples were collected from fresh buffy coats of healthy donors' peripheral blood that went through the process of positive selection of pan-B cells (EasySep™ HLA Whole Blood B Cell Positive Selection Kit, STEMCELL Technologies, Vancouver, Canada). The demographics of the DLBCL patients is illustrated in Table S3.

*Barcoded mRNA and protein profiling with the nCounter technology*

RNA was extracted using the ReliaPrep™ FFPE kit, and digital gene expression profiling (GEP) was performed to assess the expression of 240 genes using a barcoded profiling method (nCounter technology, NanoString Technologies, Inc., Seattle, WA). The concentrations of RNA were determined by NanoDrop 2000 (Thermo Fisher Scientific Inc., Waltham, MA), and its final working content was adjusted to 80 ng/μl. For quality control, more than 25% of the total RNA fragments in each FFPE-extracted sample must contain at least 300nt length on RNA fragmentation analysis as assessed by Tape Station 2200 (Agilent Technologies, Inc., Santa Clara, CA). Samples not meeting this criterion were considered of low quality and excluded from subsequent nCounter analysis.

The mRNAs analyzed in this study included the nCounter commercial HEME RNA 192-gene panel and a customized 48-gene panel selected by the investigators. After data processing with nSolver4.0, the geometric mean expression of all house-keeping genes

was used as an inter-sample normalization factor and the levels of expression in DLBCL cells were presented in $log_2$(fold changes). The barcoded protein profiling was performed with the nCounter® Vantage 3D™ Protein Panel, which detected 35 target proteins (including phosphorylated proteins), 2 internal controls, and 3 negative controls in one FFPE slide. The antibodies were conjugated with distinct fluorescence barcodes and hybridized to one FFPE slide following the common IHC procedures. After 24 hours, the slides were washed three times to remove the unlabeled antibodies. The protein barcodes were detected through photo-cleavage on the nCounter, and the data were collected using a digital analyzer. In brief, barcodes were counted and tabulated for each target protein. The raw values of protein expression were subtracted by the isotype background and normalized with house-keeping proteins ($\alpha$-tubulin and histone H3). The geometric means of each protein were selected as the basal line comparators for $log_2$(fold changes) calculation. Furthermore, quantification of the phosphorylated proteins was presented in the form of $log_2$[phosphorylation/total form].

*External validation cohort*

GSE11318, a published data set containing transcriptomic information in a cohort of DLBCL patients [2], was retrieved for survival outcome analysis with regard to the expression level of *IGGAP3*. UniGene Hs. 133294 with the probe set 225938_s_at from this array panel, representative of the *IQGAP3* expressional status, were selected for further processing. The probe sequence was found to overlap with the target *IQGAP3* sequence we designed in the nCounter analysis of our samples, indicating concordant detection of *IQGAP3* expressional levels in our patients and in the validation cohort that constituted the GSE11318 data set. Two hundred and three cases were included the validation cohort, but with three patients lost to follow-up, only two hundred cases were subjected to survival outcome analysis. Using the mean *IQGAP3* expression level as the cutoff, we divided these patients into two groups to assess their clinical outcome (Figure 4C, left panel). To mitigate potential confounding effects of samples with an *IQGAP3* level around the mean value on misleadingly impacting the survival outcome, we excluded 78 samples in which the *IQGAP3* levels were within the range of average ± 0.5 x standard deviation. The remaining 122 cases were then stratified into *IQGAP3*-high and -low groups for survival analysis (Figure 4C, right panel).

*Statistics*

Two-tailed, independent Student's t-tests were used to compare continuous variables between two groups. Chi-squared tests were used to compare dichotomous variables. The Kaplan–Meier method and Log-Rank tests were used to compare survival between patient groups. All statistical data were derived from at least two independent biological replicates, and each experiment contained at least two technical replicates. The numerical results are presented as the mean ± S.D. Box-and-whisker plots show the distribution of the data: maximum (upper end of the whisker), upper quartile (top of the box), median (band in the box), lower quartile (bottom of the box), and sample minimum (lower end of the whisker). The horizontal lines in the dot plots represent the mean value of each group. The level of statistical significance was set to $P \leq 0.05$ for all of the tests.

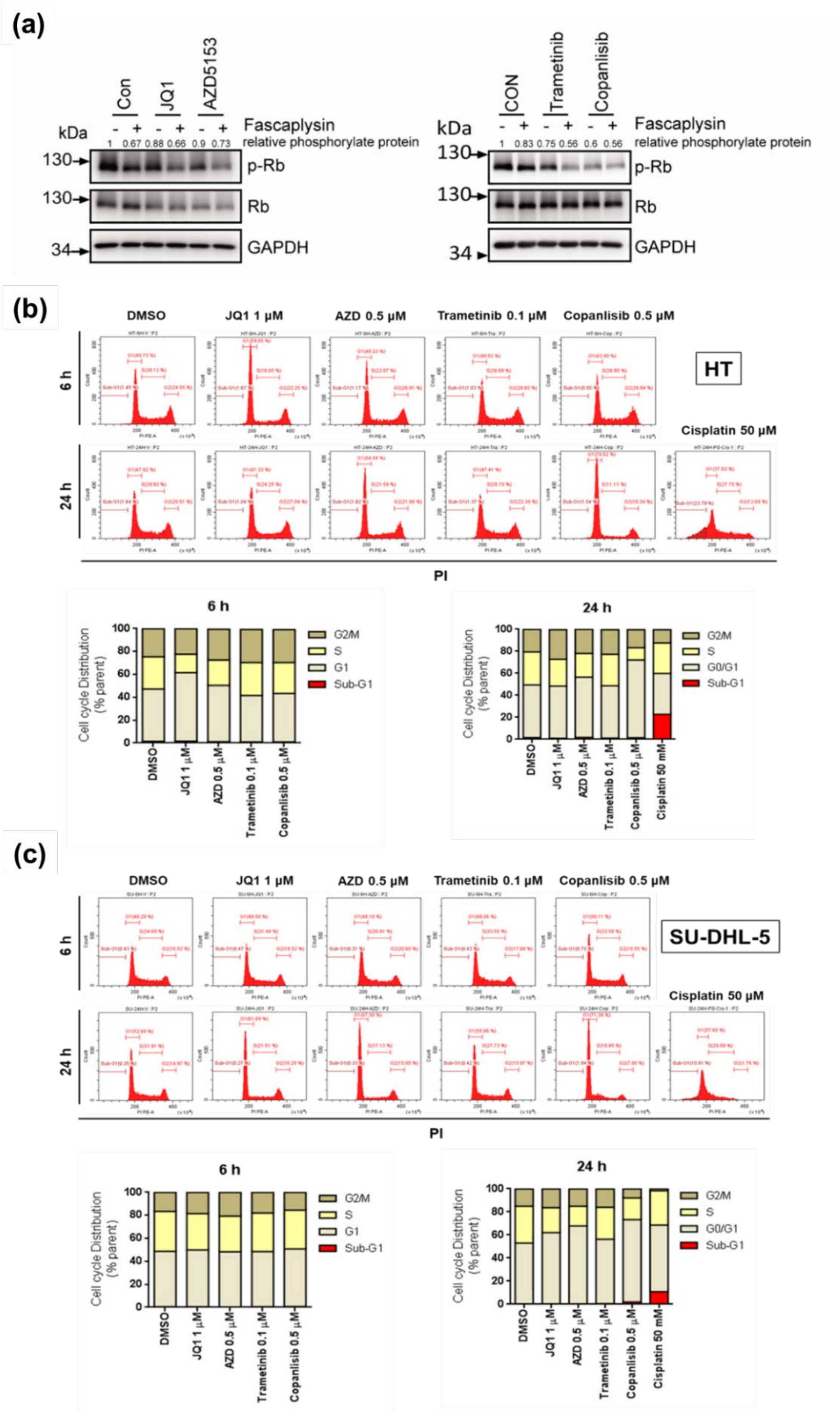


**Figure S1. Sublethal doses of BET, MEK, and PI3K inhibitors on cell cycle propagation. (a)** Western blots demonstrating the degree of Rb protein phosphorylation. Cell lysates from HT cells treated with DMSO, JQ1 (1 μM), AZD5153 (0.5 μM), trametinib (0.1 μM), or copanlisib (0.5 μM) in the presence or absence of a specific CDK4 inhibitor, fascaplysin (0.2 μM), were harvested and subjected to further analysis. GAPDH was used as a loading control. **(b-c)** Cell cycle analysis with flow cytometry. HT (B) and SU-DHL-5 (c) cells treated with DMSO or various inhibitors were permeabilized, stained with propidium iodide (PI), and subjected to flow cytometric analysis. Cells treated with DMSO and cisplatin (50 μM) for 24 hours were used as negative and positive controls, respectively. By calculating the percentages of cells in sub-G1, G1, S, and G2/M phases, cell cycle distribution was summarized into vertical slices and depicted in the respective lower panels.

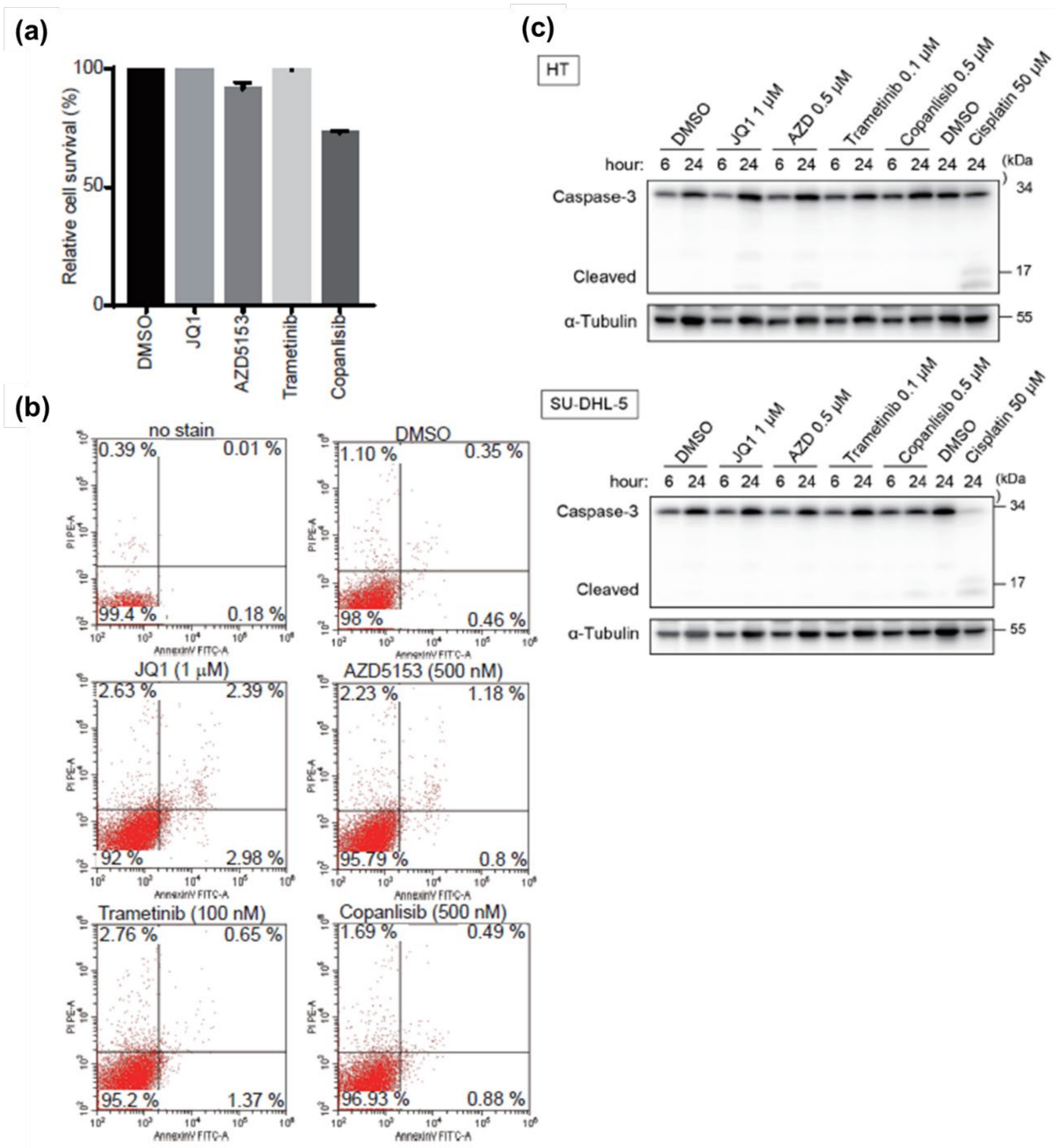


**Figure S2. Sublethal doses of BET, MEK, and PI3K inhibitors on apoptosis and cellular viability. (a)** Effects of BET, MEK, and PI3K inhibitors on cellular viability. HT cell, grown overnight in collagen gels, were treated with either DMSO as the control or 1 μM JQ1, 0.5 μM AZD5153, 0.1 μM trametinib, and 0.5 μM copanlisib for 6 hours. Cells were then incubated with an MTT reagent for viability assay. **(b)** Flow cytometric analysis of apoptosis in inhibitor-treated DLBCL cells. Cells were subjected to Annexin V and PI assays 6 hours after treatment with indicated drugs. **(c)** Western blotting of full-length or cleaved Caspase 3 after inhibitor treatment. HT and SU-DHL-5 cells were treated with inhibitors as described above for 6 or 24 hours and subjected to protein quantification. ⊚-tubulin was used as a loading control. Cells treated with DMSO and cisplatin (50 μM) for 24 hours were used as negative and positive controls, respectively.

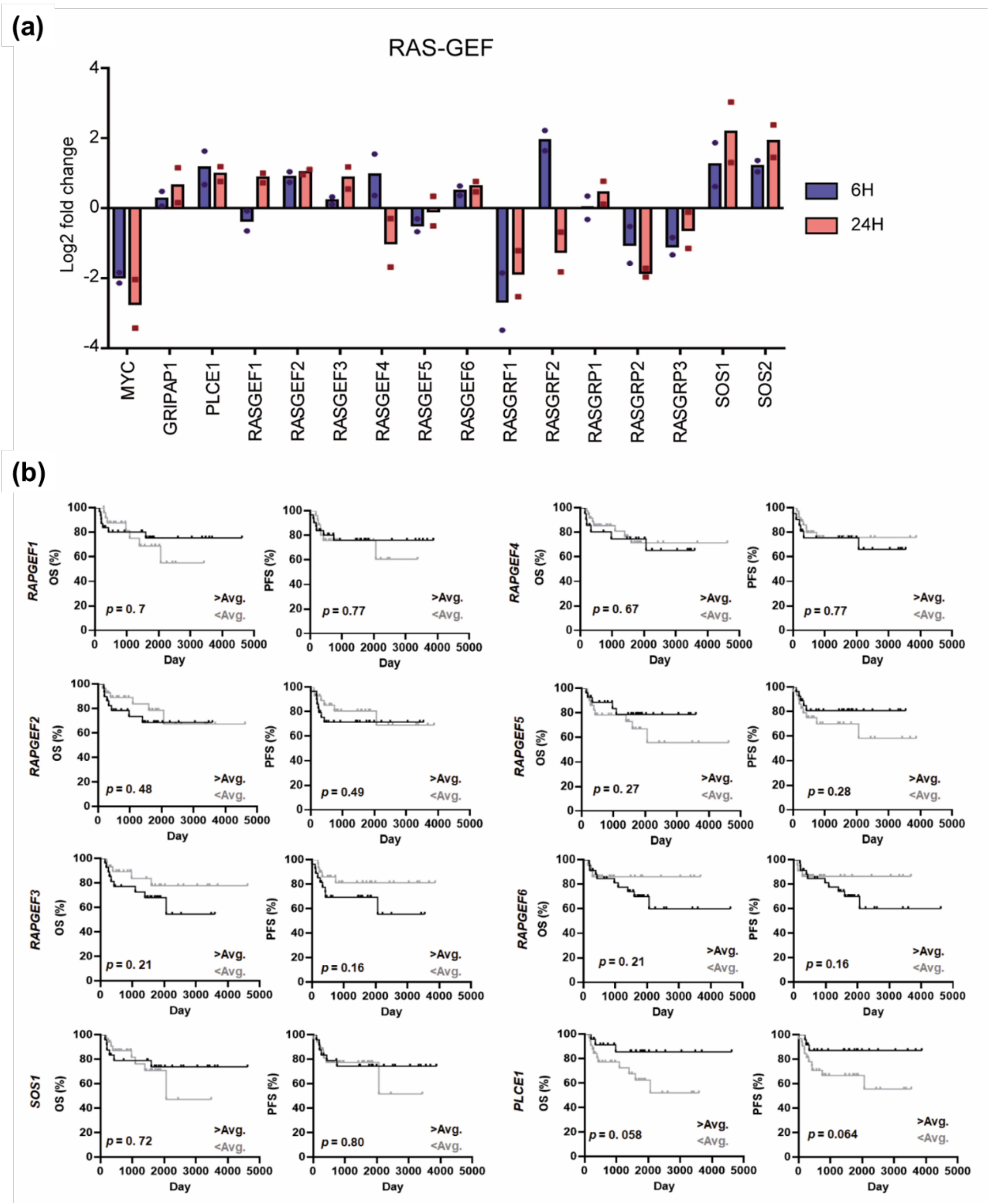


**Figure S3. Effects of BET inhibition on RASGEF genes and their prognostic impacts on survival outcome. (a)** Relative expression of RASGEF genes in HT cells treated with JQ1. Cells were treated with 1 ◎M of JQ1 for indicated time periods and the expression levels were assessed by qPCR. Folds of expression were obtained through calculating the ratios between treated samples and untreated controls. **(b)** The prognostic impacts of selected RASGEF genes on the clinical outcome. Patients with DLBCL were stratified into high- (>average) and low- (<average) expressor groups of distinct RASGEF genes. Log-Rank test was employed to assess the prognostic significance of individual genes on progression-free survival (PFS) and overall survival (OS).

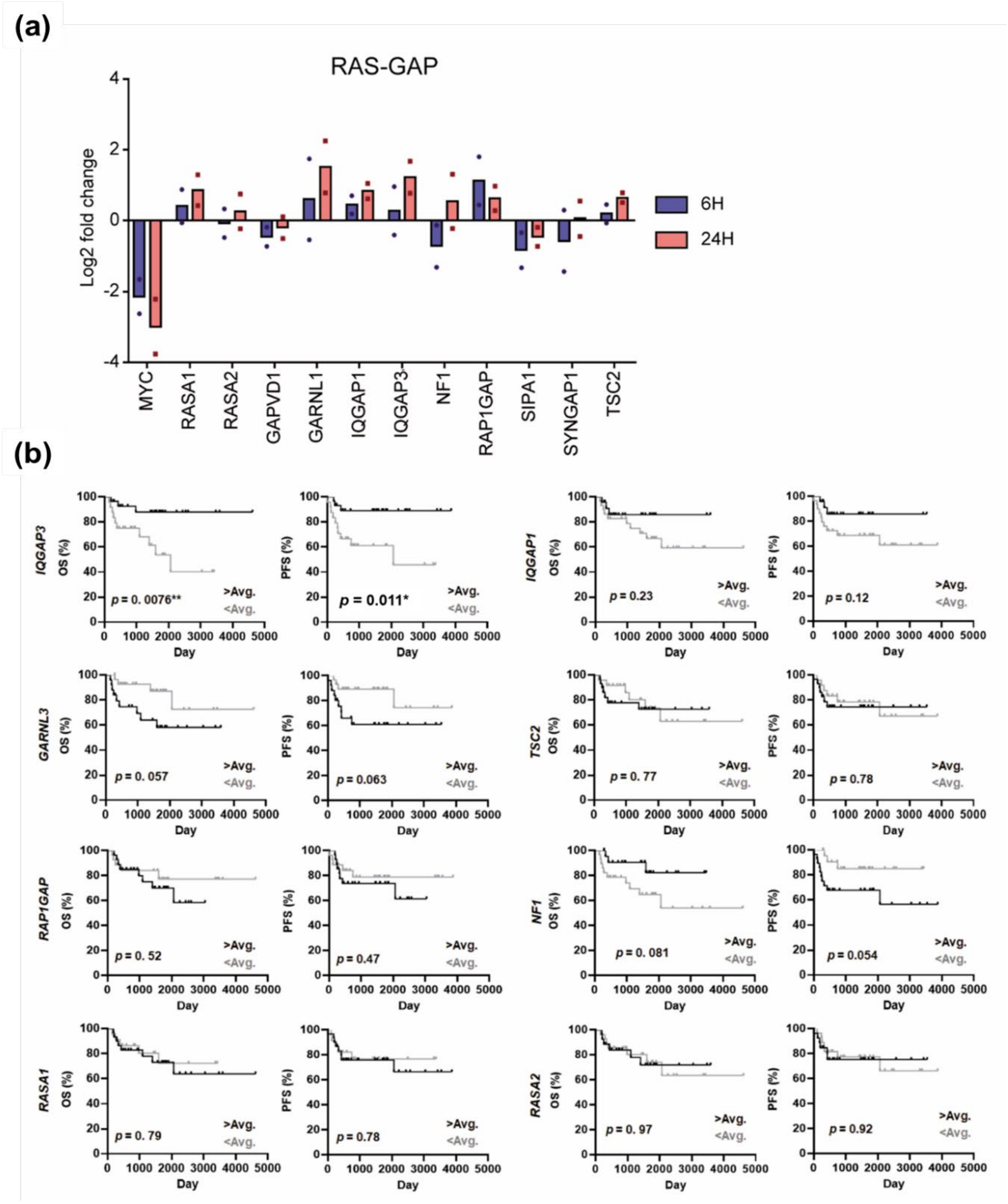


**Figure S4. Effects of BET inhibition on RASGAP genes and their prognostic impacts on survival outcome. (a)** Relative expression of RASGAP genes in HT cells treated with JQ1. Cells were treated with 1 ◉M of JQ1 for indicated time periods and the expression levels were assessed by qPCR. Folds of expression were obtained through calculating the ratios between treated samples and untreated controls. **(b)** The prognostic impacts of selected RASGAP genes on the clinical outcome. Patients with DLBCL were stratified into high- (>average) and low- (<average) expressor groups of distinct RASGAP genes. Log-Rank test was employed to assess the prognostic significance of individual genes on progression-free survival (PFS) and overall survival (OS).

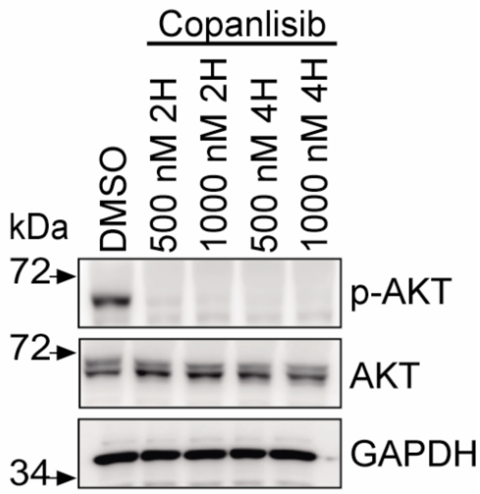


**Figure S5. Validation of suppressing AKT activation by the PI3K inhibitor copanlisib.** Western blots demonstrating the effect of PI3K inhibitor copanlisib on downstream phospho-AKT (p-AKT) activity. Cell lysates from HT cells incubated with copanlisib at 0.5 and 1.0 μM for 2 or 4 hours were harvested and subjected to further analysis. GAPDH was used as a loading control.

## Reference


1. Pan, Y.R.; Chen, C.C.; Chan, Y.T.; Wang, H.J.; Chien, F.T.; Chen, Y.L.; Liu, J.L.; Yang, M.H. STAT3-coordinated migration facilitates the dissemination of diffuse large B-cell lymphomas. *Nature communications* **2018**, *9*, 3696, doi:10.1038/s41467-018-06134-z.
2. Lenz, G.; Wright, G.W.; Emre, N.C.; Kohlhammer, H.; Dave, S.S.; Davis, R.E.; Carty, S.; Lam, L.T.; Shaffer, A.L.; Xiao, W.; et al. Molecular subtypes of diffuse large B-cell lymphoma arise by distinct genetic pathways. *Proceedings of the National Academy of Sciences of the United States of America* **2008**, *105*, 13520-13525, doi:10.1073/pnas.0804295105.
